# Conserved Molecular Mechanism of TyrA Dehydrogenase Substrate Specificity Underlying Alternative Tyrosine Biosynthetic Pathways in Plants and Microbes

**DOI:** 10.3389/fmolb.2017.00073

**Published:** 2017-11-07

**Authors:** Craig A. Schenck, Yusen Men, Hiroshi A. Maeda

**Affiliations:** Department of Botany, University of Wisconsin-Madison, Madison, WI, United States

**Keywords:** L-Tyrosine, primary metabolism, natural products, aromatic amino acid, substrate specificity

## Abstract

L-Tyrosine (Tyr) is an aromatic amino acid synthesized *de novo* in plants and microbes. In animals, Tyr must be obtained through their diet or synthesized from L-phenylalanine. In addition to protein synthesis, Tyr serves as the precursor of neurotransmitters (e.g., dopamine and epinephrine) in animals and of numerous plant natural products, which serve essential functions in both plants and humans (e.g., vitamin E and morphine). Tyr is synthesized via two alternative routes mediated by a TyrA family enzyme, prephenate, or arogenate dehydrogenase (PDH/TyrA_p_ or ADH/TyrA_a_), typically found in microbes and plants, respectively. Although ADH activity is also found in some bacteria, the origin of arogenate-specific TyrA_a_ enzymes is unknown. We recently identified an acidic Asp222 residue that confers ADH activity in plant TyrAs. In this study, structure-guided phylogenetic analyses identified bacterial homologs, closely-related to plant TyrAs, that also have an acidic 222 residue and ADH activity. A more distant archaeon TyrA that preferred PDH activity had a non-acidic Gln, whose substitution to Glu introduced ADH activity. These results indicate that the conserved molecular mechanism operated during the evolution of arogenate-specific TyrA_a_ in both plants and microbes.

## Introduction

L-Tyrosine (Tyr) is an aromatic amino acid required for protein synthesis in all organisms, but synthesized *de novo* in plants and microbes. Thus, in animals Tyr must be acquired through the diet or produced from L-phenylalanine (Phe) by Phe-hydroxylase (Fitzpatrick, 1999). In addition to protein synthesis, Tyr is used to synthesize animal neurotransmitters, such as dopamine and epinephrine (adrenaline) (Fernstrom and Fernstrom, 2007) and melanin skin pigments (Slominski et al., 2004). Tyr also serves as the precursor to numerous plant natural products with diverse functions such as electron carriers (e.g., plastoquinone and ubiquinone; Millner and Barber, 1984), defense (e.g., dhurrin and rosmarinic acid; Petersen, 2013; Gleadow and Møller, 2014), and pollinator attraction (e.g., betalain pigments; Gandía-Herrero and García-Carmona, 2013). Some of these natural products also serve medicinal and nutritional roles in humans such as antioxidants (vitamin E; Falk and Munné-Bosch, 2010), and analgesics (e.g., morphine; Sato et al., 2007).

**Figure 1:**
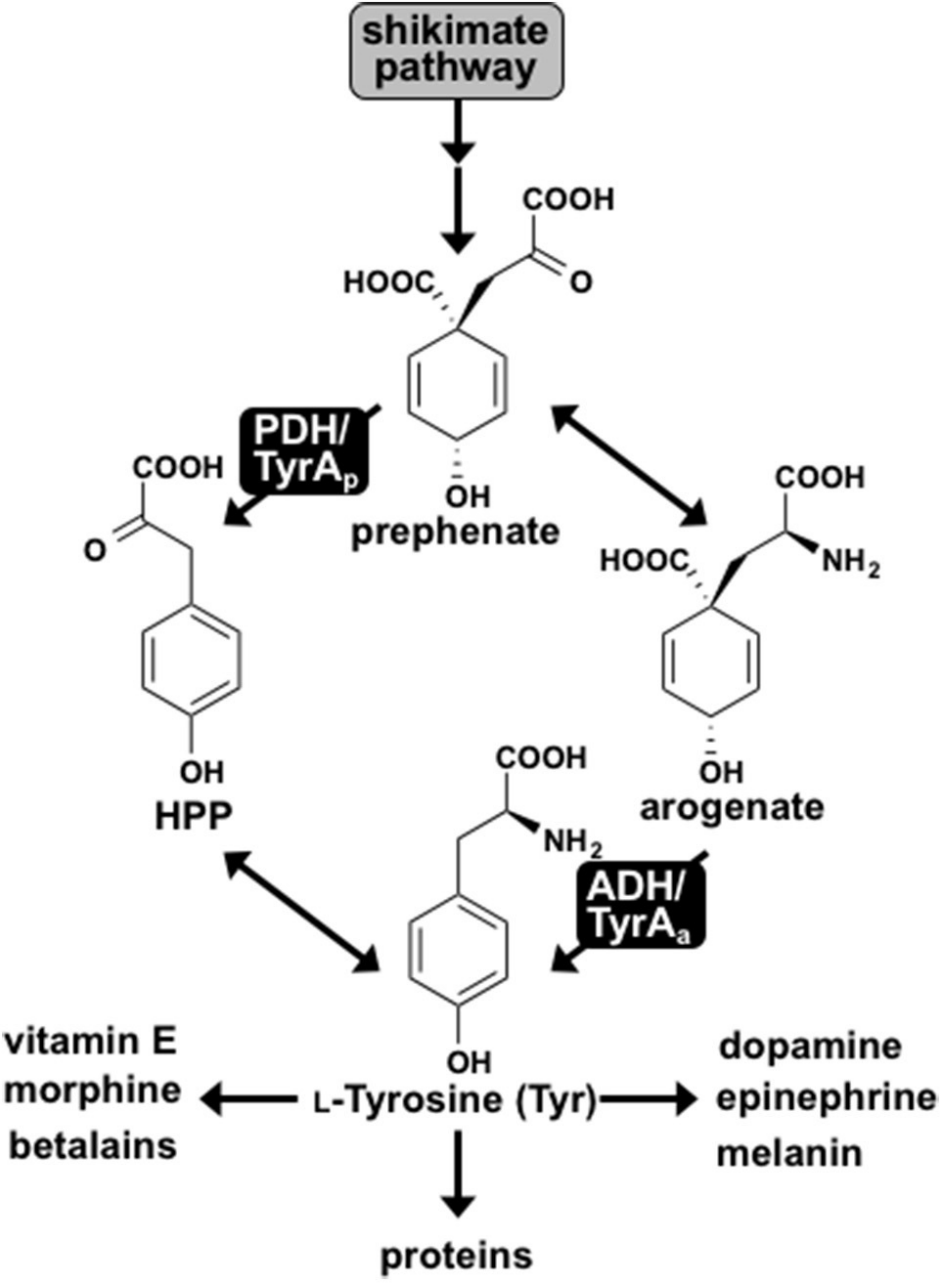
L-Tyrosine biosynthesis via two pathways in plants and microbes. Two pathways for Tyr biosynthesis from prephenate downstream of the shikimate pathway are shown. The PDH/TyrA_p_ pathway is present in most microbes, whereas the ADH/TyrA_a_ pathway is ubiquitous in plants. Tyr serves as the precursor for protein synthesis and many downstream metabolites in plants and humans.

Tyr is synthesized from prephenate, downstream of the shikimate pathway, by two alternative routes. In most microbes prephenate is first converted into 4-hydroxyphenylpyruvate (HPP) by a NAD^+^-dependent prephenate-specific TyrA dehydrogenase (PDH/TyrA_p_), followed by transamination to form Tyr (Figure 1) (Jensen and Pierson, 1975; Bentley, 1990). In contrast, plants first transaminate prephenate to form arogenate, which is converted to Tyr by a NADP^+^-dependent arogenate-specific TyrA dehydrogenase (ADH/TyrA_a_) (Gaines et al., 1982; Connelly and Conn, 1986; Rippert and Matringe, 2002). Plant TyrA_a_ and microbial TyrA_p_ catalyze the key regulatory step in Tyr biosynthesis, and their substrate specificity defines the Tyr biosynthetic routes via arogenate and prephenate intermediate, respectively (Figure 1).

Notably, exceptions have been reported for the typical cofactor and substrate specificities of TyrA dehydrogenases in plants and microbes. Some microbes, for example, use NADP^+^ cofactor instead of NAD^+^ (Fazel et al., 1980; Subramaniam et al., 1994). Arogenate-specific TyrA_a_ enzymes have also been identified in some microbes, such as the α-proteobacteria *Zymomonas mobilis* and *Phenylobacterium immobile* (Mayer et al., 1985; Zhao et al., 1993). While all plants investigated have arogenate-specific TyrA_a_, the legume family additionally possesses prephenate-specific TyrA_p_ enzymes (Gamborg and Keeley, 1966; Rubin and Jensen, 1979; Schenck et al., 2015). Using the unique presence of TyrA_p_ in legumes combined with structural analyses, a single acidic residue in the active site, Asp222, was recently shown to confer arogenate substrate specificity of plant TyrAs by directly interacting with the side chain amine of arogenate substrate (Schenck et al., 2017), which is absent in prephenate (Figure 1). Indeed, mutating Asp222 of diverse plant TyrA_a_ into the corresponding Asn residue in legume TyrA_p_ reduced their ADH activity and introduced PDH activity, suggesting that Asn222 played a key role in the recent evolution of legume-specific TyrA_p_ (Schenck et al., 2017). However, the early evolutionary origin and mechanism of microbial and plant TyrA_a_ enzymes are still unresolved.

Here, we used the Asp222 residue to trace the evolutionary history of TyrA_a_ enzymes in deep taxonomic lineages across Plantae and microbes. Structure-guided, phylogenetic analyses combined with biochemical characterization show that microbial TyrA orthologs closely-related to plant TyrA_a_ also have a corresponding Asp (or Glu) residue and prefer ADH activity. Contrarily, microbial TyrA, which are more distantly-related to plant TyrAs, contained a non-acidic Gln or Asn and preferred PDH activity. Furthermore, site-directed mutagenesis of an acidic Asp into a neutral Asn on a spirochaetes TyrA_a_ reduced ADH activity, while introducing PDH activity. The reciprocal mutation of Gln into an acidic Glu on an archaeon TyrA_p_ reduced PDH and introduced ADH activity. These data suggest that plants and some microbial TyrA orthologs share an evolutionarily conserved substrate specificity mechanism, and that acquisition of the key active site acidic residue was crucial in evolution of arogenate-specific TyrA_a_ enzymes in plants and closely-related microbes.

## Materials and methods

### Identification of microbial TyrA orthologs

**Figure 2:**
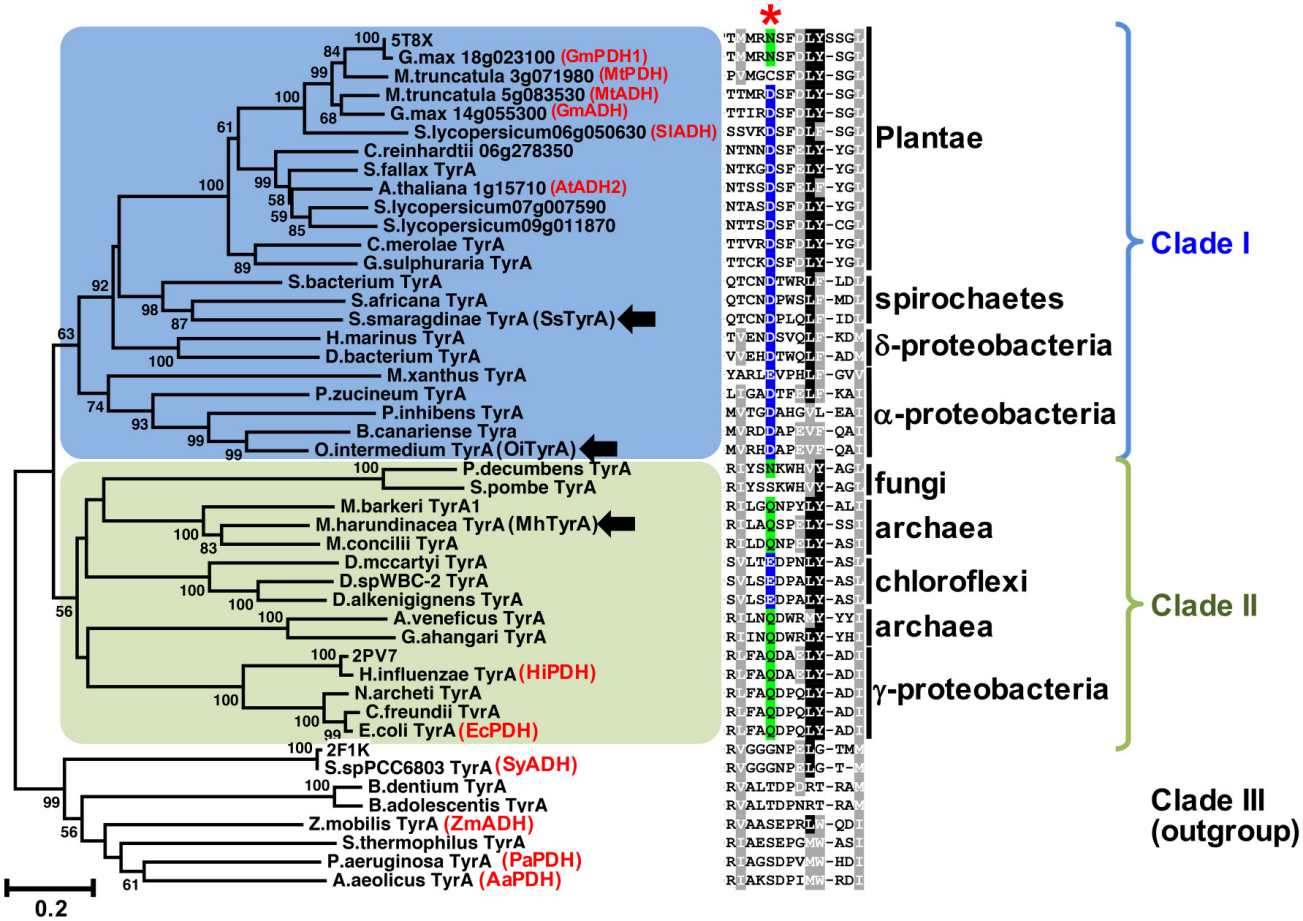
The conserved acidic residue at 222 among clade I TyrA orthologs from plants, algae, and closely-related bacteria. Structure-guided phylogenetic analysis of plant and microbial TyrAs. Three distinct clades are formed; clade I contains all plant TyrAs and closely-related microbes (blue), clade II contains bacteria, archaea, and fungi TyrAs (green), and clade III, which was used as an outgroup. Enzymes characterized in this study are marked by black arrows. Structures used to guide the alignment are labeled with their PDB IDs. Previously characterized TyrAs are labeled in red with their preferred PDH or ADH activity. Scale bar represents number of substitutions per branch length. A trimmed amino acid alignment of corresponding sequences shows a conserved acidic residue (Asp or Glu, highlighted in blue) among clade I, which is replaced with a non-acidic Asn or Gln residue (highlighted in green) in most clade II at the corresponding 222 position marked with a red star. Identical amino acids present in >50%, black shading; biochemically similar residues present in >50% of the sequences, gray shading.

BlastP searches were performed using the amino acid sequences of previously characterized TyrA homologs from plants [soybean PDH; GmPDH1 (Schenck et al., 2015) and Arabidopsis ADH; AtADH2 (Rippert and Matringe, 2002)] and microbes [*Synechocystis* sp. PCC6803 ADH (Legrand et al., 2006), and *E. coli* PDH (Hudson et al., 1984)] as the query in the NCBI database. This yielded only closely-related plant and microbial TyrA orthologs (e.g., algae and, γ-proteobacteria), which were then used as the query to perform additional BlastP searches. Every 5th BlastP hit was selected to provide sequences from various microbial lineages and limit bias in sample selection. Data S1 contains all the sequence information for the TyrA orthologs used in Figure 2 and Figure S1. A structure-guided amino acid alignment was performed in PROMALS3D (Pei and Grishin, 2007) using the default parameters with structures of TyrA enzymes from plants and microbes with varying substrate specificities (*G. max* TyrA_p_; GmPDH1; PDB # 5T8X, *H. influenzae* TyrA_p_; HiPDH; 2PV7, and *Synechocystis* sp. PCC6803 TyrA_a_; SynADH; PDB # 2F1K). The amino acid alignment from PROMALS3D was used to construct phylogenetic trees using MEGA7 (Kumar et al., 2016). The full amino acid alignment can be found in Data S2. The analyses involved 130 amino acid sequences and all sites with <75% coverage were eliminated from the analysis. A neighbor-joining method (Figure S1A; Saitou and Nei, 1987) was used to estimate evolutionary history using 1,000 bootstrap replicates (values shown at branches). The tree in Figure 2 is a representative tree using a subset of the sequences found in Figure S1. Additional phylogenetic analyses were performed using the Maximum Likelihood method based on the Jones-Taylor-Thornton (JTT) matrix-based model (Jones et al., 1992), which gave overall similar results (Figure S1B). All phylogenetic trees are drawn to scale, with branch lengths measured in the number of substitutions per site.

### Recombinant protein expression and purification and site directed mutagenesis

Full length coding sequences from *Ochrobactrum intermedium* LMG 3301 (EEQ93947.1; OiTyrA), *Sediminispirochaeta smaragdinae* DSM 11293 (ADK80640.1; SsTyrA), and *Methanosaeta harundinacea* (KUK94425.1; MhTyrA) were codon optimized for expression in *E. coli*, gene synthesized (Biomatik), and inserted into pET28a vector using *Eco*R1 and *Nde*1 sites in frame with an N-terminal 6x-His tag using a previously described cloning method (Schenck et al., 2015).

For site directed mutagenesis, plasmid template was diluted 100-fold, mixed with 0.04 U/μL Phusion DNA polymerase (Thermo), 0.2 mM dNTP's, 0.5 μM forward and reverse mutagenesis primers and 1x Phusion reaction buffer (Thermo), and then placed in a thermocycler for 98°C for 30 s followed by 20 cycles of 10 s at 98°C, 20 s at 70°C, 4.5 min at 72°C with a final extension at 72°C for 10 min. The sequence of primers used for mutagenesis were (5′-CATTCTGGCC*GAA*AGCCCGGAACTGTATAGTAGC-3′) and (5′-GTTCCGGGCT*TTC*GGCCAGAATGCGGCCCACAAAATC-3′) for MhTyrA and (5′-GTAAC*AAT*CCACTTCAGCTGTTTATAGACTTGCAAC-3′) and (5′-CTGAAGTGG*ATT*GTTACACGTTTGTTCGCGCACCTG-3′) for SsTyrA (mutated codons are italicized). The PCR products were purified with QIAquick Gel Extraction Kit (Qiagen), treated with DpnI (Thermo) to digest methylated template DNA for 30 min at 37°C, and then transformed into *E. coli* XL1-Blue cells. Plasmids were sequenced to confirm that no errors were introduced during PCR and cloning.

For recombinant protein expression, *E. coli* Rosetta2 (DE3) cells (Novagen) transformed with the above plasmids were cultured as previously reported (Schenck et al., 2017). For protein purification, 20 mL of the *E. coli* supernatant expressing the appropriate plasmid was applied to a 1 mL HisTrap FF column for purification of the His-tagged recombinant protein using an ÄKTA FPLC system (GE Healthcare). After loading the supernatant, the column was washed with 20 column volumes of 90% buffer A (0.5 M NaCl, 0.2 M sodium phosphate, and 20 mM imidazole) and 10% buffer B (0.5 M NaCl, 0.2 M sodium phosphate, and 0.5 M imidazole) followed by elution with 100% buffer B. Fractions containing purified recombinant enzymes were pooled and desalted by Sephadex G50 column (GE Healthcare) size-exclusion chromatography into lysis buffer (Schenck et al., 2017). The purity of purified proteins were analyzed by SDS-PAGE using ImageJ software (Schneider et al., 2012). All protein purification steps were performed at 4°C unless stated otherwise.

### ADH and PDH assays

ADH and PDH assays were performed using purified recombinant enzymes for SsTyrA Wild-type (Wt) and D208N mutant, and MhTyrA Wt and Q227E mutant, while the *E. coli* cell lysate was used for OiTyrA as expression and purification of this enzyme was unsuccessful. Reactions contained 0.8 mM substrate (arogenate or prephenate) and 0.8 mM cofactor (NADP^+^ or NAD^+^) together with reaction buffer [25 mM HEPES pH 7.6, 50 mM KCl, 10% (v/v) ethylene glycol]. For OiTyrA assays containing cell lysates, reactions were incubated for 45 min and analyzed using HPLC as previously reported (Schenck et al., 2015). For pure enzymes, reactions were monitored every 10–15 s for reduced cofactor at A_340nm_ using a microplate reader (Tecan Genios) in a reaction volume of 30 μL. Kinetic parameters of purified recombinant enzymes were determined from assays containing varying concentrations of arogenate (39.1 μM−5 mM) or prephenate (46.9 μM−6 mM) substrate, 0.8 mM of the preferred cofactor and monitored every 10–15 s for production of reduced cofactor at A_340nm_ using a microplate reader (Tecan Genios). Kinetic parameters were determined by fitting initial velocity data to the Michaelis–Menten equation using Origin software (OriginLab) from technical replicate assays (*n* = 3). Arogenate substrate was prepared by enzymatic conversion of prephenate (Sigma-Aldrich) as previously reported (Maeda et al., 2010). Enzyme assays were quantified using the A_340nm_ of a standard curve of reduced cofactor (NADPH or NADH) and activity is expressed as Kat/mg (moles of product produced per second per mg protein). All enzyme assays were conducted at a reaction time and protein concentration that were in the linear range and proportional to reaction velocity.

### Modeling microbial TyrA enzymes

Homology models were made using SWISS-MODEL (Biasini et al., 2014) with default parameters to predict the structures of divergent TyrA enzymes. Enzymes that are more closely-related to plants (e.g., SsTyrA and MhTyrA) were modeled using the GmPDH1 structure as the template, though this resulted in a poor model for BdTyrA, which falls within the outgroup. BdTyrA was additionally modeled using *Synechocystis* sp. PCC6803 ADH. Homology models were visualized using PyMOL.

## Results

### Phylogenetic relationship of plant and microbial TyrAs

Previous studies suggested that plant TyrAs are not derived from an eukaryotic ancestor or through cyanobacterial endosymbiosis because they are most similar to other microbes including some proteobacteria (Bonner et al., 2008; Reyes-Prieto and Moustafa, 2012; Dornfeld et al., 2014; Schenck et al., 2017); however, their precise origin was unclear. To resolve the phylogenetic relationship of TyrA orthologs from divergent organisms including plants and microbes, here we performed structure-guided phylogenetic analyses using PROMALS3D to achieve alignment of TyrA orthologs with low sequence similarities (see section Materials and Methods; Pei and Grishin, 2007). Three distinct clades were identified that contain: plant TyrAs together with those from algae, spirochaetes, α- and δ-proteobacteria (**clade I**, shaded blue in Figure 2, Figure S1), TyrA orthologs from some archaea, fungi, γ-proteobacteria, and chloroflexi (**clade II**, shaded green), and TyrA orthologs from various microbes, which formed the outgroup and contains previously characterized microbial TyrA orthologs from *Synechocystis* sp. PCC 6803 and *Aquifex aeolicus* having very low sequence similarity (~30%) to plant TyrAs (**clade III**, Figure 2, Figure S1). Interestingly, TyrAs from some spirochaetes lineages (some of which are known to cause harmful human diseases like Lyme disease; Pritt et al., 2016) formed a subclade with plant and algae TyrAs within clade I using various phylogenetic methods (Figure 2, Figure S1). These data suggest that Plantae TyrA may have been acquired through horizontal gene transfer (HGT) from an ancestor of one of these closely-related microbes.

### Microbial TyrA orthologs containing an acidic 222 residue prefer ADH over PDH activity

The amino acid sequence alignment of TyrAs showed that the Asp222 residue, which is conserved across plant TyrA_a_ (Schenck et al., 2017) was also highly conserved in clade I (Figure 2). On the other hand, most sequences in clade II, including some archaea TyrA, have a non-acidic Gln residue at the corresponding 222 position (Figure 2), similar to legume TyrA_p_ enzymes (Schenck et al., 2017). Homology models of representative TyrA from clade I—*Arabidopsis thaliana* ADH (AtADH2, Plantea; Rippert and Matringe, 2002) and *S. smaragdinae* DSM 11293 (SsTyrA, spirochaetes)—and clade II—*M. harundinacea* (MhTyrA, archaea)—generated using GmPDH1 structure as the template indeed showed that their acidic and non-acidic residues, respectively, correspond to Asp222 in the active site of plant TyrA (Figure S2). These data together suggest that TyrAs from clade I are likely arogenate-specific TyrA_a_ enzymes, whereas more distantly-related microbial TyrAs from clade II are likely prephenate-specific TyrA_p_ enzymes.

**Figure 3:**
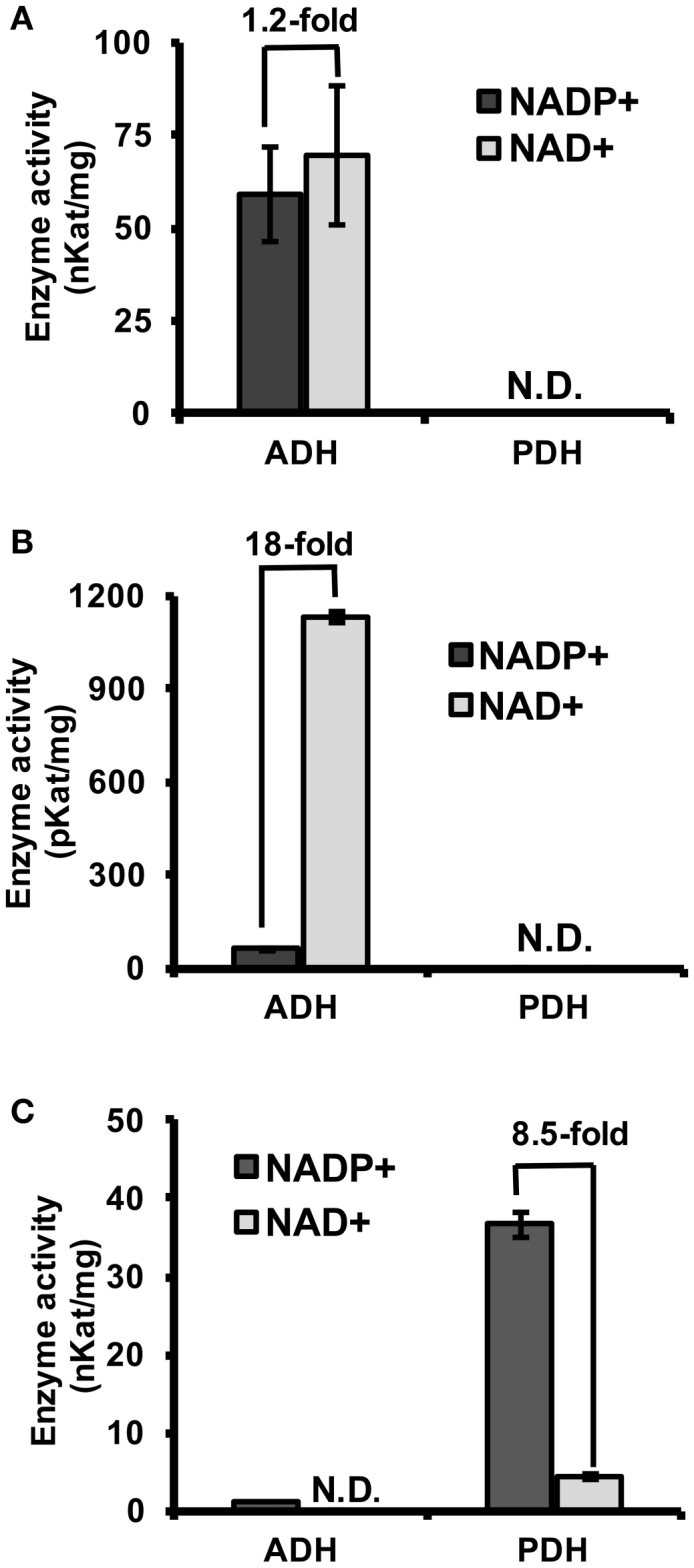
Substrate and cofactor specificity of microbial TyrA orthologs. ADH and PDH assays were performed with 0.8 mM arogenate and prephenate, respectively, and 0.8 mM cofactor (NADP^+^, black; NAD^+^, gray). **(A)** Purified recombinant SsTyrA (spirochaetes) was used to test enzymatic activity, and shown as the average in nKat/mg protein ± SEM of *n* = 3. **(B)** α-proteobacteria TyrA (OiTyrA) cell lysate was used as purification of the recombinant enzyme was not successful. Average enzymatic activity is shown as pKat/mg protein ± SEM of *n* = 3 **(C)** Purified recombinant MhTyrA (archaea) was used to test enzymatic activity, and shown as the average in nKat/mg protein ± SEM of *n* = 3. N.D. no activity detected. Cofactor preference is indicated by the fold-change over the bars.

To experimentally test if TyrAs from clade I have ADH activity, representative TyrA orthologs from two distinct subclades of clade I, spirochaetes (SsTyrA) and α-proteobacteria (*O. intermedium*; OiTyrA, Figure 2, Figure S1), were expressed in *E. coli* as recombinant enzymes and biochemically characterized. SsTyrA and OiTyrA were chosen as they are located at key phylogenetic boundaries within clade I and contain residues required for cofactor binding and catalysis (Figure S2, Data S2). Purified SsTyrA recombinant enzyme showed ADH activity with a slight preference for NAD^+^ over NADP^+^ cofactor; however, PDH activity was not detectable (Figure 3A). Similarly, the *E. coli* cell lysate expressing OiTyrA had ADH but not PDH activity and strongly preferred NAD^+^ over NADP^+^ cofactor (Figure 3B), although the purification of OiTyrA was not successful due to low expression. These results demonstrate that microbial TyrA orthologs from clade I, which contain an acidic residue at the corresponding 222 position (Figure 2, Data S2), are arogenate-specific TyrA_a_ enzymes.

### An archaeon TyrA containing a non-acidic residue prefers PDH over ADH activity

To test if TyrA orthologs from clade II, which contain a non-acidic residue at the corresponding 222 position, are prephenate specific TyrA_p_ enzymes, a representative archaeon TyrA from *M. harundinacea* (MhTyrA) was biochemically characterized. MhTyrA was chosen as no TyrAs from its subclade of clade II have previously been characterized (Figure 2). Also, MhTyrA is a monofunctional enzyme, while some archaea, fungi, and γ-proteobacteria orthologs in clade II are bifunctional and have a chorismate mutase enzyme domain (Hudson et al., 1984; Shlaifer et al., 2017). MhTyrA was expressed in *E. coli* and the recombinant enzyme was purified to homogeneity using affinity-chromatography (Figure S3) and used for biochemical analyses. Unlike plant and microbial TyrA_a_ orthologs from clade I, MhTyrA showed strong PDH and very weak ADH activity (Figure 3C). Interestingly, MhTyrA strongly preferred NADP^+^ over NAD^+^ cofactor (Figure 3C), like plant TyrAs (Gaines et al., 1982; Connelly and Conn, 1986). These results suggest that TyrA orthologs from clade II that have a non-acidic residue at the corresponding 222 position are TyrA_p_ enzymes that strongly prefer prephenate over arogenate substrate.

### A single D208N mutation introduces PDH activity in a spirochaetes TyrA_a_

**Figure 4:**
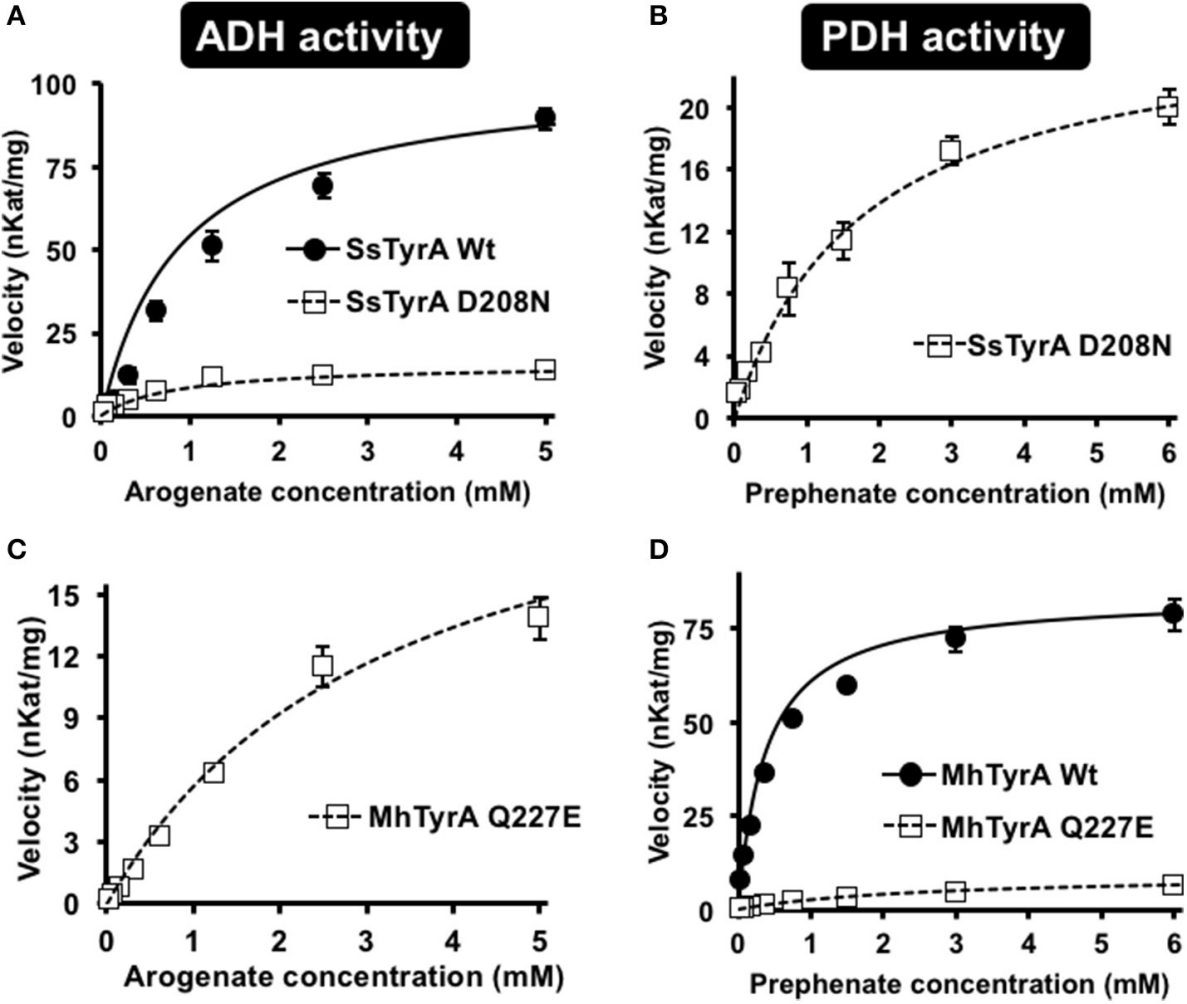
Kinetic analysis of Wt and mutant enzymes of SsTyrA_a_ and MhTyrA_p_. Kinetic analyses were performed with SsTyrA_a_ Wt (filled circle) and D208N mutant (open square) enzymes with arogenate **(A)** and SsTyrA_a_ D208N mutant (open square) with prephenate **(B)**. Kinetic analyses were also performed with MhTyrA_p_ Wt (filled circle) and Q227E mutant (open square) enzymes using arogenate **(C)** and prephenate **(D)**. Initial velocity values at each substrate concentration were fit to the Michaelis-Menten equation using Origin software. Kinetic analyses were conducted for MhTyrA_p_ Wt using 3.41 μg of purified recombinant enzyme, and 4.56 and 2.28 μg of purified recombinant Q227E using prephenate and arogenate, respectively. Kinetic analyses conducted for SsTyrA_a_ Wt used 0.30 μg of purified recombinant enzyme, and 0.39 μg of purified recombinant D208N. Data are means ± SEM (*n* = 3 independent experiments). Error bars smaller than symbols are not shown.

To test if the acidic residue at the corresponding 222 position in spirochaetes SsTyrA_a_ is involved in its substrate specificity, the D208N mutation that converts the active site acidic residue into a non-acidic residue was introduced in SsTyrA. The purified recombinant SsTyrA_a_ D208N enzyme (Figure S3) drastically decreased its original ADH activity (Figure 4A), but now exhibited PDH activity (Figure 4B), with a slight alteration in cofactor specificity in the mutant compared to Wt (Figure S4).

Kinetic analyses showed that SsTyrA_a_ Wt enzyme did not accept prephenate and had a *K*_m_ for arogenate of 901 μM (Table 1), which is substantially higher than previously reported plant and microbial TyrA enzymes (Rippert and Matringe, 2002; Bonvin et al., 2006; Ku et al., 2010; Schenck et al., 2015). The SsTyrA_a_ D208N mutant exhibited a seven-fold lower catalytic efficiency (*k*_*cat*_/*K*_m_) with arogenate than Wt, though the *K*_m_ was not altered (Table 1). SsTyrA_a_ D208N showed significant PDH activity that was absent in Wt, though its *K*_m_ was still much higher than previously reported TyrA enzymes (Rippert and Matringe, 2002; Bonvin et al., 2006; Ku et al., 2010; Schenck et al., 2015) and had poor catalytic efficiency (Table 1). These results suggest that a single mutation of the active site acidic residue to a non-acidic residue can alter the substrate specificity of spirochaetes TyrA, similar to plant TyrAs (Schenck et al., 2017).

**Table 1:** Kinetic analysis of MhTyrA_p_ and SsTyrA_a_ Wt and mutant enzymes.

**Enzyme**	**Substrate**	***k_*cat*_* (s^−1^)**	***K*_m_ (mM)**	***k_*cat*_*/*K*_m_ (mM^−1^ s^−1^)**
SsTyrA_a_ Wt	Arogenate	3.44 ± 0.31	0.901 ± 0.15	4.03 ± 1.01
SsTyrA_a_ Wt	Prephenate	N.D.	N.D.	N.D.
SsTyrA_a_ D208N	Arogenate	0.450 ± 0.01	0.847 ± 0.16	0.568 ± 0.14
SsTyrA_a_ D208N	Prephenate	0.863 ± 0.12	1.742 ± 0.63	0.587 ± 0.40
MhTyrA_p_ Wt	Arogenate	N.D.	N.D.	N.D.
MhTyrA_p_ Wt	Prephenate	2.44 ± 0.38	0.378 ± 0.02	6.44 ± 0.54
MhTyrA_p_ Q227E	Arogenate	0.704 ± 0.03	3.290 ± 0.22	0.213 ± 0.05
MhTyrA_p_ Q227E	Prephenate	0.285 ± 0.02	2.669 ± 0.32	0.107 ± 0.04

*N.D. activity below detection limit, Kinetic parameters are the average of three replicate experiments (n = 3) ± SEM. Kinetic analyses were conducted as described in Figure 4 legend*.

### A single Q227E mutation introduces ADH activity in an archaeon TyrA_p_

To test if the non-acidic residue of MhTyrA_p_ at the corresponding 222 position (Gln227) is involved in prephenate substrate specificity, site-directed mutagenesis was performed on MhTyrA_p_ to replace Gln227 with acidic Glu and generate the MhTyrA_p_ Q227E mutant. The purified recombinant MhTyrA_p_ Q227E enzyme (Figure S3) showed decreased PDH activity (Figure 4D) with a substantial gain of ADH activity (Figure 4C, Table 1) without altering cofactor preference (Figure S4).

Further kinetic analyses showed that Wt MhTyrA_p_ had a *K*_m_ of 378 μM and turnover rate (*k*_*cat*_) of 2.4 s^−1^ using prephenate substrate and NADP^+^ cofactor (Figure 4, Table 1), which are comparable to previously characterized microbial TyrA_p_ enzymes (Bonvin et al., 2006; Ku et al., 2010). The very weak ADH activity of MhTyrA_p_ Wt (Figure 4, Table 1) precluded it from kinetic analysis using arogenate.

The Q227E mutant, on the other hand, exhibited almost 10-fold reduction in *K*_m_ for prephenate (2.7 mM), while the catalytic efficiency (*k*_*cat*_/*K*_m_) was reduced by 60-fold (0.1 vs. 6.4 mM^−1^ s^−1^, Figure 4D, Table 1). The Q227E mutant displayed substantial ADH activity compared to the Wt enzyme with a *K*_m_ for arogenate of 3.3 mM, similar to that of Q227E for prephenate (2.7 mM, Figure 4C, Table 1) though still 10-fold higher than that of the Wt enzyme for prephenate (Figure 4D, Table 1) and other previously characterized TyrA_a_ enzymes (Bonvin et al., 2006; Ku et al., 2010; Schenck et al., 2015, 2017). The Q227E mutant had roughly two-fold higher catalytic efficiency with arogenate than with prephenate (0.2 vs. 0.1 mM^−1^ s^−1^, Figure 3). These results demonstrate that the Q227E mutation can shift the substrate preference of MhTyrA_p_ from prephenate to arogenate, suggesting that the single residue is responsible for substrate specificity of archaea TyrA_p_ enzymes.

## Discussion

Previous studies suggest that microbes predominantly use a PDH-mediated pathway to synthesize Tyr, whereas plants mainly use an ADH-mediated Tyr pathway (Jensen and Pierson, 1975; Bentley, 1990; Siehl, 1999; Rippert and Matringe, 2002; Maeda and Dudareva, 2012; Schenck et al., 2015, 2017). In this study, structure-guided phylogenetic analyses from diverse organisms identified ADH-like sequences in some bacteria, e.g., spirochaetes, α- and δ-proteobacteria, which form a monophyletic clade with plant TyrAs (Figure 2, Figure S1). Biochemical characterization further demonstrated that TyrAs from spirochaetes and α-proteobacteria indeed have ADH, but not PDH activity (Figures 3A,B). A native TyrA enzyme purified from the α-proteobacteria *P. immobile*, which belongs to the same α-proteobacteria genus found in clade I, was also previously shown to have ADH, but not PDH activity (Mayer et al., 1985). Therefore, our study revealed that arogenate-specific TyrA_a_ enzymes are more widely distributed in microbes than previously thought.

Previous evolutionary studies revealed that plant aromatic amino acid pathway enzymes are derived from a wide range of, and sometimes unexpected microbial origins (Richards et al., 2006; Reyes-Prieto and Moustafa, 2012; Dornfeld et al., 2014). For example, plant shikimate kinase is most likely derived from cyanobacteria endosymbiosis (Richards et al., 2006) whereas plant prephenate aminotransferase and arogenate dehydratase involved in Phe biosynthesis are sister to Chlorobi/Bacteroidetes orthologs (Dornfeld et al., 2014). However, the evolutionary origin of plant TyrAs is currently unknown. TyrAs from some spirochaetes were more closely-related to plant and algae TyrA_a_s than other microbial TyrAs from clade I (Figure 2, Figure S1) and, like Plantae TyrA_a_ enzymes, had a conserved acidic residue at the corresponding 222 position. BlastP searches across different spirochaetes genomes showed that plant-like TyrAs are restricted to the order Spirochaetales, and absent in Leptospirales, Brevinematales, and Brachyspirales (Figure S5; Gupta et al., 2013). Thus, the current result suggests that the common ancestor of algae and plants acquired a TyrA_a_ enzyme from a spirochaetes ancestor likely through a novel HGT event, rather than from an α-proteobacteria through mitochondria symbiosis (Gray et al., 1999).

The archaeon MhTyrA from clade II preferred PDH over ADH activity (Figure 3C) and had a non-acidic residue at the 222 position (Figure 2, Figure S1). This is consistent with previously-characterized clade II TyrA enzymes from γ-proteobacteria and fungi, which also preferred PDH over ADH activity (Mannhaupt et al., 1989; Christendat and Turnbull, 1999; Chiu et al., 2010) though they belonged to distinct subclades (Figure 2). As almost all TyrA sequences within clade II have a non-acidic residue (Gln or Asn) at the corresponding 222 position, except for Chloroflexi TyrAs (Figure 2, Figure S1), they are likely prephenate-specific TyrA_p_ enzymes. Previously characterized microbial TyrA_p_ enzymes had similar *K*_m_ with prephenate as MhTyrA_p_ (Figure 4, Table 1); however the catalytic efficiency of MhTyrA_p_ was lower than previously characterized TyrA_p_ enzymes (Bonvin et al., 2006; Ku et al., 2010). This implies that MhTyrA may also have alternative *in vivo* substrates and further genetic studies are needed to determine the *in vivo* function of MhThrA_p_.

In plant TyrAs, an acidic residue at the corresponding 222 position confers ADH activity by directly interacting with the side chain amine of arogenate, and when mutated to a non-acidic Asn, switches to PDH activity (Schenck et al., 2017). Consistently, in OiTyrA_a_ and SsTyrA_a_, which contain acidic residues at the corresponding 222 position prefer ADH activity (Figure 3). Furthermore, mutation of the corresponding acidic residue into a non-acidic residue on SsTyrA_a_ introduced novel PDH activity (Figure 4, Table 1). The reciprocal mutation (Gln to Glu) on MhTyrA_p_ reduced PDH activity while gaining ADH activity (Figure 4, Table 1), further supporting that the corresponding 222 position in microbial TyrA enzymes is also important for their substrate specificity. However, ADH activity of MhTyrA_p_ Q227E was only two-fold higher than its PDH activity and still 30-fold lower than PDH of MhTyrA_p_ Wt. Also, PDH activity of SsTyrA_a_ D208N was seven-fold lower than SsTyrA_a_ Wt with arogenate (Figure 4, Table 1). These results suggest that residues besides the corresponding 222 substrate specificity determining residue likely contribute to overall catalytic activity of microbial TyrA enzymes. These data together suggest that mutation of the non-acidic to an acidic residue at the corresponding 222 position played a key role in the evolution of arogenate-specific TyrA_a_ enzymes in microbes from clade I that gave rise to plant TyrAs.

The outgroup (clade III) appears to contain TyrA enzymes with both PDH and ADH activity (Xia and Jensen, 1990; Zhao et al., 1993; Bonvin et al., 2006; Legrand et al., 2006). Homology models of microbial TyrAs from the outgroup (e.g., *Bifidobacterium dentium* TyrA; BdTyrA) were compared to previously crystallized GmPDH1 (Schenck et al., 2017) and Synechocystis ADH (Legrand et al., 2006) to determine if the substrate specificity mechanism of TyrAs from clade I and II are also conserved in clade III TyrAs (Figure S6). The global conformations of these divergent TyrA proteins from clade I and III are similar in structure, though there are some differences, such as additional α-helices around the C-terminal dimerization domain (Figure S6). All structures have conserved catalytic Ser101 and His124 (Christendat and Turnbull, 1999; Sun et al., 2006) that directly interact with ring hydroxyl of arogenate and prephenate substrate (Schenck et al., 2017), suggesting that the key catalytic residues have been maintained across divergent TyrAs. However, the two loop regions surrounding and recognizing the substrate side chain by the 222 residue (Schenck et al., 2017), are not well conserved in clade III as compared to clade I TyrAs (Figure S6). This makes it difficult to confidently assign a corresponding residue in clade III TyrAs to the 222 position of clade I TyrAs (Figure 2, Data S2). Thus, clade III TyrAs likely use a different molecular mechanism(s) for their substrate specificity than plant and closely-related microbial TyrAs from clade I and II.

Alteration of substrate specificity of microbial TyrA enzymes provides another example of a single active site residue that modifies substrate specificity of plant and microbial enzymes (Louie et al., 2006; He et al., 2011; Fan et al., 2016). Substitutions between active site Val and Phe residues switch the acyl-CoA substrate specificity of *Solanum* acylsugar acyltransferases that belong to the BAHD acyltransferase family (Fan et al., 2016). A single mutation of the conserved active site Leu residue of isopropylmalate dehydrogenases involved in leucine biosynthesis is sufficient to convert their specificity to 3-(2′-methylthio)ethylmalate, an intermediate of the methionine chain-elongation pathway required for aliphatic glucosinolate biosynthesis (He et al., 2011). Mutating His89 of a microbial Tyr ammonia lyase (TAL) into Phe also switches its substrate specificity to prefer Phe instead of Tyr (Louie et al., 2006). Many of these examples are the result of gene duplications followed by neofunctionalization by a single key amino acid mutation, resulting in a novel substrate specificity and recruitment to a different metabolic pathway (Leong and Last, 2017). Despite the single amino acid mutation that switches substrate specificity of TyrA dehydrogenases, they are still involved in the same Tyr biosynthesis but alters the pathway architecture.

In conclusion, the current study revealed that arogenate-specific TyrA_a_ enzymes evolved in some bacterial lineages, through the acquisition of an acidic residue at the 222 position, which later gave rise to the TyrAs of algae and land plants likely through a novel HGT event. More recently, the same residue was mutated back to a non-acidic residue uniquely in legume plants, which resulted in prephenate-specific TyrA_p_ enzymes (Schenck et al., 2017). Thus, in the course of TyrA enzyme evolution, microbial TyrA_p_ were converted into microbial TyrA_a_ and then to legume-specific TyrA_p_ by altering the same active site residue from a non-acidic to an acidic, and then back to a non-acidic residue. Previous studies proposed that the ubiquitous presence of the ADH-mediated Tyr pathway among photosynthetic organisms is to avoid futile cycling of tocopherol and plastoquinone biosynthesis from HPP (Siehl, 1999; Graindorge et al., 2014). Identification of arogenate-specific TyrA among many non-photosynthetic microbes may require revisiting the biological significance of the ADH vs. PDH-mediated Tyr biosynthetic pathways in diverse organisms. Given that arogenate and prephenate substrate specificity of TyrAs can be readily converted by a single residue (Figure 4, Table 1; Schenck et al., 2017), there must be significant selection pressure to maintain the acidic 222 residue and thus ADH activity in many organisms. The molecular mechanism and the key amino acid residue regulating the biochemical properties of diverse TyrAs also enables the optimization of Tyr biosynthesis via two alternative Tyr biosynthetic pathways in both plants and microbes, for enhanced production of pharmaceutically important natural products derived from Tyr (e.g., morphine and vitamin E).

## Author contributions

CAS and YM performed experiments and analyzed data; CAS and HAM conceived the experiments, and CAS wrote the manuscript. All authors read and edited the manuscript.

### Conflict of interest statement

The authors declare that the research was conducted in the absence of any commercial or financial relationships that could be construed as a potential conflict of interest.
