# Supplementary material for: Conserved Molecular Mechanism of TyrA Dehydrogenase Substrate Specificity Underlying Alternative Tyrosine Biosynthetic Pathways in Plants and Microbes
Source: Front Mol Biosci. 2017 Nov 7;4:73. doi: 10.3389/fmolb.2017.00073 (PMC5681985; doi:10.3389/fmolb.2017.00073)
Supplement: Supplementary file 2 [file DataSheet2.pdf]

```
s003              1 ----------------------------------------------------------------------
S.spPCC6803_Tyr   1 ----------------------------------------------------------------------
B.animalis_TyrA   1 ----------------------------------------------------------------------
B.pseudocatenul   1 ----------------------------------------------------------------------
B.adolescentis_   1 ----------------------------------------------------------------------
B.dentium_TyrA    1 ----------------------------------------------------------------------
S.meliloti_TyrA   1 ----------------------------------------------------------------------
R.leguminosarum   1 ----------------------------------------------------------------------
T.sp_TyrA         1 ----------------------------------------------------------------------
D.invisus_TyrA    1 ----------------------------------------------------------------------
B.anthracis_Tyr   1 ----------------------------------------------------------------------
S.thermophilus_   1 ----------------------------------------------------------------------
A.aeolicus_TyrA   1 ----------------------------------------------------------------------
R.pneumotropicu   1 --------------MEALKDLRSEIDSLDREVIQLFSKRLKLVSQVGKVKHQHGLPIYAPDREIAMLQAR
s001              1 ----------------------------------------------------------------------
H.influenzae_Ty   1 -----------MSFMEALKDLRSEIDSLDRELIQLFAKRLELVSQVGKVKHKHGLPIYAPEREIAMLKAR
A.succinogenes_   1 --------------MEALKEIRKEIDALDKELIEVFAKRLALVKKVGEIKHKHGLPIYVPEREADMLAAR
H.massiliensis_   1 --------------MDALKELRAEIDHLDRELLQLFAKRLALVKKVGEIKHRCGLPIYAPEREADMLAAR
H.somni_TyrA      1 --------------MDVLKNLRTEIDCLDRELLHLFSQRLELVRKVGKVKHQYGLPIYVPERENEMLQAR
A.actinomycetem   1 --------------MERLAELREQIDSLDHELLQLLAKRQQVVHKVGEVKQQHGLPIYAPQREAEMLQRK
A.aphrophilus_T   1 --------------MERLAELRAQIDSLDEELLQLLAKRLDVVHKIGEVKQQHGLPIYVPQRESEMLQKK
N.archeti_TyrA    1 -------------MVAELTALRDQIDEVDKALLDLLATRLKLVAEVGEVKSRYGLPIYVPEREASMLASR
Y.kristensenii_   1 -------------MVAELTALRDQIDEVDKALLDLLAKRLHLVAEVGEVKSRYGLPIYVPDREAAMLASR
S.marcescens_Ty   1 -------------MVAKLTALRDQIDEVDKALLDLLAKRLHLVAEVGEVKSRHGLPVYVPEREAAMLASR
E.coli_TyrA       1 -------------MVAELTALRDQIDEVDKALLNLLAKRLELVAEVGEVKSRFGLPIYVPEREASMLASR
C.youngae_TyrA    1 -------------MVAELTALRDQIDEVDKALLDLLAKRLELVAEVGEVKSRFGLPIYVPEREASMLASR
S.boydii_TyrA     1 -------------MVAELTALRDQIDEVDKALLNLLAKRLELVAEVGEVKSRFGLPIYVPEREASMLASR
C.freundii_TyrA   1 -------------MVAELTALRDQIDEVDKVLLDLLARRLELVAEVGEVKSRFGLPIYVPEREASMLASR
E.fergusonii_Ty   1 -------------MVAELTALRDQIDEVDKALLNLLAKRLELVAEVGEVKSRFGLPIYVPEREASMLASR
S.enterica_TyrA   1 -------------MVAELTALRDQIDDVDKALLNLLAKRLELVAKVGEVKSRFGLPIYVPEREASMLASR
C.sakazakii_Tyr   1 -------------MVAELTALRDQIDEVDKALLGLLARRLELVAEVGEVKSRYGLPIYVPEREASMLASR
X.hominickii_Ty   1 -------------MVAELSQLREQIDEVDKALLALLAKRMNLVAQVGEVKSQLGLPIYAPDREATMLASR
P.asymbiotica_T   1 --------------MVVELSQLRDQIDEVDKALLGLLAKRLELVAEIGEVKSRNGLPIYAPDREMAMLAA
M.fervens_TyrA    1 ----------------------------------------------------------------------
M.maripaludis_T   1 ----------------------------------------------------------------------
M.jannaschii_Ty   1 ----------------------------------------------------------------------
M.vulcani_TyrA    1 ----------------------------------------------------------------------
M.burtonii_TyrA   1 ----------------------------------------------------------------------
M.thermophila_T   1 ----------------------------------------------------------------------
M.concilii_TyrA   1 ----------------------------------------------------------------------
M.harundinacea_   1 ----------------------------------------------------------------------
M.barkeri_TyrA1   1 ----------------------------------------------------------------------
M.barkeri_TyrA2   1 ----------------------------------------------------------------------
M.horonobensis_   1 ----------------------------------------------------------------------
M.mazei_TyrA      1 ----------------------------------------------------------------------
C.methanoperede   1 ----------------------------------------------------------------------
P.homiensis_Tyr   1 ----------------------------------------------------------------------
R.pomeroyi_TyrA   1 ----------------------------------------------------------------------
M.hallyeonensi_   1 ----------------------------------------------------------------------
J.pohangensis_T   1 ----------------------------------------------------------------------
R.halocynthiae_   1 ----------------------------------------------------------------------
P.ascidiaceicol   1 ----------------------------------------------------------------------
O.pituitosum_Ty   1 ----------------------------------------------------------------------
O.anthropi_TyrA   1 ----------------------------------------------------------------------
O.intermedium_T   1 ----------------------------------------------------------------------
B.canariense_Ty   1 ----------------------------------------------------------------------
P.inhibens_TyrA   1 ----------------------------------------------------------------------
P.zucineum_TyrA   1 ----------------------------------------------------------------------
S.spMCT13_TyrA    1 ----------------------------------------------------------------------
A.excentricus_T   1 ----------------------------------------------------------------------
S.cellulosum_Ty   1 ----------------------------------------------------------------------
M.xanthus_TyrA    1 ----------------------------------------------------------------------
C.bacteriumRBG_   1 ----------------------------------------------------------------------
D.mccartyi_TyrA   1 ----------------------------------------------------------------------
D.mccartyi_TyrA   1 ----------------------------------------------------------------------
D.spWBC-2_TyrA    1 ----------------------------------------------------------------------
D.lykanthropore   1 ----------------------------------------------------------------------
D.alkenigignens   1 ----------------------------------------------------------------------
A.sulfaticallid   1 ----------------------------------------------------------------------
A.profundus_Tyr   1 ----------------------------------------------------------------------
A.veneficus_Tyr   1 ----------------------------------------------------------------------
G.ahangari_TyrA   1 ----------------------------------------------------------------------
F.placidus_TyrA   1 ----------------------------------------------------------------------
```

```
B.spBAL6_X_TyrA    1 ----------------------------------------------------------------------
B.spMedPE-Swde_    1 ----------------------------------------------------------------------
H.marinus_TyrA     1 ----------------------------------------------------------------------
B.bacterium_Tyr    1 ----------------------------------------------------------------------
D.bacterium4572    1 ----------------------------------------------------------------------
D.multivorans_T    1 ----------------------------------------------------------------------
D.bacterium_Tyr    1 ----------------------------------------------------------------------
D.cetonica_TyrA    1 ----------------------------------------------------------------------
D.tepidiphila_T    1 ----------------------------------------------------------------------
S.africana_TyrA    1 ----------------------------------------------------------------------
S.bajacaliforni    1 ----------------------------------------------------------------------
S.smaragdinae_T    1 ----------------------------------------------------------------------
S.bacterium_Tyr    1 ----------------------------------------------------------------------
S.bacteriumRBG_    1 ----------------------------------------------------------------------
S.odontotermiti    1 ----------------------------------------------------------------------
S.bacterium4572    1 ----------------------------------------------------------------------
C.Bathyarchaeot    1 ----------------------------------------------------------------------
M.truncatula_3g    1 ----------------------------------------------------------------------
s002               1 ----------------------------------------------------------------------
G.max_11g233900    1 ----------------------------------------------------------------------
G.max_18g023100    1 ----------------------------------------------------------------------
B.bituminosa_20    1 ----------------------------------------------------------------------
P.vulgaris_1g24    1 ----------------------------------------------------------------------
L.sativus_20036    1 ----------------------------------------------------------------------
A.americana_205    1 ----------------------------------------------------------------------
M.truncatula_5g    1 ----------------------------------------------------------------------
G.max_14g055300    1 ----------------------------------------------------------------------
C.cajan_05579      1 ----------------------------------------------------------------------
P.trichocarpa_8    1 ----------------------------------------------------------------------
C.clementina_32    1 ----------------------------------------------------------------------
S.lycopersicum0    1 ----------------------------------------------------------------------
A.trichopoda_00    1 ----------------------------------------------------------------------
S.fallax_TyrA      1 MDRNCCEAIANTLQRKLSLMARTQGTWHGICFLPGGSASNSLAHSSSTAVTICGMTCCTKNTLQQLSWKL
S.lycopersicum0    1 ------------------------------------------MLSFTPLQSKPTPTSNSNRFSNLTNPTS
A.trichopoda_00    1 ----------------------------------------------------------------------
P.patens_TyrA2     1 ------------MVAVGVMGALPLTSIAHCATTSSYQQTRYGVVGCKVPALVQIDSVFGFAASELQGDFR
P.patens_TyrA1     1 ---------------------MNGLIPTPVAHTTMIFSHQQTKHVGSSKLPILRQNESSGAVELQGDFRK
A.thaliana_1g15    1 ---------------------------------------------------------------MLLHFSP
S.lycopersicum0    1 ------------------MFSLSSIQSNNIQSQSSSSLLFNHHHQHSTISTRFHHHRLLFPLRAQNSDLT
B.distachyon_1g    1 ---------------------------------------MSSSGRF-HQPPSCRRPAALAQSLRASTTTV
S.italica_4g275    1 --------------------------------------------------MAASSSVRLHHQPSPSLRLP
S.italica_4g174    1 ----------------------------------MASSLRHFAGPSCFTATAAASSGASGFLRRYAPNFC
B.distachyon_1g    1 -------------------------------------------MASSLVHLSSPAGGRTAAAPPSLLSRF
A.trichopoda_00    1 ----------------------------------------------------------------------
A.trichopoda_00    1 -------------------------------------------MAAFQFCPCAQQVHQPKLLLPPSLAPT
C.reinhardtii_0    1 -----------------------------------MLRARPLAPSVPTCTSSSCSTSGASLVPILAAAEC
C.subellipsoide    1 ----------------------------------------------------------------------
O.lucimarinus_T    1 -----------------------------------------------------------------MIVRC
C.merolae_TyrA     1 ----------------------------------------------------------------------
G.sulphuraria_T    1 ----------------------------------------------------------------------
S.cryophilus_Ty    1 ----------------------------------------------------------------------
P.decumbens_Tyr    1 ----------------------------------------------------------------------
A.carbonarius_T    1 ----------------------------------------------------------------------
S.complicata_Ty    1 ----------------------------------------------------------------------
S.japonicus_Tyr    1 ----------------------------------------------------------------------
S.octosporus_Ty    1 ----------------------------------------------------------------------
S.pombe_TyrA       1 ----------------------------------------------------------------------
```

```
s003                1 --------------------------------------------------------------MK-----I
S.spPCC6803_Tyr     1 --------------------------------------------------------------MK-----I
B.animalis_TyrA     1 ---------------------MHEE----TIKQ-WDRD------------NAG-EPQIAPEGIECP-KHV
B.pseudocatenul     1 -----------------------------------------------------------MVKQR-----I
B.adolescentis_     1 -----------------------------------------------------------MVKPR-----I
B.dentium_TyrA      1 -----------------------------------------------------------MVKQR-----I
S.meliloti_TyrA     1 ---------------------------------------------------------MAQQFQT-----I
R.leguminosarum     1 ---------------------------------------------------------MNVQFDR-----I
T.sp_TyrA           1 ------------------------------------------------------------MIRK-----A
D.invisus_TyrA      1 ---------------------------------------------------------MDFSKTT-----A
B.anthracis_Tyr     1 -------------------MKIKKE----SLE-------------------------MRQMRKK-----V
S.thermophilus_     1 -----------------------------------------------------------MSKKT-----I
A.aeolicus_TyrA     1 -------------------------------------------------------------MQN-----V
R.pneumotropicu    57 RLEAEKAGISPDLIEDVLRRFMRES----YTNE-NQF---------------GFKTVNPNIQKI-----V
s001                1 --------------------------------------------------GFK-TIN-SDIHKI-----V
H.influenzae_Ty    60 RLEAEKAGISADLIEDVLRRFMRES----YANE-NQF---------------GFKTINPDIHKI-----V
A.succinogenes_    57 RQEAEKTGVPADLIEDVLRRLMRES----YASE-NDY---------------GFKTVNPAIKKI-----V
H.massiliensis_    57 RQEAENMGVPADLIEDVLRRLMRES----YSRE-NQF---------------GFKTVNPAIRKI-----V
H.somni_TyrA       57 RNEAEKLGVSPDLIEDVLRRLMRES----YTRE-NQY---------------GFKTANPEIKKI-----V
A.actinomycetem    57 REAAEQMGLSPNLIEDVLRRIMRES----YVSE-NQF---------------GFKTVNPQIRKI-----V
A.aphrophilus_T    57 REAATKLGLSPNLIEDILRRIMRES----YVSE-NQF---------------GFKTVNPQIRKI-----V
N.archeti_TyrA     58 RKEAQALGVPPDLIEDVLRRVMRES----YSSE-NDK---------------GFKTLCPQLRPI-----V
Y.kristensenii_    58 RQEAEALGVPPDLIEDVLRRVMRES----YTSE-NDK---------------GFKTLCPQLRPV-----V
S.marcescens_Ty    58 RQEAENLGVPPDLIEDVLRRVMRES----YVSE-NDK---------------GFKTLCPQLRPI-----V
E.coli_TyrA        58 RAEAEALGVPPDLIEDVLRRVMRES----YSSE-NDK---------------GFKTLCPSLRPV-----V
C.youngae_TyrA     58 RAEAEALGVPPDLIEDVLRRVMRES----YSSE-NDK---------------GFKTLCPSLRPV-----V
S.boydii_TyrA      58 RAEAEALGVPPDLIEDVLRRVMRES----YSSE-NDK---------------GFKTLCPSLRPV-----V
C.freundii_TyrA    58 RAEAEALGVPPDLIEDVLRRVMRES----YSSE-NDK---------------GFKTLCPSLRPV-----V
E.fergusonii_Ty    58 RAEAEALGVPPDLIEDVLRRVMRES----YSSE-NDK---------------GFKTLCPALRPV-----V
S.enterica_TyrA    58 RAEAEAIGVPPDLIEDVLRRVMRES----YSSE-NDK---------------GFKTLCPSLRPV-----V
C.sakazakii_Tyr    58 RHEAETLGVPPDLIEDVLRRVMRES----YSSE-NDK---------------GFKTLNPALRPV-----V
X.hominickii_Ty    58 RQEAENMGIPPDLIEDILRRIMRES----YVSE-NNK---------------GFKKFRSHLGPV-----V
P.asymbiotica_T    57 RRQEAEEFGVPPDLIEDILRRIMRE---SYTSE-NNK---------------GFKPLCPQLGPI-----V
M.fervens_TyrA      1 ---------------------MKN-------------------------------------KLT-----I
M.maripaludis_T     1 ---------------------MEK-------------------------------------IMI-----I
M.jannaschii_Ty     1 ---------------------MKNT------------------------------------NLT-----I
M.vulcani_TyrA      1 -------------------------------------------------------------MKV-----L
M.burtonii_TyrA     1 -------------------------------------------------------------MKM-----L
M.thermophila_T     1 ---------------------------------------------------------------M-----L
M.concilii_TyrA     1 ------------------------------------------------------------MVRI-----L
M.harundinacea_     1 ------------------------------------------------------MTTMANSGTKN--RML
M.barkeri_TyrA1     1 ---------------------MSAN----LKPG-SQV---------------NTDQK-PEKTKV-----L
M.barkeri_TyrA2     1 ---------------------MSAN----LKPG-SQV---------------NTAQN-PEKTKV-----L
M.horonobensis_     1 ---------------------MNYE----TEQIKAEDHYHPTYS-TRSHPEIRRIPDPEKKNSEKT-KVL
M.mazei_TyrA        1 -------------------------------------------------MNTD---YDAGKTKV-----L
C.methanoperede     1 -------------------------------------------------------------MKI-----L
P.homiensis_Tyr     1 ------------------------------------------------------------MSLS-----V
R.pomeroyi_TyrA     1 ---------------------MTTR----PGIA-APS---------------EPVMSCHLTFRS-----V
M.hallyeonensi_     1 ---------------------MNN--------------------------------NNNREPSS-----L
J.pohangensis_T     1 ---------------------MNS--------------------------------TSRNQRTS-----L
R.halocynthiae_     1 ---------------------MSN--------------------------------NCGSQITS-----L
P.ascidiaceicol     1 ---------------------MNN--------------------------------ISKNQNTS-----L
O.pituitosum_Ty     1 ---------------------MSF--------------------------------DENHQHKT-----I
O.anthropi_TyrA     1 ---------------------MSR--------------------------------DNN-QHMK-----I
O.intermedium_T     1 ----------------MRPLRGQSM-----------------------------SRDRN-QHTK-----I
B.canariense_Ty     1 ---------------------MSND-------------------------------SNINSRPL-----I
P.inhibens_TyrA     1 -----------------------------------------------------MPQ--PPQFSR-----I
P.zucineum_TyrA     1 -------------------------------------------------------------MKT-----L
S.spMCT13_TyrA      1 -------------------------------------------------------------MKR-----F
A.excentricus_T     1 ---------------------MLTA----LPFK-RPE---------------LKAAQTVKQVHK-----L
S.cellulosum_Ty     1 ---------------------MPHS----LPHS-GGG----------------LAP--PARIGK-----V
M.xanthus_TyrA      1 ------------------------------------------------------------MTES-----I
C.bacteriumRBG_     1 --------------------------------------------------------------MK-----L
D.mccartyi_TyrA     1 --------------------------------------------------------------MK-----I
D.mccartyi_TyrA     1 --------------------------------------------------------------MK-----I
D.spWBC-2_TyrA      1 ------------------------------------------------------------M-KI-----V
D.lykanthropore     1 ------------------------------------------------------------MKSI-----A
D.alkenigignens     1 -------------------------------------------------------MVDIGPKNI-----A
A.sulfaticallid     1 ---------------------MNR----------------------------------IEQKPK-----I
A.profundus_Tyr     1 --------------------------------------------------------------MR-----I
A.veneficus_Tyr     1 ------------------------------------------------------------MKPG-----I
G.ahangari_TyrA     1 --------------------------------------------------------------MR-----I
F.placidus_TyrA     1 --------------------------------------------------------------MK-----I
```

```
B.spBAL6_X_TyrA     1 --------------------------------------------------------------MK-----V
B.spMedPE-Swde_     1 --------------------------------------------------------------MK-----V
H.marinus_TyrA      1 --------------------------------------------------------------MK-----V
B.bacterium_Tyr     1 --------------------------------------------------------------MN-----V
D.bacterium4572     1 ---------------------------------------------------------------M-----I
D.multivorans_T     1 ---------------------------------------------------------------M-----I
D.bacterium_Tyr     1 ----------------------------------------------------MSETSKGKKNIK-----I
D.cetonica_TyrA     1 -------------------------------------------------------------MSS-----I
D.tepidiphila_T     1 ---------------------------------------------------------------M-----I
S.africana_TyrA     1 --------------------------------------------------------------MQ-----I
S.bajacaliforni     1 ---------------------MPGM-----------------------------------VEHT-----V
S.smaragdinae_T     1 ------------------------M-----------------------------------VEHT-----V
S.bacterium_Tyr     1 --------------------------------------------------------------MR-----I
S.bacteriumRBG_     1 --------------------------------------------------------------MR-----V
S.odontotermiti     1 --------------------------------------------------------------ME-----I
S.bacterium4572     1 -----------------------------------------------------------MFFMR-----I
C.Bathyarchaeot     1 --------------------------------------------------------------MK-----V
M.truncatula_3g     1 -------------------M---S---------------------------------SSSKSLK-----I
s002                1 ------------------------------------------------------------QSLK-----I
G.max_11g233900     1 -------------------MTTMST-------------------------------SSSSQSLK-----I
G.max_18g023100     1 ----------------------MST-------------------------------SSSSQSLK-----I
B.bituminosa_20     1 -------------------FSTMST-------------------------------SSSFQNLK-----I
P.vulgaris_1g24     1 -------------------MSSSS---------------------------------SSSQSLK-----I
L.sativus_20036     1 ---------------------------------------------------------SSSKSLK-----I
A.americana_205     1 ---------------------------------------------------------ASSQSLK-----I
M.truncatula_5g     1 --------------------------------------------------------MSNSPSLK-----I
G.max_14g055300     1 ---------------------------------------------------------MSTWSLK-----I
C.cajan_05579       1 -------------------------------------------------------------SLK-----I
P.trichocarpa_8     1 --------------MAVSSSSSSSS----SSSS-----------------------SSPSRNLK-----I
C.clementina_32     1 -------------------MAVSSP--------------------------------SSSSTLK-----I
S.lycopersicum0     1 -------------------MSSSSS--------------------------------CQPKTLR-----I
A.trichopoda_00     1 -----------------------MPRIRALDAA-QPF---------------DYEAKIMEMHEKISKLKI
S.fallax_TyrA      71 SSRSLCQDVVLHTKVRVPRKRNSSLEVAAIDAA-QSF---------------DYESLRMEELQSGNKLKI
S.lycopersicum0    29 STSRRRHFSVSSPSQVSHHHGRRRLSIKAIDAA-QPY---------------DYEALVSNQYAQSGRLKI
A.trichopoda_00     1 --MSAFSLPATFRVKQRASKLFVMPRIRALDAA-QPF---------------DYEARIMEAHEKSSKLKI
P.patens_TyrA2     59 KPSQLFLGNGFRVSGRQSQWQRSALDIRAVDAA-QPF---------------DYESRKLQELEKSSKLKV
P.patens_TyrA1     50 QPGQLFLRNGSSIERRGRSKHRSRVNVSAIDAA-QPF---------------DFESMRSQELEKSTPLKV
A.thaliana_1g15     8 AKPLISPPNLRRNSPTFLISPPRSLRIRAIDAA-QIF---------------DYETQLKSEYRKSSALKI
S.lycopersicum0    53 TATTNNNYVDLDDNLTRLDKFSKSL----SISN-IEE----------------NTSLNPLLCSNNK-LKI
B.distachyon_1g    31 TCRW---RQQYHHPLAAAPLRLRAVPVRATDAA-RQPFDHL-------SGAVK-----SEEGTHPR-LKI
S.italica_4g275    21 APTNHQFLAARCRWRGHPGGAAAPPLRLRATGAAQPFDSSES-----PPRAVELEKREDQQQQQPR-LKI
S.italica_4g174    37 AFAALRPIRPAAAAAFSAAAGAANPCAPPAAEH-EQHQLRH-------DSDQPTPSPAPAAPAPAA-LRV
B.distachyon_1g    28 SPNFCAFATLRPGPTRPTAATPKHARARTCAEQEQKQEVAAPCRDAYEKPAVWNTTAADSAEAAPP-LRV
A.trichopoda_00     1 ----------------------------------------------------------------------
A.trichopoda_00    28 RASTKIPMFTPHNNLLSAKFSGKPIPAKFNGSH-LKSQI-----------RAC-ANPDSKGSKEEG-LRI
C.reinhardtii_0    36 STRTIAGAARSVPCRRVLGRRARGVRVLAIDAA-QPF---------------DFEHRMKQRISDKEKLKV
C.subellipsoide     1 --------------------MKVRA----LDAP-MPF---------------DFEHKATRILAKRKQLTI
O.lucimarinus_T     6 HGPPASRVFSRLGRIRTARTRTALKGVRALDAA-QPF---------------DGENKQRMREHKIK-LKI
C.merolae_TyrA      1 -----------------------------------------------------MDQVNSSNSLT-----I
G.sulphuraria_T     1 -------------------------------------------------------MNSSTRILK-----I
S.cryophilus_Ty     1 ---------------------MDIC----FDE--------------------------IKKEFQ-----I
P.decumbens_Tyr     1 -----------------------------MGR--------------------------TKEDAS-----I
A.carbonarius_T     1 -----------------------------MGF--------------------------TKETAS-----I
S.complicata_Ty     1 -------------------MATTRM----NGDS-----------------------SSWKETLE-----V
S.japonicus_Tyr     1 -------------------MAVYDA----SQLE------------------------KMKETFT-----I
S.octosporus_Ty     1 ---------------------MECS----VDE--------------------------IKKEFQ-----I
S.pombe_TyrA        1 ----------------------------------------------------------MKETFQ-----V
```

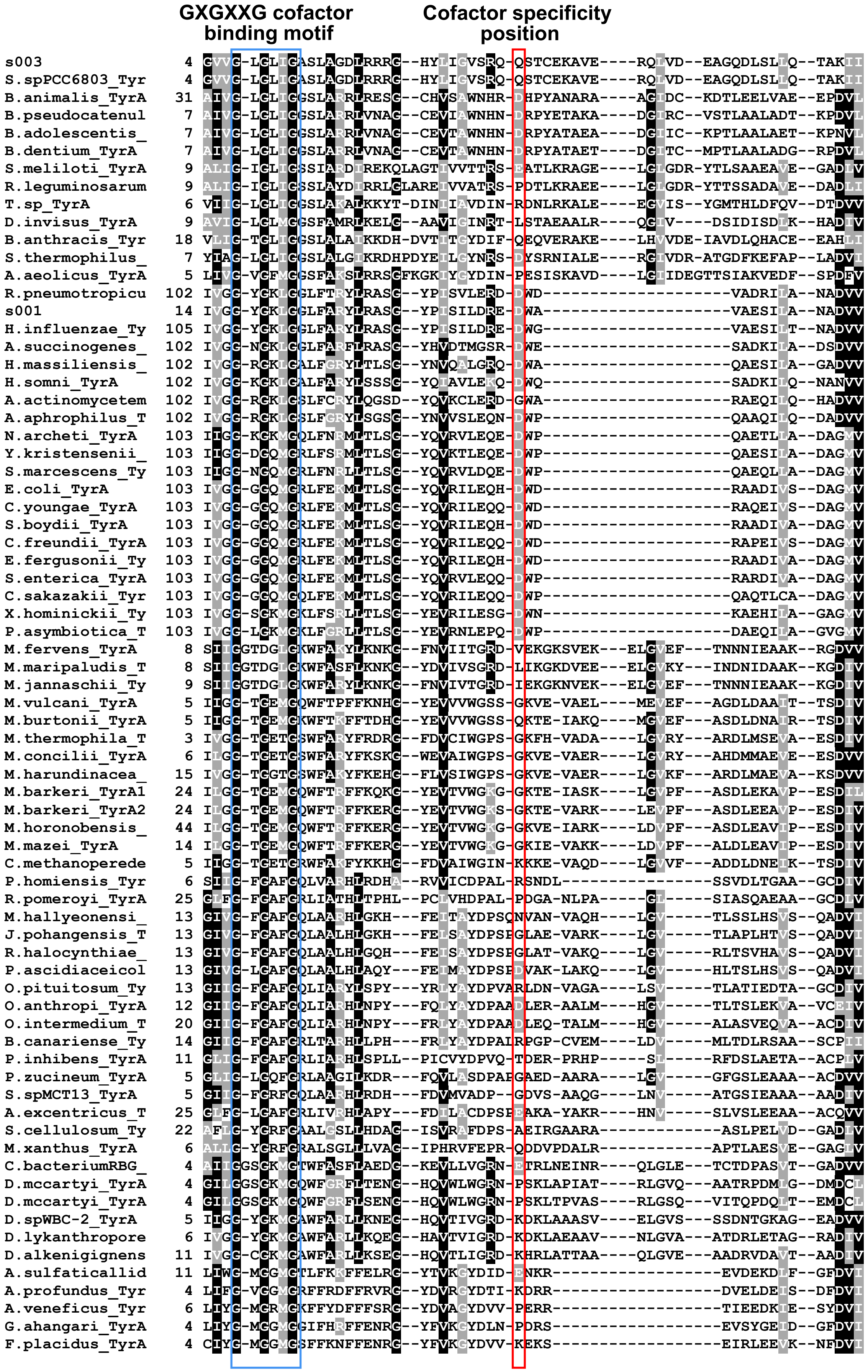
GXGXXG cofactor binding motif
Cofactor specificity position

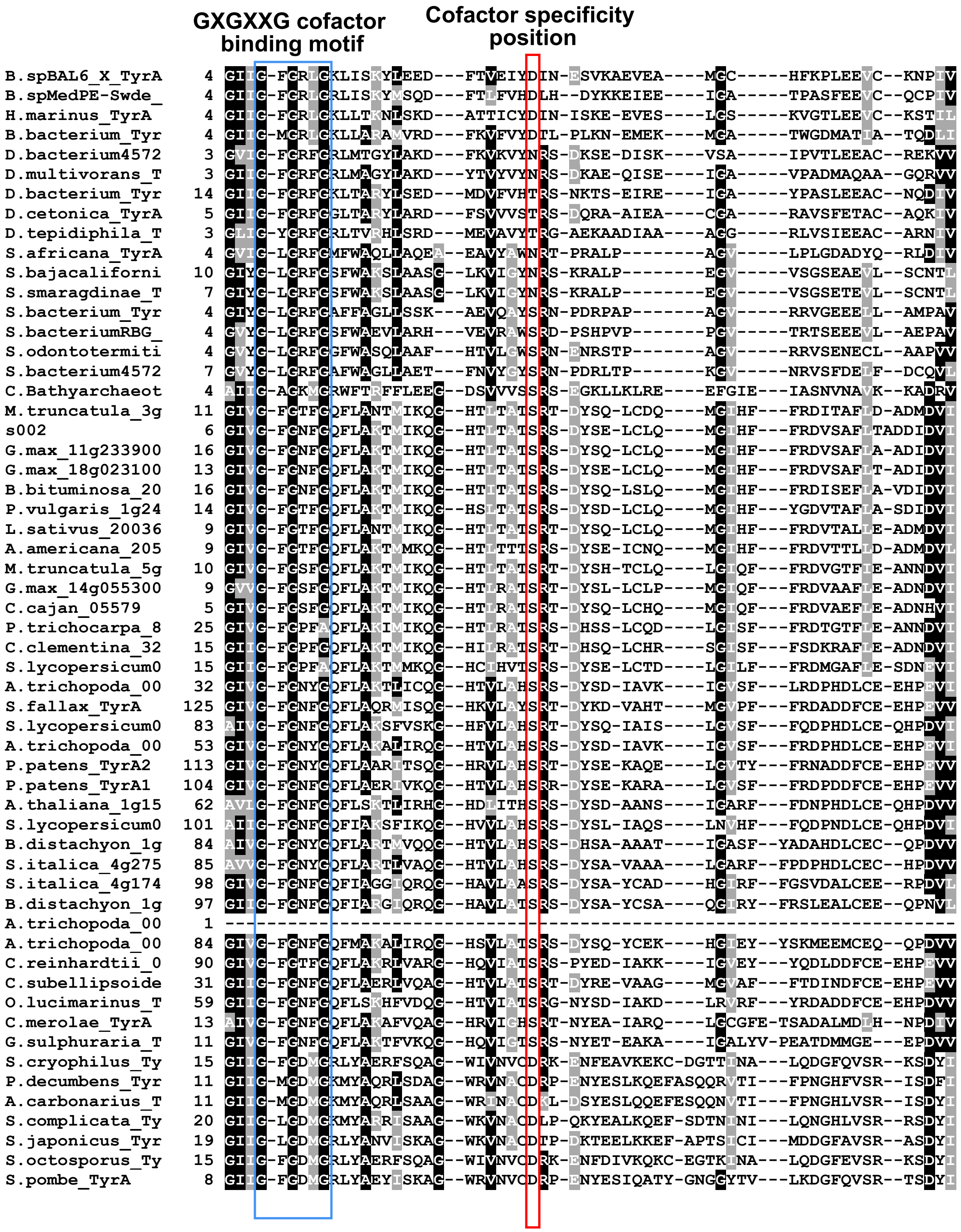
GXGXXG cofactor binding motif
Cofactor specificity position

```
s003                62 FLC-TPIQLI-LPTLEKLIPH---LSPT-AIVTDVASVKT-AIAEPASQLW----SGFIGGHPXAGTAAQ
S.spPCC6803_Tyr     62 FLC-TPIQLI-LPTLEKLIPH---LSPT-AIVTDVASVKT-AIAEPASQLW----SGFIGGHPMAGTAAQ
B.animalis_TyrA     89 MLC-NPLVAM-PQVLQTIKPI---INPQKTTLSDVGSVKG-PVRTQVSEVGLL--DCYVGAHPMAGNEHS
B.pseudocatenul     65 VLC-NPLKAM-PQILGALKPL---IDTDITTLTDVGSVKA-MVREQVKAVGLE--HCYVGAHPMAGNELS
B.adolescentis_     65 VLC-NPLKAM-PEILGALKPL---IDTETTTLTDVGSVKG-MVRDQVKAIGLD--GCYVGAHPMAGNELS
B.dentium_TyrA      65 VLC-NPLKAM-PQILSALKPL---IDQNVTTLTDVGSVKG-MVRDQVKAVGLE--NCYVGAHPMAGNELS
S.meliloti_TyrA     70 VVS-VPVGAS-GAVAAEIAAH---LKPG-AIVTDVGSTKG-SVIAQMAPHLPKD-VHFVPGHPIAGTEHS
R.leguminosarum     70 IVS-VPVGAS-ESVAKEIAGR---LKPG-TIVTDVGSTKA-SVIAQMLPHMPDY-VHFIPGHPIAGTEKS
T.sp_TyrA           65 FIC-TPVGKV-VESVKNIIPY---LKRG-CIVTDVGSTKK-VIMEEVQKFLPDE-IFFIGGHPMAGTEKA
D.invisus_TyrA      65 IFC-TPAKAT-LAFVKNHLPD---FRSD-AIMTDIAGVKG-NLADDIRQILPPG-IDFISSHPMCGHEGE
B.anthracis_Tyr     78 VFA-SPVEET-KKLLHKLASFH--LRED-VIVTDVGSTKG-SIMNEAEALFSKE-ISFIGGHPMAGSHKT
S.thermophilus_     68 ILA-VPIKQT-MAYLKELADLD--LKDN-VIITDAGSTKR-EIVEAAERYLTGKNVQFVGSHPMAGSHKS
A.aeolicus_TyrA     67 MLS-SPVRTF-REIAKKLSYI---LSED-ATVTDQGSVKG-KLVYDLENILG---KRFVGGHPIAGTEKS
R.pneumotropicu    146 IVS-VPIANT-LETIERLKPY---LTEN-MLLADLTSVKR-EPLEKMLEIHQ---GAVVGLHPMFGPDIA
s001                58 IVS-VPINLT-LETIERLKPY---LTEN-MLLADLTSVKREEPLAKMLEVHT---GAVLGLHPMFGADIA
H.influenzae_Ty    149 IVS-VPINIT-LETIERLKPY---LTEN-MLLADLTSVKR-EPLEKMLEIHS---GAVLGLHPMFGSDIA
A.succinogenes_    146 MVC-VPIAKT-LETIERLKPY---LTES-MLLTDLTSVKR-RPLEKMLEVHT---GAVVGLHPMFGPDIA
H.massiliensis_    146 IVS-VPIVNT-VETIERLKPY---LTET-MLLTDLTSVKR-APLAKMLEVHR---GPAVGLHPMFGPDIT
H.somni_TyrA       146 IVC-VPIAQT-LNVIDRLKPY---LTEN-MLLTDLTSVKR-QPLEKMLQVHQ---GAVLGLHPMFGPDIT
A.actinomycetem    146 IVS-VPIANT-LAVIEQLKPY---LTEN-MLLVDFTSVKR-TPLEKMLEVHQ---GAVVGLHPMFGPDVV
A.aphrophilus_T    146 IVS-VPIANT-LEVIAQLKSY---LTEN-MLLADLTSVKR-APLEKMLEVHH---GPVVGLHPMFGPDVA
N.archeti_TyrA     147 IVS-VPIHLT-EQVIARLPPL----PAD-CILVDLASVKN-RPLQAMLAVHE---GPVVGLHPMFGPDVG
Y.kristensenii_    147 IVS-VPIHIT-EEVIARLPKL----PPD-CILLDLASVKN-KPLQAMLAAHE---GPVVGLHPMFGPDVG
S.marcescens_Ty    147 IVS-VPIHVT-EQVISRLPPL----PDD-CILVDLASVKN-RPLHAMLAVHS---GPVVGLHPMFGPDVG
E.coli_TyrA        147 IVS-VPIHVT-EQVIGKLPPL----PKD-CILVDLASVKN-GPLQAMLAAHD---GPVLGLHPMFGPDSG
C.youngae_TyrA     147 IVS-VPIHVT-EQVIAKLPRL----PSD-CILVDLASVKN-GPLQAMLAAHD---GPVVGLHPMFGPDSG
S.boydii_TyrA      147 IVS-VPIHVT-EQVIGKLPPL----PKD-CILVDLASVKN-GPLQAMLAAHD---GPVLGLHPMFGPDSG
C.freundii_TyrA    147 IVS-VPIHIT-EQVIAKLPRL----PSD-CILVDLASVKN-GPLQAMLAAHD---GPVVGLHPMFGPDSG
E.fergusonii_Ty    147 IVS-VPIHVT-EQVIGKLPPL----PKD-CILVDLASVKN-GPLQAMLAAHD---GPVLGLHPMFGPDSG
S.enterica_TyrA    147 IVS-VPIHVT-EQVIAQLPPL----PSD-CILVDLASVKS-GPLQAMLAAHD---GPVLGLHPMFGPDSG
C.sakazakii_Tyr    147 IVS-VPVHLT-EAVIRKLPAL----PQD-CVLVDLTSVKN-VPLQAMLEAHS---GPVLGLHPMFGPDSG
X.hominickii_Ty    147 IVS-VPIHLT-EEIIHRLPPL----PEQ-CILVDLASIKQ-RPLNAMLEVHK---GPVLGLHPMFGPDVG
P.asymbiotica_T    147 IIS-VPIHLT-EEVIRRLPPL----PDH-CILVDLASVKQ-QPLQAMLDVHK---GPVLGLHPMFGPDVG
M.fervens_TyrA      67 IVA-VPINVT-ERVIKEIAPH---VREE-CLLMDITSIKE-IPKRAMEENVKEG-VTVIPTHPMFGPSTP
M.maripaludis_T     67 IIS-VPINVT-ENVIKEVAPH---VKVG-SLLIDITSIKE-RPSELMKEFSKSG-VFVLPTHPMFGPETP
M.jannaschii_Ty     68 IVA-VPINVT-ERVIKEVAPH---VREG-CLLMDITSIKE-IPSKAMEEHVKEG-VTVIPTHPMFGPSTP
M.vulcani_TyrA      61 IVT-VPINIT-ERVISETAPK---MKSG-SLLMDFTSLKV-GPTEAMKKYAPKD-VEILGTHPMFGPSIP
M.burtonii_TyrA     61 IIT-VPIDIT-ADVIRETAPK---MKAG-SLLMDLTSIKA-EPVRAMRETAPDG-VEILGTHPMFGPSIP
M.thermophila_T     59 VLS-VPIDRT-PEVAGRIGPA---MRSG-SLLMDLTSLKV-EPVRAMVESTHPD-VEVLGAHPMFGPTMP
M.concilii_TyrA     62 LVS-VLIEKT-VEVIRQVAPR---MHSG-SLIMDVTSVKS-GPVRAMKTYAPKG-VEALGTHPMFGPTMP
M.harundinacea_     71 LLS-VPIKET-AKVVEEVAPR---MRPG-SLLMDVTSLKA-EPMKAMLRWAPDG-VEVLGTHPMFGPTIP
M.barkeri_TyrA1     80 IVS-VPINVT-EETIAEFAPK---MKSG-SLLMDFTSIKV-KPVEAMKKFAPSD-VEILGTHPMFGPTIP
M.barkeri_TyrA2     80 IVS-VPISVT-EETIAEIAPK---MKAG-SLLMDFTSIKV-KPVEAMRKFAPSG-VEILGTHPMFGPTIP
M.horonobensis_    100 IVS-VPINAT-EETIAEVAPK---MKAG-SLLMDFTSTKV-RPVEAMRRFAPGD-VEILGTHPMFGPTIP
M.mazei_TyrA        70 IVS-VPINAT-EETIAEIAPK---MKAG-SILMDFTSIKV-GPVEAMRKFAPKD-VEILGTHPMFGPTIP
C.methanoperede     61 MIS-VPINIT-EKTISNIAPR---MSPG-SLLMDITSIKT-GPMEAMIKYAPRN-VEILGSHPMFGPSIP
P.homiensis_Tyr     54 ILA-VPLSQM-QGVLGAIAPH---LRPG-ALVADVASVKL-RPAQMMRDLLPDH-VQILATHPLFGPESA
R.pomeroyi_TyrA     78 ILA-MPVAGI-AEACRAIAPH---LRPG-AVVADVGSVKM-TPAAIMQATLPGH-VALIGTHPLFGPQSA
M.hallyeonensi_     67 LIA-APVSSF-ENVVSEIADA---CNPG-ALIVDVGSVKV-VPAEIMRRMLPNH-VDIVATHPLFGPQSA
J.pohangensis_T     67 LVA-APVSSF-EQVVSEIAVA---CKPG-ALIVDVGSVKV-IPSEIMRRLLPNC-VDIVATHPLFGPQSA
R.halocynthiae_     67 LIA-APMSSF-EQVVSEIAVA---CKPG-ALIVDVGSVKV-VPAEIMRRLLPNH-VDIVATHPLFGPQSA
P.ascidiaceicol     67 LIA-APVSSF-EQVVSEIAVA---CKPG-ALVIDVGSVKV-VPSEIMRWLLPDN-VDIVASHPLFGPQSA
O.pituitosum_Ty     67 ILA-TPVSTL-EGVVRAIAPH---LRPG-ALVMDVGSVKV-GPTNIMLKGLPDH-VDIVATHPLFGPQSA
O.anthropi_TyrA     66 ILA-TPVATL-ERVVAMIAPH---LRPG-TLVLDVGSVKV-GPADIMRRGLPAH-VDIVATHPLFGPQSA
O.intermedium_T     74 ILA-TPVATL-ESVVDKIAPH---LCPG-ALVLDVGSVKV-GPADIMRRGLPAH-VDIVATHPLFGPQSA
B.canariense_Ty     68 ILA-VPVNRF-EESVAAISPH---LKPG-SLVLDVGSVKM-IPAEIMRRGLPEH-VAIIATHPLFGPQSA
P.inhibens_TyrA     64 ILA-VPVGAM-EPLCHTLAPL---VRPG-TWVLDVGSVKM-APADVMQRVLPPE-VNLLGTHPLFGPEST
P.zucineum_TyrA     59 VVA-VPVAAM-REVFAAIAPH---VKPG-ALVVDVGSVKV-LPARWMAELLPAH-ADLVATHPLFGPQSV
S.spMCT13_TyrA      57 MLA-VPVQAM-AGVIARIAPV---VRPG-ALVLDVASVKM-LPARWMAEALPPS-VDIVATHPLFGPQSA
A.excentricus_T     79 ILA-TPIRTL-KDLAAKIAPH---VPLN-GLVIDVGSVKV-KPAAWLQDVLPPQ-VSILCTHPLFGPQSA
S.cellulosum_Ty     73 LVA-VPVPAV-REALLALRPH---LAPG-QLVADVGSVKS-VPASAMAEVLGEG-RPWVATHPLFGPASL
M.xanthus_TyrA      58 VLS-MPVSGM-RSVLEELRPR---LSPA-QTVLDVGSVKV-RPVQVLASVLGRD-IPWVGTHPLFGPASL
C.bacteriumRBG_     62 LVS-VPMDSF-EDVIRLYAPH---IRPE-QTVIEITSVKV-KPVEAMHRYLKT--EKVLGVHPMFGPGAR
D.mccartyi_TyrA     62 IIS-VPIDAF-EDTLRELAPF---TKPD-QLVFDLCSVKE-RPVELMHQYLPH--CRTLGTHPVFGPGAE
D.mccartyi_TyrA     62 VIS-VPIDAF-EDTLKEIAPF---TKRE-QLVFDLCSVKE-RPVEMMHQYLPH--CRILGTHPVFGPGAK
D.spWBC-2_TyrA      62 VIS-VPVDSF-EAVCHNLAPH---LNSG-QTVFDLTSVKV-KPVDIMHRYFTG--IKVLGSHPVFGPGAE
D.lykanthropore     63 IIS-VPVDVF-GRVCEQLAPH---IRPG-QLVFDLTSVKV-MPVAAMHRNFNR--AQVLGVHPAFGPGAE
D.alkenigignens     68 IIS-VPIDSF-DFVCSGLAPH---VKPI-QKVFDLTSVKT-GPVTAMHRHFPH--SLILGAHPVFGPGAK
A.sulfaticallid     57 FLC-VPMDSI-DSVISKLEKLSTKFYKK-PLIVDIASVKS-DLDKLIRN------FDVLSIHPMIGPDSD
A.profundus_Tyr     50 FVC-VPMECI-KDALEDIKKY---ANKN-ALLVDISTIKS-DILPYFDESG----FDYMSIHPMFGPKSD
A.veneficus_Tyr     52 FLC-VPMQAV-GDAIARIPRSS-------ALIVDISSIKG-FVLPYLDASG----FDYLSIHPMFGPDSE
G.ahangari_TyrA     50 FLC-VPMDEI-ENALEDIAR----KNRS-ALIVDISTLKN-LAIPHLERLG----FDYLSIHPMFGPDSE
F.placidus_TyrA     50 FLC-VPMDKI-EDAVSEIKET---ADKN-ALLVDIASVKK-PFVKIYESSG----FEWMSIHPMFGPDSE
```

```
B.spBAL6_X_TyrA      58  IPF-VPMNKF-ESVIEEIAPL---LSKD-ALIVDVCSVKE-MPVNVMKKHLPET-VSILATHPMFGPDSA
B.spMedPE-Swde_      57  IPF-VPMSTF-ENTIKQMAPL---LKKD-TLVVDVCSVKE-MPVNIMKEHLSKD-IQIMATHPMFGPDSA
H.marinus_TyrA       57  ILC-VPISAI-ESIADQIAPL---VSKE-TLVVDVCSVKI-HPMKILEEKLPQD-TQILGTHPMFGPDSA
B.bacterium_Tyr      57  IPA-VPISEF-ESVIRQLSPL---LKPG-SIVADVCSVKE-HPAEIMQKYLPAH-TSILATHPMFGPDSA
D.bacterium4572      56  ILS-VPISNM-REMLRRIAPL---LRPD-AMVVDVCSVKV-YPIEWMRDALPAS-VSLLGTHPMFGPDSA
D.multivorans_T      56  ILS-VPISRM-ADFLKEIAPL---VVPD-AVVVDVCSVKV-YPVQWMTTLLPPS-VDILATHPMFGPDSA
D.bacterium_Tyr      67  IPT-VPISTL-EDILKKIAPL---LKKD-ALVIDVCSVKE-YPIKWMKELLPET-VSILATHPMFGPDSA
D.cetonica_TyrA      58  ILC-MPISAM-RETLRRVAPL---LSPD-ALVVDVCSVKV-YPVQWMRELLPEN-VSILPTHPMFGPDSA
D.tepidiphila_T      57  IVC-VPISAM-QATLAQIAPL---LRPG-VTVADVCSVKE-YPARWMRQLLPAS-VEILATHPMFGPDSA
S.africana_TyrA      55  FLC-TSIASV-GSVAETIASR---LHPR-TIVADTCSVKV-EPLADLDRVIPAE-NPLLGTHPMFGPDSA
S.bajacaliforni      61  FYC-VAISSF-EEVLSHTAAK---IGKH-TLVFDTCSVKV-EPLRTMERLLPRG-CTFAGSHPMFGPDSA
S.smaragdinae_T      58  FYC-VAISSF-EEVLSRTAAK---IGKH-TLVFDTCSVKV-EPVRTMERLLPQG-CTFAGSHPMFGPDSA
S.bacterium_Tyr      55  CLC-VDIGAM-PEVLARIAPR---LGPG-TLVLDTCSVKE-LPVRWMRELLPPG-VEILASHPMFGPDSG
S.bacteriumRBG_      55  FFC-VAISAF-EEVLVRTASR---IPSG-TLVMDTCSVKS-HPAELMRRLLAPT-VSVLATHPMFGPDSA
S.odontotermiti      55  ILC-VTISAM-EEVLEKIAKL---LAPG-TLVMDTCSVKV-YPANLMLRLLPPE-IEIIATHPMFGPDSG
S.bacterium4572      58  FLC-NAISSM-EEVLKDISGH---LQPG-TLVVDSCSVKV-YPINLMKAILPEE-VGILGTHPMFGPDSA
C.Bathyarchaeot      61  LIC-VPIENF-EEVVKEIHSH---VRSE-QVVMDICSIKE-IPVKIMHEYIKA--GITLGTHPVFGPGVK
M.truncatula_3g      68  LLC-TSISSL-SEVVGSMPLAC--LKRP-TLFVDVLSVKE-HPKNLLLKVLPEE-SDILCTHPMFGPVSG
s002                 64  VLCTTSILSLSSEVVGSMPLTS-SLKRP-TLFVDVLSVKE-HPRELLLRELPEDSSDILCTHPMFGPQTA
G.max_11g233900      73  VLC-TSILSL-SEVVGSMPLTS--LKRP-TLFVDVLSVKE-HPRELLLRELPED-SDILCTHPMFGPQTA
G.max_18g023100      70  VLC-TSILSL-SEVVGSMPLTS--LKRP-TLFVDVLSVKE-HPRELLLRELPED-SDILCTHPMFGPQTA
B.bituminosa_20      73  VLC-TSILSL-SEVVGSMPLTS--LKRP-TLFVDVLSVKE-HPREVLLRVLPED-SDILCTHPMFGPQTA
P.vulgaris_1g24      71  VLC-TSILSL-SEVVGSMPLAS--LKRP-TLFVDVLSVKE-HPRELLLRELPEH-SDILCTHPMFGPVSG
L.sativus_20036      66  LLC-TSISSL-SEVVGSMPLTC--LKRP-TLFVDVLSVKE-HPRNLLLKVLPEE-LDILCTHPMFGPVSG
A.americana_205      66  LLC-TSISSL-SEVVGSMPLTR--LKRP-TLFVDVLSVKE-HPRNLLLQVLPEE-SDILCTHPMFGPVSG
M.truncatula_5g      67  LIC-TSIMSF-TKVLSSMPLAC--LKKPTTLFVDVLSVKE-HPREVLLRVLPEE-SDILCTHPMFGPESG
G.max_14g055300      66  LVC-TSILSL-SEVLSSMPLTC--LKRS-TLFVDVLSVKE-HPRNLLIKVLPEE-SDILCTHPMFGPDSG
C.cajan_05579        62  LIC-TSILSL-SKVLTSMPLTP--LKTP-TLFVDVLSVKE-HPRDLLLRVLPEE-SDILCTHPMFGPDSG
P.trichocarpa_8      82  LIC-TSILSL-SKVLNTMPLHC--LKRS-PLFVDVLSVKE-YPRDILLKVLPEE-LDVLCTHPMFGPESG
C.clementina_32      72  LIS-TSILSL-SEVLNSLPVHC--LQRR-TLIADVLSVKE-YPRNVLLQVLPEE-MDVLCTHPMFGPESG
S.lycopersicum0      72  MIS-TSILSL-SQVVESIPFNC--LKRP-TLFVDVLSVKE-HPKDVLLRMMPRE-CDLLCTHPMFGPESG
A.trichopoda_00      89  LHC-TLIIST-ESVLRSLPTQR--LKRN-TLFVDVLSVKE-IPKNLFLQILPRE-FDVLCTHPMFGPESG
S.fallax_TyrA       182  LLC-TSILST-EDVLKLLPVQR--LKRN-TLFVDVLSVKE-FPQKLFLQVLPQE-FDVLCTHPMFGPESG
S.lycopersicum0     140  VLC-TSIIST-ETVLRSLPIQR--LKRN-TLFVDVLSVKE-FPKNIFLQVLPTH-FDILCTHPMFGPESG
A.trichopoda_00     110  LLC-TSIIST-ESVLRSLPTQR--LKRN-TLFVDVLSVKE-FPRNLFLQILPQE-FDVLCTHPMFGPESG
P.patens_TyrA2      170  LLC-TSILST-VAVLQSLPLQR--LRRN-TLFVDVLSVKE-FPKNLFLQALPAE-FDVLCTHPMFGPESG
P.patens_TyrA1      161  LLC-TSILST-EAVLQSLPLQR--LRRH-TLFVDVLSVKE-FPKNLFLQVLPPE-FDILCAHPMFGPESG
A.thaliana_1g15     119  LLC-TSILST-ESVLRSFPFQR--LRRS-TLFVDVLSVKE-FPKALFIKYLPKE-FDILCTHPMFGPESG
S.lycopersicum0     158  LLC-TSINSL-ENVIRSLPIQK--LKRN-TLFVDVLSVKE-FPKNIFLQSLPKE-FDILCTHPMFGPTSG
B.distachyon_1g     141  LLS-TSILSA-EAVLRSLPVHR--FRRS-TLFADVLSVKE-FPKNLLLAYLPGD-FDVICTHPMFGPESA
S.italica_4g275     142  LLA-TSILSA-EAVLRSLPVHR--LRRN-TLFVDVLSVKE-FPKNLLLSSLPPD-FDVICTHPMFGPESA
S.italica_4g174     155  LIC-SSILST-ESVVSAIPFHK--LRPD-TIVADVLSVKQ-FPRNLLLEILPPG-FGIVCTHPMFGPESG
B.distachyon_1g     154  LVC-SSILST-EAVVRAIPFHK--LRSD-TIVADVLSVKQ-FPRNLLLEILPPE-FGIVCTHPMFGPESG
A.trichopoda_00       1  ----------------------------MLMVDVLSVKE-FPRDHFLQVLSQE-FDILCTHPMFGPESG
A.trichopoda_00     141  LVC-TSILSA-ENILRSIPVHK--LKPD-TIFADVLSVKQ-FPKNLFLEILPPE-FGIICTHPMFGPESG
C.reinhardtii_0     147  LLA-SSILST-EKVLRSVPVQR--LKRN-TLFVDVLSVKV-FPKQLLLRELPSE-VDILCTHPMFGPDSG
C.subellipsoide      88  IFA-SSILSM-GSVLGGLPVQR--LKRN-TLFVDVLSVKE-FPKRLMLSTLPSE-VDILCTHPMFGPDSG
O.lucimarinus_T     116  VVC-TSILST-DATLRNFPLQR--LKRS-TLFCDVLSVKQ-FPKQLFQQLLPPD-FDILCLHPMFGPDSG
C.merolae_TyrA       71  VLS-TSILST-EEVLRRFPTA---KLRS-CLVVDVLSVKV-YARELMLRYAPEE-ADILATHPMFGPESG
G.sulphuraria_T      68  LFC-TSITST-RSVIEAFPID---ALRG-KLVVDVLSVKA-YPKKLLLELLPPE-ADVLCTHPMFGPESG
S.cryophilus_Ty      75  LYS-VESEYL-DRIVGMYGPA---TKVG-AIVGGQSSCKS-PEIRAFEKYLPKD-VEIISCHSMHGPRVN
P.decumbens_Tyr      72  LYS-VEAGVI-DRVVAQYGPS---TKVG-AIVGGQTSCKG-PELAAFEKHLPED-VEIVSCHSLHGPKVN
A.carbonarius_T      72  IYS-VEAGVI-DKVVAMYGPS---TKVG-AIVGGQTSCKA-PELSAFDKHLPQD-VEIVSCHSLHGPQVN
S.complicata_Ty      80  MYS-VEAEYI-DRVVAMYGPS---TKLG-AIVGGQTSCKA-PEIAALEEHLPPD-TQIVSCHSLHGPSVS
S.japonicus_Tyr      79  IYS-VEAEKI-DAVVGKYGPA---TKMG-AIVGGQTSCKT-PEIAAFEKYLPED-VQIISCHSLHGPAVN
S.octosporus_Ty      75  LYS-VESEYL-DRIVGMYGPA---TKVG-AIVGGQSSCKS-PEIRAFEKYLPQD-VDIISCHSMHGPRVN
S.pombe_TyrA         68  LYS-VEAEHI-DKVVALYGPA---TKVG-AIVGGQTSCKA-PEMNAFEKYLPED-VDIISCHSMHGPKVN
```

```
s003              121 GIDGAEENLFVNAPYVLTPTE----YTDPEQLAX-LRSVLEPLGVK-IYLCTPADHDQAVAWISHLPVM-
S.spPCC6803_Tyr   121 GIDGAEENLFVNAPYVLTPTE----YTDPEQLAC-LRSVLEPLGVK-IYLCTPADHDQAVAWISHLPVM-
B.animalis_TyrA   151 GWVAADPTLFDDALWAVTFGL----HTQYRRVVQIAGMITRGVRNT-MIVIDDVTHDREASLISHMPHV-
B.pseudocatenul   127 GWESSDPTLYDGALWAITVDE----NTEYQRFRAVATMIVDHCSNR-LIVLDDATHDRCAALISHMPHV-
B.adolescentis_   127 GWEASDPTLYDDALWAITVDE----HTEYRRFRAVADMIVNRCGNR-LIVLDDATHDRCAALISHMPHV-
B.dentium_TyrA    127 GWESSDPALYDDALWAITVDE----RTEYRRFLAVATMITDACANR-LIVLDDATHDRCAALISHMPHV-
S.meliloti_TyrA   132 GPDAGFAGLFRGRWCILTPPA----GTDEEAVAR-LRLFWETLGSM-VDEMDPKHHDKVLAIVSHLPHI-
R.leguminosarum   132 GPDAGFPGLFEGRWCIFTPVA----GTDEAAMKR-LRSFWEALGSK-VDEMDAEHHDKVLAIVSHLPHI-
T.sp_TyrA         127 GYDNADADLFVNSNYLLTPSD----IVKEDILELFIDEVIIKIGAK-PVIMDYNKHDAIVGVISHVPHI-
D.invisus_TyrA    127 GLSRADPDIFRGANYILLPDK----TNRPESVEL-LRNMALALGCRHVPSITPEEHDRHIAYTSDLTHV-
B.anthracis_Tyr   141 GVESAKAHLFENAFYILTPMH----HVPNEHVEE-LKDWLKGTGSH-FLVLNTEEHDYVTGIVSHFPHL-
S.thermophilus_   132 GAIAADVTLFENAYYIFTPTS----LTKETTIPE-LKDILSGLKSR-YVEIDAAEHDRVTSQISHFPHL-
A.aeolicus_TyrA   127 GVEYSLDNLYEGKKVILTPTK----KTDKKRLKL-VKRVWEDVGGV-VEYMSPELHDYVFGVVSHLPHA-
R.pneumotropicu   206 --------SMAKQVVVCCDGR------FPERYEW-LLEQIQIWGVK-IHQTDAIEHDHNMTYVQALRHF-
s001              119 --------SMAKQVVVRCDGR------FPERYEW-LLEQIQIWGAK-IYQTNATEHDHNMTYIQALRHF-
H.influenzae_Ty   209 --------SMAKQVVVRCDGR------FPERYEW-LLEQIQIWGAK-IYQTDATEHDHNMTYIQALRHF-
A.succinogenes_   206 --------SMAKQIVVRCDGR------YPERYEW-LLQQIGIWGAK-IYQADAAEHDHSMTYIQALRHF-
H.massiliensis_   206 --------SMAKQVVVCCDGR------YAVRYQW-LLEQIQIWGAK-IYQVDAAEHDRSMTYVQALRHF-
H.somni_TyrA      206 --------NMAKQVVVRCDGR------YPEKYQW-FLEQIQMWGAK-IYQVDATEHDKSMTYVQALRHF-
A.actinomycetem   206 --------SMAKQVVVCCDGR------FSERYQW-LLQQIQIWGAK-IYQVDAAEHDHHMTYIQALRHF-
A.aphrophilus_T   206 --------SMAKQIVACCDGR------FSERYQW-LLQQIQMWGVK-IYHVDATEHDHHMTYIQALRHF-
N.archeti_TyrA    206 --------SLAKQVVVYCDGR------EPEAYQW-LLEQLQVWGAR-LHRISAVEHDQNMAFIQALRHF-
Y.kristensenii_   206 --------SLAKQVVVYCDGR------SPQAYQW-LLEQLQVWGAR-LHRISAVEHDQNMAFIQALRHF-
S.marcescens_Ty   206 --------SVAKQVVVYCDGR------RPEAYQW-LLEQLQVWGAR-LHRISAVEHDQNMAFIQALRHF-
E.coli_TyrA       206 --------SLAKQVVVWCDGR------KPEAYQW-FLEQIQVWGAR-LHRISAVEHDQNMAFIQALRHF-
C.youngae_TyrA    206 --------SLAKQVVVWCDGR------QPEAYQW-FLEQIQVWGAR-LHRISAVEHDQNMAFIQALRHF-
S.boydii_TyrA     206 --------SLAKQVVVWCDGR------KPEAYQW-FLEQIQVWGAR-LHRISAVEHDQNMAFIQALRHF-
C.freundii_TyrA   206 --------SLAKQVVVWCDGR------QPEAYQW-FLEQIQVWGAR-LHRISAVEHDQNMAFIQALRHF-
E.fergusonii_Ty   206 --------SLAKQVVVWCDGR------KPEAYQW-FLEQIQVWGAR-LHRISAVEHDQNMAFIQALRHF-
S.enterica_TyrA   206 --------SLAKQVVVWCDGR------QPEAYQW-FLEQIQVWGAR-LHRISAVEHDQNMAFIQALRHF-
C.sakazakii_Tyr   206 --------SLAKQVVVYCDGR------MPEAYQW-FLEQIQVWGAR-LHRISAVEHDQNMAFIQALRHF-
X.hominickii_Ty   206 --------SFAKQVVVYCDGR------HPEAYQW-FLEQMSVWGAR-LHQISAEKHDKNMSFIQALRHF-
P.asymbiotica_T   206 --------SFAKQVVVYCDGR------QREAYQW-FLEQLLVWGAC-LYQINPEQHDKSMSFIQALRHF-
M.fervens_TyrA    129 --------SLLRQVVILTPSE----KHKQSEWFGKVYNFLKKEGAK-VIVIPAEKHDKIMGIVQGLTHF-
M.maripaludis_T   129 --------SLNRQVVILTPIE----KEKNPFFEK-VKEFLENEGAK-VIVVSPKEHDKIMGVVQGLTHF-
M.jannaschii_Ty   130 --------SLLRQVVILTPSE----KHKNTEWFNKVYNFLKKEGAK-VIVIPPEKHDRIMGIVQGLTHF-
M.vulcani_TyrA    123 --------SLHGQIFILTPIE----GRCNKWFPI-MRSLFEDNGAH-IEVITPTEHDRFVSVVQGLTHF-
M.burtonii_TyrA   123 --------TLQGQIVIMSPTK----GRSEKWFPI-MRNLFEENGAH-IEIIKPEEHDKFVSVVQGLTHF-
M.thermophila_T   121 --------SIRGQTVIITPVE----GRWGRWSSH-IREILERDGAR-VEVLTPEEHDRMMAVVQALTHF-
M.concilii_TyrA   124 --------SLFGQTIIFTPVE----GKTGKWLGV-IRSLFESDGAK-IEILEAEEHDETMAVVQALTHF-
M.harundinacea_   133 --------TIRGQTVILVPAA----GRCDEWLLP-MEEIFQEDGAR-VEVLGAEEHDRIMAVVQALTHF-
M.barkeri_TyrA1   142 --------TIRGQTVILVPVK----GRSEKWFPV-IRELFEEGGAH-VEITTAAEHDRLVSVVQGLTHF-
M.barkeri_TyrA2   142 --------TIRGQTVILVPVK----GRSEKWFPV-IKELFEEGGAH-VEITTAAEHDRLVSVVQGLTHF-
M.horonobensis_   162 --------TIRGQTVILVPVE----GRSEKWFPV-IRQLFEENGAH-VEITTAAEHDRLVSVVQGLTHF-
M.mazei_TyrA      132 --------TIRGQTVILVPVK----GYSEKWFPV-IRQLFEESGAH-VEITTAEEHDRLVSVVQGLTHF-
C.methanoperede   123 --------DIRGQIVIFTPAN----ERSSKWLPL-IKSLYEDNGAH-IEILDALEHDRMMAVIQGLTHF-
P.homiensis_Tyr   116 ------RDGIAGHRIAWCPLR------GRG-HLR-LARFLRGLGLR-VIPTTPDDHDREMAVVQGLTHL-
R.pomeroyi_TyrA   140 ------RHGIAGHKIALCPLR------GRAHLPV-AAFLRARLGLR-VILTTPEAHDREAATVQGLTHL-
M.hallyeonensi_   129 ------RQGIEGLKVAVCPVQ------GNRHAQV-AAFLRKALGLT-VIMTTPEDHDQEAAVVQGLTHL-
J.pohangensis_T   129 ------GTGIEGLKVVVCPIR------GRRHARM-AAFLRKALRLT-VIVTTPEAHDKEAAVVQGLTHL-
R.halocynthiae_   129 ------GTGIEGLKIAVCPIQ------GTRHARL-AAFLHKALGLN-VIMTTPEYHDQELAVIQGLTHL-
P.ascidiaceicol   129 ------TTGIKGLKIAVCPIQ------GKRHARL-VAFLRKILGLT-VIVTTPEDHDQEAAVVQGLTHL-
O.pituitosum_Ty   129 ------RNGIAGMKIAICPVR------GTQALRI-GAFLRKQLALD-VIMTTPEDHDRDAAMVQGLTHL-
O.anthropi_TyrA   128 ------RDGIAGLKIAVCPVR------GTRFHRV-AAFLKRKLALD-VIITTPEDHDREAAMVQGLTHL-
O.intermedium_T   136 ------RHGIAGLKIAVCPVR------GTRFHRV-AAFLKKHLSLN-VIVTTPEDHDREAAMVQGLTHL-
B.canariense_Ty   130 ------RDGINGLKIAICPIR------GDRGRRV-AAFLRKALGLN-VVMTTPEAHDQDTAMAQGLTHL-
P.inhibens_TyrA   126 ------RQGLAGQKIALCPLR------GGRPLRL-AAVLRHIFRLE-VIWTTPEAHDRELATVQGLTHL-
P.zucineum_TyrA   121 -----ARDGLPGLRFVVCPIR-------GDRYER-VAALGRELGLS-VTITTPEEHDEEMAYVQALTHL-
S.spMCT13_TyrA    119 ------RNGLTGQPLVVCPVR-------GNRHEK-VAAFGRSLGLK-VSITTAEEHDREMAYVQALTHL-
A.excentricus_T   141 ------RYGIHDMEIVVCPVR------VRHLSPI-VRFLEKTLDLK-VSIATPEIHDRALAAVQGLTHM-
S.cellulosum_Ty   135 ------ARGERPLRVVVCPNP-----THPGAVRR-VVALFERIGCQ-VLEQAPEEHDRVMAFTHALAFF-
M.xanthus_TyrA    120 ------ARGDLPRRTVVCPNE-----LHPEAVRK-ARVLFERIGCE-VTELSPDAHDALMVRTHVLTFF-
C.bacteriumRBG_   123 --------DMANQNFILTPTG----TAEDALAEK-VKAYLEKRGGR-VRVMTPEQHDETMSIVLGLPHI-
D.mccartyi_TyrA   123 --------SLKGYNFILTPTT----APETDLAQG-VKTWLEKQGGN-VSLISPEEHDRLMSVVLGLAHF-
D.mccartyi_TyrA   123 --------SLKGYNFILTPTT----EAETALAEK-VKTWLEKHEST-VSLTSPEEHDRLMSVVLGLAHF-
D.spWBC-2_TyrA    123 --------SLKGQNFILTPTD----DIEKATAFK-VSDWLSTRGAR-VRTTTPEEHDRLMSISLGLAHF-
D.lykanthropore   124 --------SLNGQNVILTPTD----EAENRLADE-VRDWLTAQGAR-VRVTSPEEHDRLMSISLGLAHF-
D.alkenigignens   129 --------GLAGQNFILTPTS----DAENELARK-LSHWLTGRGAR-VRLTSPEEHDRLMAISLGLAHF-
A.sulfaticallid   117 --------LGLSNIIVVYES-------GREEGKL-ILDELYRAGAN-ISRLNYRLHDHKMAEIQGVAHF-
A.profundus_Tyr   109 --------IGL-SNIIVVHRS------GRREEEV-ILEEFKKAGAV-ISYLPRERHDVVMAEIQGLSHF-
A.veneficus_Tyr   108 --------IGL-SNIIVVKKS------GRKEEEV-ILEEFRKAGAV-LSELPPEKHDAKMAEIQGLAHF-
G.ahangari_TyrA   108 --------IGL-SNIIVVHES------GREEERV-ILEEFRRAGAI-ISHISVEEHDRKMAEIQGTAHF-
F.placidus_TyrA   109 --------IGL-SNVIVVRLP------KDERAVR-IVEEFRKSGAI-VSFLSLEEHDEEMAKIQSTSHF-
```

```
B.spBAL6_X_TyrA   120 ------GETLFGTKIAVCPVR-----IEDKYYQK-ICAFMRFHGIK-VIETTAAEHDKQISHSLLLTHM-
B.spMedPE-Swde_   119 ------RDTLFGTKIAVYPVR-----MEQELYIK-ISAYLRSHGIK-TIEASPEDHDKQIANSLILTHL-
H.marinus_TyrA    119 ------AKSLYGCKIVLCQQR-----VSDERYRN-IKGYLESHGLK-VIEATADEHDQQIANTLILTHT-
B.bacterium_Tyr   119 ------SDSFFARKLVLCPIR-----MEEARYQS-IKRYLQEQGLT-VIETTPAHHDEEISQSLLLAHF-
D.bacterium4572   118 ------ADSLHGHKIVLCKDR-----VNASCYGK-VKAYLTGKGLK-VIDATPEEHDRQIAVSLSLTHF-
D.multivorans_T   118 ------ATSLLDRKIVLCKTR-----ISDARYDK-IKSYLKARGLV-VIETTPEEHDRQIAMSLCLTHF-
D.bacterium_Tyr   129 ------ADSLKERKIVLCKVR-----IEEKFYNK-IKSSLSSKGLT-VIETTPEDHDRQIAVSLSLTHF-
D.cetonica_TyrA   120 ------ADALVGRKIVLCPER-----IDEDQYDR-IRRWLESKGLV-VIRTTAREHDEKIAVSLSLTHF-
D.tepidiphila_T   119 ------AHSLQGHKIVLCPER-----IRPERYEK-IKAWLSARQLQ-IIETTPAEHDRKIALSLSLTHF-
S.africana_TyrA   117 ------RNGADGLPMVFSPVR-----IAPDQLAM-VQDFFRKYQLD-QLVMTPEEHDREAAYTQGITHF-
S.bajacaliforni   123 ------SEGMDGLPVVLCPGR-----DDEGKVDF-WRHHFSKMGLR-VIEITADRHDKEAAYTQGITHV-
S.smaragdinae_T   120 ------SEGMDGLPMVLCPGR-----DDEGKVDF-WRHHFSKMGLR-VIEITADRHDKEAAYTQGITHV-
S.bacterium_Tyr   117 ------ARGVEGLPMILCPVR-----LQEARYRE-WEGFFSSFGLE-VHRLAPEDHDRQAAYTQGLTHY-
S.bacteriumRBG_   117 ------TESLDGLPMVLCPVR-----ITPEELER-WRRFFASLGLS-VAVMDPDEHDRQAAFTQGVTHY-
S.odontotermiti   117 ------VDGITGLPMVFSPMR-----VDERIAQL-WSSNFEAMGLK-VIDMSPKQHDEEMAFSQGITHF-
S.bacterium4572   120 ------KISVEGLPIVLCAER-----LRDEQYVY-WKDRFRAMGLV-IQEMTADEHDREAAYTQGITHF-
C.Bathyarchaeot   122 --------SIKNQNFILTPTV----EKEEVFAEN-FKSWLEERGAN-VFIMSPRRHDELMSVVLGLPHF-
M.truncatula_3g   131 ------KNGWQNLTFMFDKVR----IKDEVTCSK-FLQIFASEGCK-MVEMSCEEHDKAAAKSQFITHT-
s002              131 ------KNGWTDHTFMYDKVR----IRDEVICSSNFIQIFATEGCK-MVQMSCEEHDRAAAKSQFITHTI
G.max_11g233900   136 ------NNGWTDHTFMYDKVR----IRDEATCSS-FIQIFATEGCK-MVQMSCEEHDRAAAKSQFITHT-
G.max_18g023100   133 ------KNGWTDHTFMYDKVR----IRDEVICSN-FIQIFATEGCK-MVQMSCEEHDRAAAKSQFITHT-
B.bituminosa_20   136 ------KNGWKDHTFMFDKVR----IRDEATCSN-FIQIFASEGCK-MVQMSCEEHDRAAAKSQFITHT-
P.vulgaris_1g24   134 ------KNGWKGLTFMYDKVR----IRNEAICSS-FIQIFASEGCK-MVQMTCEEHDKAAAKSQFITHT-
L.sativus_20036   129 ------KNGWQNLTFMYDRVR----IKDEATCSK-FLQIFSSEGCK-MVEMSCEEHDRAAAKSQFITHT-
A.americana_205   129 ------KNGWKELTFMFDKVR----IRDQVTCSK-FLQIFETEGCK-MVEMSCEEHDKAAAKSQFITHT-
M.truncatula_5g   131 ------KNGWKDLNFMYDKVR----IHDEATCSN-FLHIFASEGCK-MLQMSCEEHDKIAAKSQFITHT-
G.max_14g055300   129 ------KDGWQDLTFVYDKVR----IRDEAICSS-FLHIFASEGCR-MLQMSCEEHDKIAAKSQFITHT-
C.cajan_05579     125 ------KDGWKDLTFVYDKVR----IRDEAICSN-FLQIFASEGCR-MLQMSCEEHDKLAAKSQFITHT-
P.trichocarpa_8   145 ------KNGWKDLAFMYERVR----IKDEATCSS-FLRIFETEGCR-MLEMSCEEHDMVAARSQFLTHT-
C.clementina_32   135 ------QNGWKDFAFVYEKVR----IRDEATCSS-FLRIFESEGCK-MLEMSCEEHDKVAAKSQFLTHT-
S.lycopersicum0   135 ------KDGWEDLTFMYDMVR----IRDQPLCSS-FLHIFSSEGCK-MLEMTCEKHDRLAAQSQFLTHT-
A.trichopoda_00   152 ------KDGWKGLPFVYEKVRIGSDEYREDRCQK-FLDIFAKEGCR-MVEMSCSEHDRHAAESQFITHT-
S.fallax_TyrA     245 ------KSSWVGLPFVYDKVRI-GGGSRLERCDR-FLNIFGSEGCR-MVEMSCAEHDRYAAGSQFITHT-
S.lycopersicum0   203 ------KDSWKDLIFMFDKVRIGEGRSRTARVDK-FLDIFEKEGCR-MVPMTCAEHDKHAAGSQFITHT-
A.trichopoda_00   173 ------KDGWKGLPFVYEKVRIGSDEYKEDRCQK-FLDIFAKEGCR-MVEMSCSEHDRHAAESQFITHT-
P.patens_TyrA2    233 ------KGSWAGLPFVYDKVRI-SNGRRSRIADR-FLDIFASEGCR-MVKMTCAEHDRYAAGSQFITHT-
P.patens_TyrA1    224 ------KGSWAGLPFVFDKVRV-SNGRRSRVADK-FLDIFSKEGCR-MVEMSCAEHDRFAAGSQFVTHT-
A.thaliana_1g15   182 ------KHSWSGLPFVYDKVRIGDAASRQERCEK-FLRIFENEGCK-MVEMSCEKHDYYAAGSQFVTHT-
S.lycopersicum0   221 ------KDNWKGLPFMYDKVRIGQEESRIKRVNN-FINIFVKEGCR-MVEMSCSEHDKYAAGSQFITHT-
B.distachyon_1g   204 ------RDGWAGLPFVFDEVRVGDGPARRARADA-FLDVFAREGCR-MVEMSCAEHDAHAAETQFLTHT-
S.italica_4g275   205 ------RDGWDGLPFVFDKVRVGDCPARRARAEA-FLNIFEREGCR-MVEMSCAEHDAHAAETQFLTHT-
S.italica_4g174   218 ------KHGWGKLPFVYNKVRVAEGGDQATKCDQ-FLSIFEQEGCR-MVEMSCAEHDRYAAGSQFITHT-
B.distachyon_1g   217 ------KHGWSTLPFVYDKVRLADKGDQKANCGQ-FLSIFEGEGCR-MVEMSCAEHDRHAAASQFITHT-
A.trichopoda_00    40 ------RNGWSGLPFMFDKVRISKDDHRSKICDD-FLHIFKLEGCR-MVEMSCEEHDQLAAQTQFITHT-
A.trichopoda_00   204 ------KNGWSGLPFVYDNVRIAQGTEQEKSCAK-FLSIFENEGCR-MVEMSCEEHDRHAAGSQFITHT-
C.reinhardtii_0   210 ------KGSWAGLNFMYEKVRIGADPKRERRVET-FLKFFRDEGCN-MVEMTCEEHDRQAASTQFITHT-
C.subellipsoide   151 ------KHSWQGLTLMYERVRVAGSADRQKRAAN-LLRFFETEGCR-MVEMSCEEHDRIAASTQFITHT-
O.lucimarinus_T   179 ------KHGWRDLPLVFDKVRIGTEPCREERCAR-LLGIFEAAGCR-MVEMSCEEHDRQAASSQFITHT-
C.merolae_TyrA    133 ------RGSWRGLPFVFERTR----VRQHERCEA-FLSIFRERGCT-LIEMPCQEHDHYAASTQFITHT-
G.sulphuraria_T   130 ------RYSWKGLPFVYEQVR----ISDGARCET-FLGIFSSALCT-MIPMSCELHDSYAASSQFITHT-
S.cryophilus_Ty   137 ---------PQSQPLVIIRHR-----AKDKSFMI-VREILSCLQSS-VVFLSAEEHDRITADTQAVTHA-
P.decumbens_Tyr   134 ---------PKGQPLVLIQHR-----ASDESVQF-VDRILSSFGSK-HVYLSGEMHDRITADTQAVTHA-
A.carbonarius_T   134 ---------PKGQPLVLIQHR-----ASDESLGF-VKEVLSCFESK-FVFLSGEMHDRITADTQAVTHA-
S.complicata_Ty   142 ---------PRGQPLVLISHR-----CTPEATSL-VRSILSCFDSK-IVELSAKEHDRITADTQAVTHA-
S.japonicus_Tyr   141 ---------PKSQPLIIIRHR-----ASDEAFAV-VNYMFQCFGSS-LAYLSAGEHDRITADTQAVTHA-
S.octosporus_Ty   137 ---------PQSQPLVIIRHR-----SKDKSFEI-VKEILSCLQSS-VVFLSAEEHDRITADTQAVTHA-
S.pombe_TyrA      130 ---------PKSQPLVIIRHR-----ASDEHFEI-VNEILSCFKSS-VVYLSAKEHDRITADTQAVTHA-
```

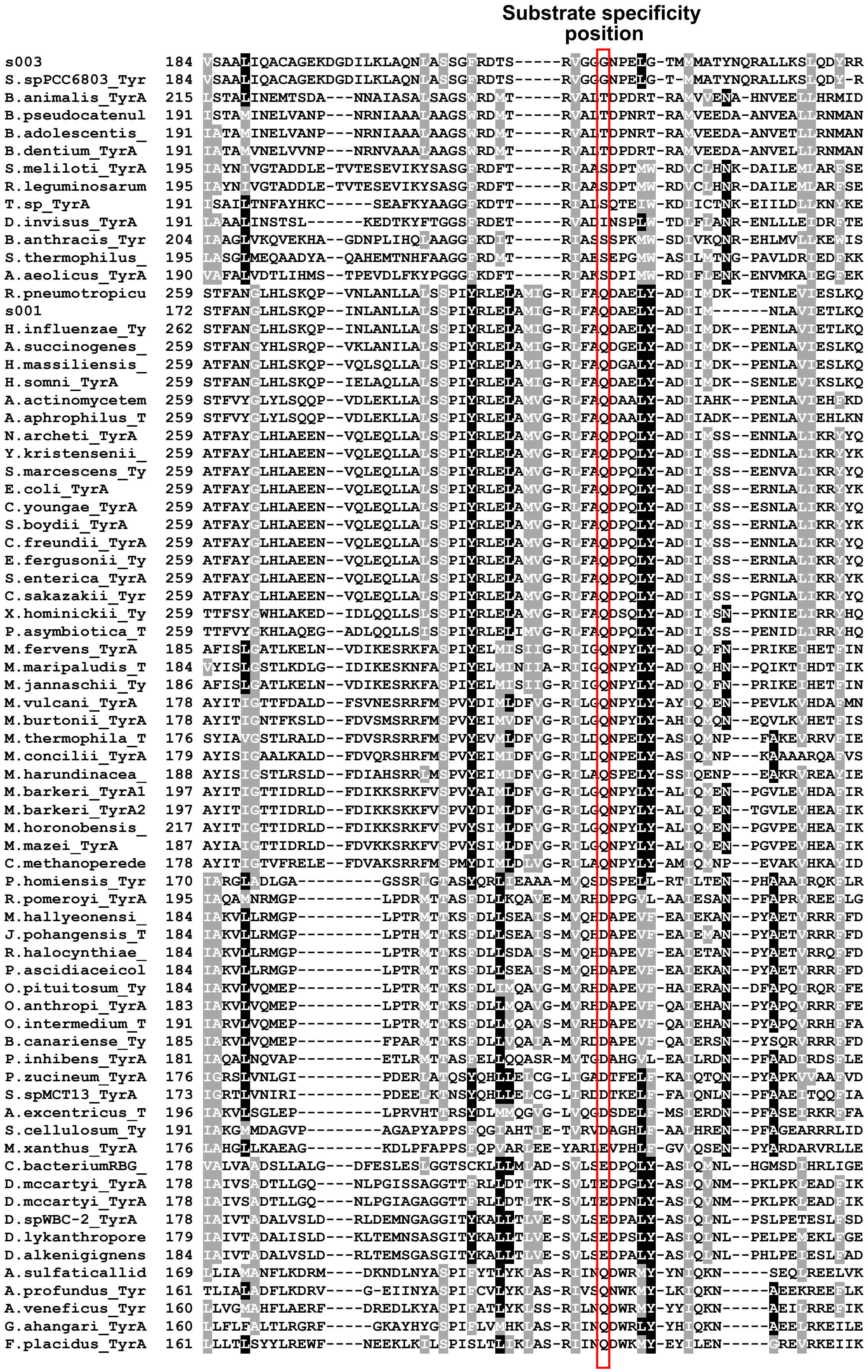
Substrate specificity
position
s003 184 VSAALIQACAGEKDGDILKLAQNLASSGERDTS-----RVGGGNPELG-TMMMATYNQRALLKSLQDYRR
S.spPCC6803_Tyr 184 VSAALIQACAGEKDGDILKLAQNLASSGERDTS-----RVGGGNPELG-T-MMATYNQRALLKSLQDY-R
B.animalis_TyrA 215 LSTALINEMTSDA---NNAIASAISAGSWRDMT-----RVAITDPDRT-RAMVVENA-HNVEELLHRMID
B.pseudocatenul 191 ISTAMINELVANP---NRNIAAAIAAGSWRDMT-----RVAITDPNRT-RAMVEEDA-ANVEALLRNMAN
B.adolescentis_ 191 IATAMINELVANP---NRNIAAAIAAGSWRDMT-----RVAITDPNRT-RAMVEEDA-ANVETLLRNMAN
B.dentium_TyrA 191 IATAMVNELVVNP---NRNVAAAIAAGSWRDMT-----RVAITDPDRT-RAMVEEDA-ANVELLLRNMAN
S.meliloti_TyrA 195 IAYNIVGTADDLE-TVTESEVIKYSASGFRDFT-----RIAASDPTMW-RDVCLHNK-DAILEMIARFSE
R.leguminosarum 195 IAYNIVGTADDLE-TVTESEVIKYSASGFRDFT-----RIAASDPTMW-RDVCLHNR-DAILEMIARFSE
T.sp_TyrA 191 ISAILTNFAYHKC-----SEAFKYAAGGFKDTT-----RIAISQTEIW-KDIICTNK-EIILDLLKNYKE
D.invisus_TyrA 191 LAAALINSTSL------KEDTKYFTGGSFRDET-----RVADINSPLW-TDLFIANR-ENLLLEIDRFTE
B.anthracis_Tyr 204 IAAGLVKQVEKHA--GDNPLIHQLAAGGFKDIT-----RIASSSPKMW-SDIVKQNR-EHLMVLLKEWIS
S.thermophilus_ 195 LASGLMEQAADYA--QAHEMTNHFAAGGFRDMT-----RIAESEPGMW-ASILMTNG-PAVLDRIEDFKK
A.aeolicus_TyrA 190 VAFALVDTLIHMS--TPEVDLFKYPGGGFKDFT-----RIAKSDPIMW-RDIFLENK-ENVMKAIEGFEK
R.pneumotropicu 259 STFANGLHLSKQP--VNLANLLAISSPIYRLEIAMIG-RIFAQDAELY-ADIIMDK--TENLEVIESLKQ
s001 172 STFANGLHLSKQP--INLANLLAISSPIYRLEIAMIG-RIFAQDAELY-ADIIM------NLAVIETLKQ
H.influenzae_Ty 262 STFANGLHLSKQP--INLANLLAISSPIYRLEIAMIG-RIFAQDAELY-ADIIMDK--PENLAVIETLKQ
A.succinogenes_ 259 STFANGYHLSRQP--VKLANILAISSPIYRLEIAMIG-RIFAQDGELY-ADIIMDK--PENLAVIESLKQ
H.massiliensis_ 259 ATFANGLHLSKQP--VQLSQLLAISSPIYRLEIAMIG-RIFAQDGALY-ADIIMDK--PENLAVIESLKQ
H.somni_TyrA 259 STFANGLHLSKQP--IELAQLLAISSPIYRLEIAMIG-RIFAQDAELY-ADIIMDK--SENLEVIKSLKQ
A.actinomycetem 259 STFVYGLYLSQQP--VDLEKLLAISSPIYRLEIAMVG-RIFAQDAALY-ADIIAHK--PENLAVIEHLKD
A.aphrophilus_T 259 STFVYGLYLSQQP--VDLEKLLAISSPIYRLEIAMVG-RIFAQDAALY-ADIIADK--PENLAVIEHLKN
N.archeti_TyrA 259 ATFAYGLHLAEEN--VQLEQLLAISSPIYRLEIAMVG-RIFAQDPQLY-ADIIMSS--ENNLALIKRYYQ
Y.kristensenii_ 259 ATFAYGLHLAEEN--VQLEQLLAISSPIYRLEIAMVG-RIFAQDPQLY-ADIIMSS--EDNLALIKRYYK
S.marcescens_Ty 259 ATFAYGLHLAEEN--VQLEQLLAISSPIYRLEIAMVG-RIFAQDPQLY-ADIIMSS--EENVALIKRYYQ
E.coli_TyrA 259 ATFAYGLHLAEEN--VQLEQLLAISSPIYRLEIAMVG-RIFAQDPQLY-ADIIMSS--ERNLALIKRYYK
C.youngae_TyrA 259 ATFAYGLHLAEEN--VQLEQLLAISSPIYRLEIAMVG-RIFAQDPQLY-ADIIMSS--ESNLALIKRYYQ
S.boydii_TyrA 259 ATFAYGLHLAEEN--VQLEQLLAISSPIYRLEIAMVG-RIFAQDPQLY-ADIIMSS--ERNLALIKRYYK
C.freundii_TyrA 259 ATFAYGLHLAEEN--VQLEQLLAISSPIYRLEIAMVG-RIFAQDPQLY-ADIIMSS--ENNLALIKRYYQ
E.fergusonii_Ty 259 ATFAYGLHLAEEN--VQLEQLLAISSPIYRLEIAMVG-RIFAQDPQLY-ADIIMSS--ERNLALIKRYYQ
S.enterica_TyrA 259 ATFAYGLHLAEEN--VQLEQLLAISSPIYRLEIAMVG-RIFAQDPQLY-ADIIMSS--ERNLALIKRYYK
C.sakazakii_Tyr 259 ATFAYGLHLAEEN--VQLEQLLAISSPIYRLEIAMVG-RIFAQDPQLY-ADIIMSS--PGNLALIKRYYQ
X.hominickii_Ty 259 TTFSYGWHLAKED--IDLQQLLSISSPIYRLEIAMVG-RIFAQDSQLY-ADIIMSN--PKNIELIRRYHQ
P.asymbiotica_T 259 TTFVYGKHLAQEG--ADLQQLLSISSPIYRLELIMVG-RIFAQDPQLY-ADIIMSS--PENIDLIRRYHQ
M.fervens_TyrA 185 AFISLGATLKELN--VDIKESRKFASPIYELMISIIG-RIGQNPYLY-ADIQMFN--PRIKEIHETFIN
M.maripaludis_T 184 VYISLGSTLKDLG--IDIKESKNFASPIYELMINIIA-RIGQNPYLY-ADIQMHN--PQIKTIHDTFIK
M.jannaschii_Ty 186 AFISLGATLKELN--VDIKESRKFASPIYELMISIIG-RIGQNPYLY-ADIQMFN--PRIKEIHETFIN
M.vulcani_TyrA 178 AYITIGTTFDALD--FSVNESRRFMSPVYDIMIDFVG-RILGQNPYLY-AYIQMEN--PEVLKVHDAFMN
M.burtonii_TyrA 178 AYITIGNTFKSLD--FDVSMSRRFMSPVYEIMIDFVG-RILGQNPYLY-AHIQMEN--EQVLKVHETFIS
M.thermophila_T 176 SYIAVGSTLRALD--FDVSRSRRFMSPVYEVMVDFVG-RILDQNPELY-ASIQMNP---FAKEVRRVFIE
M.concilii_TyrA 179 AYISIGAALKALD--FDVQRSHRFMSPVYEIMIDFVG-RILDQNPELY-ASIQMNP---KAAAARQAFVS
M.harundinacea_ 188 AYISIGSTLRSLD--FDIAHSRRIMSPVYEIMIDFVG-RILAQSPELY-SSIQENP---EAKRVREAYIE
M.barkeri_TyrA1 197 AYITIGTTIDRLD--FDIKKSRKFVSPVYAIMIDFVG-RILGQNPYLY-ALIQMEN--PGVLEVHDAFIR
M.barkeri_TyrA2 197 AYITIGTTIDRLD--FDIKKSKKFVSPVYDIMIDFVG-RILGQNPYLY-ALIQMEN--TGVLEVHEAFIK
M.horonobensis_ 217 AYITIGTTIDRLD--FDIKKSRKFVSPVYSIMIDFVG-RILGQNPYLY-ALIQMEN--PGVPEVHEAFIK
M.mazei_TyrA 187 AYIAIGTTIDRLD--FDVKKSRKFVSPVYSIMIDFVG-RILGQNPYLY-ALIQMEN--PGVPEVHEAFIK
C.methanoperede 178 AYITIGTVFRELE--FDVAKSRRFMSPMYDIMIDLVG-RILAQNPYLY-AMIQMNP---EVAKVHKAYID
P.homiensis_Tyr 170 IARGLADLGA---------GSSRIGTASYQRLIEAAA-MVQSDSPELL-RTILTEN--PHAAAIRQKFLR
R.pomeroyi_TyrA 195 IAQAMNRMGP---------LPDRMTTASFDLLKQAVE-MVRHDPPGVL-AAIESAN--PFAPRVREEFLG
M.hallyeonensi_ 184 IAKVLLRMGP---------LPTRMTTKSFDLISEAIS-MVQHDAPEVF-EAIEKAN--PYAETVRRRFFD
J.pohangensis_T 184 IAKVLLRMGP---------LPTHMTTKSFDLISEAIS-MVQHDAPEVF-EAIEMAN--PYAETVRRRFFD
R.halocynthiae_ 184 IAKVLLRMGP---------LPTRMTTKSFDLISDAIS-MVQHDAPEVF-EAIETAN--PYAETVRRQFFD
P.ascidiaceicol 184 IAKVLLRMGP---------LPTRMTTKSFDLISEAIS-MVQHDAPEVF-EAIEKAN--PYAETVRRRFFD
O.pituitosum_Ty 184 IAKVLVQMEP---------LPTRMTTKSFDLIMQAVG-MVRHDAPEVF-HAIERAN--DFAPQIRQRFFE
O.anthropi_TyrA 183 IAKVLVQMEP---------LPTRMTTKSFDLLMQAVG-MVRHDAPEVF-QAIEHAN--PYAPQVRRRFFE
O.intermedium_T 191 IARVLVQMEP---------LPTRMTTKSFDLLMQAVS-MVRHDAPEVF-QAIEHAN--PYAPQVRRHFFA
B.canariense_Ty 185 IAKVLVQMEP---------FPARMTTKSFDLLVQATA-MVRDDAPEVF-QAIERSN--PYSQRVRRRFFD
P.inhibens_TyrA 181 IAQALNQVAP---------ETLRMTTASFELLQQASR-MVTGDAHGVL-EAILRDN--PFAADIRDSFLE
P.zucineum_TyrA 176 IGRSLVNLGI---------PDERLATQSYQHLLEICG-LIGADTFELF-KAIQTQN--PYAPKVVAAFVD
S.spMCT13_TyrA 173 IGRTLVNIRI---------PDEELKTNSYQHLLEICG-LIRDDTKELF-FAIQNLN--PFAAEITQQFIA
A.excentricus_T 196 IAKVLSGLEP---------LPRVHTTRSYDLMMQGVG-LVQGDSDELF-MSIERDN--PFASEIRKRFFA
S.cellulosum_Ty 191 IAKGMMDAGVP--------AGAPYAPPSFQGIAHTIE-TVRVDAGHLF-AAIHREN--PFAGEARRRLID
M.xanthus_TyrA 176 LAHGLLKAEAG--------KDLPFAPPSFQPVARIEE-YARIEVPHLF-GVVQSEN--PYARDARVRLLE
C.bacteriumRBG_ 178 VALVAADSLLALG---DFESLESLGGTSCKLLMMAD-SVLSEDPQLY-ASIQMNL--HGMSDIHRLIGE
D.mccartyi_TyrA 178 IAIVSADTLLGQ----NLPGISSAGGTTFRLLDTLTK-SVLTEDPGLY-ASIQVNM--PKLPKLEADFIK
D.mccartyi_TyrA 178 IAIVSADTLLGQ----NLPGIAGAGGTTFRLLDTLTK-SVLTEDPNLY-ASIQVNM--PKLPKLEADFIK
D.spWBC-2_TyrA 178 IAIVTADALVSLD---RLDEMNGAGGITYKALLTLVE-SVLSEDPALY-ASIQINL--PSLPETESLFSD
D.lykanthropore 179 IAIVTADALISLD---KLTEMNSASGITYKALLTLVE-SVLSEDPSLY-ASIQINL--PELPEMEKLFGE
D.alkenigignens 184 IAIVTADALVSLD---RLTEMSGASGITYKALLTLVE-SVLSEDPALY-ASIQINL--PHLPEIESLFAD
A.sulfaticallid 169 LLIAMANFLKDRM----DKNDLNYASPIFYTLYKIAS-RIINQDWRMY-YNIQKN-----SEQLREELVK
A.profundus_Tyr 161 TLIALADFLKDRV----G-EIINYASPIFCVLYKIAS-RIVSQNWKMY-LKIQKN-----AEEKREEFLK
A.veneficus_Tyr 160 LLVGMAHFLAERF----DREDLKYASPIFATLYKISS-RILNQDWRMY-YYIQKN-----AEILRREFIK
G.ahangari_TyrA 160 LLFLFALTLRGRF-----GKAYHYGSPIFLVMHKIAS-RIINQDWRLY-YHIQKN-----AEELRKEILE
F.placidus_TyrA 161 LLLTLSYYLREWF----NEEKLKILSPISLTLIKIAS-RIINQDWKMY-EYILEN-----GREVRKEIIK

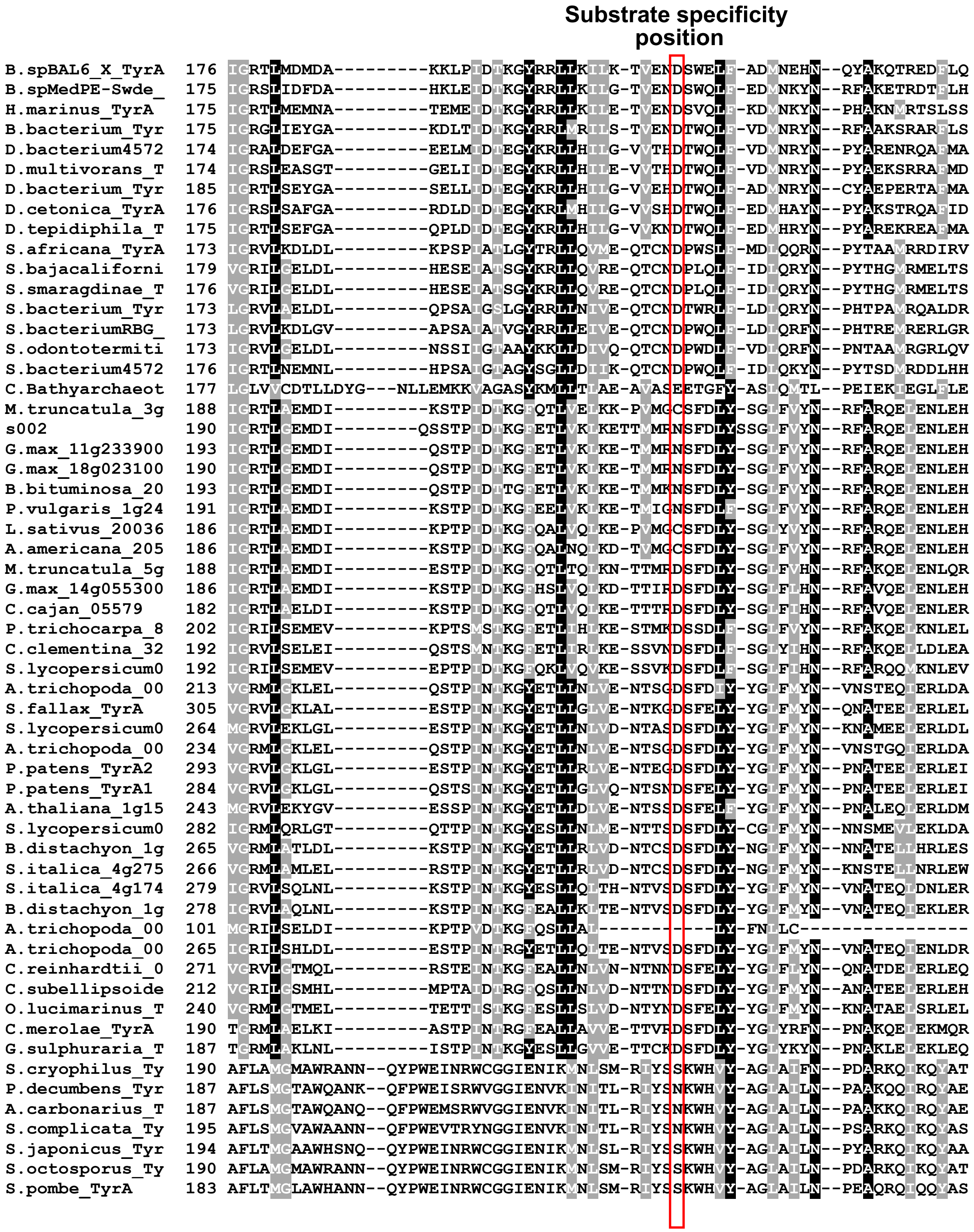
Substrate specificity position
B.spBAL6_X_TyrA 176 IGRTLMDMDA---------KKLPIDTKGYRRLLKILK-TVENDSWELF-ADMNEHN--QYAKQTREDFLQ
B.spMedPE-Swde_ 175 IGRSLIDFDA---------HKLEIDTKGYRRLLKILG-TVENDSWQLF-EDMNKYN--RFAKETRDTFLH
H.marinus_TyrA 175 IGRTLMEMNA---------TEMEIDTKGYRRLLKILE-TVENDSVQLF-KDMNKYN--PHAKNMRTSLSS
B.bacterium_Tyr 175 IGRGLIEYGA---------KDLTIDTKGYRRLMRILS-TVENDTWQLF-VDMNRYN--RFAAKSRARFLS
D.bacterium4572 174 IGRALDEFGA---------EELMIDTEGYKRLLHILG-VVTHDTWQLF-VDMNRYN--PYARENRQAFMA
D.multivorans_T 174 IGRSLEASGT---------GELIIDTEGYKRLLHILE-VVTHDTWQLF-VDMNRYN--PYAEKSRRAFMD
D.bacterium_Tyr 185 IGRTLSEYGA---------SELLIDTEGYKRLLHILG-VVEHDTWQLF-ADMNRYN--CYAEPERTAFMA
D.cetonica_TyrA 176 IGRSLSAFGA---------RDLDIDTEGYKRLMHILG-VVSHDTWQLF-EDMHAYN--PYAKSTRQAFID
D.tepidiphila_T 175 IGRTLSEFGA---------QPLDIDTEGYKRLLHILG-VVKNDTWQLF-EDMHRYN--PYAREKREAFMA
S.africana_TyrA 173 IGRVLKDLDL---------KPSPIATLGYTRLLQVME-QTCNDPWSLF-MDLQQRN--PYTAAMRRDIRV
S.bajacaliforni 179 VGRILGELDL---------HESEIATSGYKRLLQVRE-QTCNDPLQLF-IDLQRYN--PYTHGMRMELTS
S.smaragdinae_T 176 VGRILGELDL---------HESEIATSGYKRLLQVRE-QTCNDPLQLF-IDLQRYN--PYTHGMRMELTS
S.bacterium_Tyr 173 LGRVLAELDL---------QPSAIGSLGYRRLLNIVE-QTCNDTWRLF-LDLQRYN--PHTPAMRQALDR
S.bacteriumRBG_ 173 LGRVLKDLGV---------APSAIATVGYRRLLEIVE-QTCNDPWQLF-LDLQRFN--PHTREMRERLGR
S.odontotermiti 173 IGRVLGELDL---------NSSIIGTAAYKKLLDIVQ-QTCNDPWDLF-VDLQRFN--PNTAAMRGRLQV
S.bacterium4572 176 IGRTLNEMNL---------HPSAIGTAGYSGLLDIIK-QTCNDPWQLF-IDLQKYN--PYTSDMRDDLHH
C.Bathyarchaeot 177 LGLVVCDTLLDYG---NLLEMKKVAGASYKMLLTIAE-AVASEETGFY-ASLQMTL--PEIEKIEGLFLE
M.truncatula_3g 188 IGRTLAEMDI---------KSTPIDTKGFQTLVELKK-PVMGCSFDLY-SGLFVYN--RFARQELENLEH
s002 190 IGRTLGEMDI--------QSSTPIDTKGFETLVKLKETTMMRNSFDLYSSGLFVYN--RFARQELENLEH
G.max_11g233900 193 IGRTLGEMDI---------QSTPIDTKGFETLVKLKE-TMMRNSFDLY-SGLFVYN--RFARQELENLEH
G.max_18g023100 190 IGRTLGEMDI---------QSTPIDTKGFETLVKLKE-TMMRNSFDLY-SGLFVYN--RFARQELENLEH
B.bituminosa_20 193 IGRTLGEMDI---------QSTPIDTTGFETLVKLKE-TMMKNSFDLY-SGLFVYN--RFARQELENLEH
P.vulgaris_1g24 191 IGRTLAEMDI---------KSTPIDTKGFEELVKLKE-TMIGNSFDLF-SGLFVYN--RFARQELENLEH
L.sativus_20036 186 IGRTLAEMDI---------KPTPIDTKGFQALVQLKE-PVMGCSFDLY-SGLYVYN--RFARQELENLEH
A.americana_205 186 IGRTLAEMDI---------KSTPIDTKGFQALNQLKD-TVMGCSFDLY-SGLFVYN--RFARQELENLEH
M.truncatula_5g 188 IGRTLAEMDI---------ESTPIDTKGFQTLTQLKN-TTMRDSFDLY-SGLFVHN--RFAKQELENLQR
G.max_14g055300 186 IGRTLAEMDI---------KSTPIDTKGFHSLVQLKD-TTIRDSFDLY-SGLFLHN--RFAVQELENLEH
C.cajan_05579 182 IGRTLAELDI---------KSTPIDTKGFQTLVQLKE-TTTRDSFDLY-SGLFIHN--RFAVQELENLER
P.trichocarpa_8 202 IGRILSEMEV---------KPTSMSTKGFETLIHLKE-STMKDSSDLF-SGLFVYN--RFAKQELKNLEL
C.clementina_32 192 IGRVLSELEI---------QSTSMNTKGFETLIRLKE-SSVNDSFDLF-SGLYIHN--RFAKQELLDLEA
S.lycopersicum0 192 IGRILSEMEV---------EPTPIDTKGFQKLVQVKE-SSVKDSFDLF-SGLFIHN--RFARQQMKNLEV
A.trichopoda_00 213 VGRMLGKLEL---------QSTPINTKGYETLLNLVE-NTSGDSFDIY-YGLFMYN--VNSTEQIERLDA
S.fallax_TyrA 305 VGRVLGKLAL---------ESTPINTKGYETLLGLVE-NTKGDSFELY-YGLFMYN--QNATEELERLEL
S.lycopersicum0 264 MGRVLEKLGL---------ESTPINTKGYETLLNLVD-NTASDSFDLY-YGLFMYN--KNAMEELERLDL
A.trichopoda_00 234 VGRMLGKLEL---------QSTPINTKGYETLLNLVE-NTSGDSFDLY-YGLFMYN--VNSTGQIERLDA
P.patens_TyrA2 293 VGRVLGKLGL---------ESTPINTKGYETLLRLVE-NTEGDSFDLY-YGLFMYN--PNATEELERLEI
P.patens_TyrA1 284 VGRVLGKLGL---------QSTSINTKGYETLLGLVQ-NTSNDSFELY-YGLFMYN--PNATEELERLEI
A.thaliana_1g15 243 MGRVLEKYGV---------ESSPINTKGYETLLDLVE-NTSSDSFELF-YGLFMYN--PNALEQLERLDM
S.lycopersicum0 282 IGRMLQRLGT---------QTTPINTKGYESLLNLME-NTTSDSFDLY-CGLFMYN--NNSMEVLEKLDA
B.distachyon_1g 265 VGRMLATLDL---------KSTPINTKGYETLLRLVD-NTCSDSFDLY-NGLFMYN--NNATELLHRLES
S.italica_4g275 266 VGRMLAMLEL---------RSTPINTKGYETLLRLVD-NTCSDSFDLY-NGLFMYN--KNSTELLNRLEW
S.italica_4g174 279 IGRVLSQLNL---------KSTPINTKGYESLLQLTH-NTVSDSFDLY-YGLFMYN--VNATEQLDNLER
B.distachyon_1g 278 IGRVLAQLNL---------KSTPINTKGFEALLKLTE-NTVSDSFDLY-YGLFMYN--VNATEQIEKLER
A.trichopoda_00 101 MGRILSELDI---------KPTPVDTKGFQSLLALL-----------LY-FNLLC----------------
A.trichopoda_00 265 IGRILSHLDL---------ESTPINTRGYETLLQLTE-NTVSDSFDLY-YGLFMYN--VNATEQIENLDR
C.reinhardtii_0 271 VGRVLGTMQL---------RSTEINTKGFEALLNLVN-NTNNDSFELY-YGLFLYN--QNATDELERLEQ
C.subellipsoide 212 VGRILGSMHL---------MPTAIDTRGFQSLLNLVD-NTTNDSFDLY-YGLFMYN--ANATEELERLEH
O.lucimarinus_T 240 VGRMLGTMEL---------TETTISTKGFESLLSLVD-NTYNDSFELY-YGLFMYN--KNATAELSRLEL
C.merolae_TyrA 190 TGRMLAELKI---------ASTPINTRGFEALLAVVE-TTVRDSFDLY-YGLYRFN--PNAKQELEKMQR
G.sulphuraria_T 187 TGRMLAKLNL---------ISTPINTKGYESLLGVVE-TTCKDSFDLY-YGLYKYN--PNAKLELEKLEQ
S.cryophilus_Ty 190 AFLAMGMAWRANN--QYPWEINRWCGGIENIKMNLSM-RIYSSKWHVY-AGLAIFN--PDARKQIKQYAT
P.decumbens_Tyr 187 AFLSMGTAWQANK--QFPWEISRWVGGIENVKINITL-RIYSNKWHVY-AGLAILN--PAAKQQIRQYAE
A.carbonarius_T 187 AFLSMGTAWQANQ--QFPWEMSRWVGGIENVKINITL-RIYSNKWHVY-AGLAILN--PAAKKQIRQYAE
S.complicata_Ty 195 AFLSMGVAWAANN--QFPWEVTRYNGGIENVKINLTL-RIYSNKWHVY-AGLAILN--PSARKQIKQYAS
S.japonicus_Tyr 194 AFLTMGAAWHSNQ--QYPWEINRWCGGIENIKMNLSL-RIYSSKWHVY-AGLAILN--PYARKQIKQYAA
S.octosporus_Ty 190 AFLAMGMAWRANN--QYPWEINRWCGGIENIKMNLSM-RIYSSKWHVY-AGLAILN--PDARKQIKQYAT
S.pombe_TyrA 183 AFLTMGLAWHANN--QYPWEINRWCGGIENIKMNLSM-RIYSSKWHVY-AGLAILN--PEAQRQIQQYAS

```
s003               248 QHLDQLITLISNQQ-----------QWPE----LHRL---------LQQTNGDRDKYVE-----------
S.spPCC6803_Tyr    246 QHLDQLITLISN-Q-----------QWPE----LHRL---------LQQTNGDRDKYVE-----------
B.animalis_TyrA    275 -WLSEFANALHEED------------ETE----IRHF---------FAEGQPYRDYKAQI---------N
B.pseudocatenul    251 -RLTLMANVLHGMTTQQGS--PTAGDDKE----MARF---------FIQGQPFREYKVLA---------K
B.adolescentis_    251 -RLTLVANVLHDMADTGVR--PTEGDVKQ----MDRF---------FAQGQPFRDYKAAS---------R
B.dentium_TyrA     251 -RLTLMANVLHGVQPQGAGALQTAATQESDAKEMARF---------FEQGQPFRDYKTAI---------R
S.meliloti_TyrA    257 -DLASLQRAIRWGD------------GDK----LFDL---------FTRTRAIRRSIVQA---------G
R.leguminosarum    257 -DLSY-----------------------------------------------------------------
T.sp_TyrA          249 -VLSDFICYLENDD------------IDS----IQKF---------LEDARKYRNTIT------------
D.invisus_TyrA     248 -SLSAIKTALERAD------------KNT----LHEL---------LEKAGKRKRNLTAA---------D
B.anthracis_Tyr    265 -EMEDLYDTVSSGD------------AGE----IQNY---------FADAKEYRDSLPVR---------K
S.thermophilus_    256 -RLDHVADLIKAED------------ESA----IWEF---------FDNGRKRKEMEIH----------K
A.aeolicus_TyrA    251 -SLNHLKELIVREA------------EEE----LVEY---------LKEVKIKRMEI-------------
R.pneumotropicu    323 -TYENALKFFENHD------------RQG----FIDT---------FHQVREWFGEYSEQ----------
s001               232 -TYDEALTFFENND------------RQG----FIDA---------FHKVRDWFGDYSEQ----------
H.influenzae_Ty    326 -TYEEALAFFENND------------RQG----FIDA---------FHKVRDWFGDYSEQ----------
A.succinogenes_    323 -SYEESLKFFENGD------------KAG----FIEA---------FNQVREWFGEYSEQ----------
H.massiliensis_    323 -SYEESLAFFENGD------------KQG----FIDC---------FNRVREWFGEYSQQ----------
H.somni_TyrA       323 -SYEESLNFFEHNN------------KQG----FIDC---------FNQVREWFGEYSEQ----------
A.actinomycetem    323 -SYETGLAFFKHHD------------RQG----FIEQ---------FNQIRDWFGGYSEQ----------
A.aphrophilus_T    323 -SYETGFAFFKNKD------------KAG----FIAQ---------FNQIRDWFGEYSEQ----------
N.archeti_TyrA     323 -RFGEAIALLEQGD------------KQA----FIDS---------FKKVEHWFGDYAQR----------
Y.kristensenii_    323 -RFGEAITLLEQSD------------KQA----FVKS---------FQKVEHWFGDYAER----------
S.marcescens_Ty    323 -RFGEAIKLLEHGD------------KQA----FIDS---------FRKVEHWFGDYAQR----------
E.coli_TyrA        323 -RFGEAIELLEQGD------------KQA----FIDS---------FRKVEHWFGDYAQR----------
C.youngae_TyrA     323 -RFGEAIGLLEQGD------------KQA----FIDS---------FRKVEHWFGDYAKR----------
S.boydii_TyrA      323 -RFGEAIELLKQGD------------KQA----FIDS---------FRKVEHWFGDYAQR----------
C.freundii_TyrA    323 -RFGEAIGLLEQGD------------KQA----FIDS---------FRKVEHWFGDYAKR----------
E.fergusonii_Ty    323 -RFGEAIELLEQGD------------KQA----FIDS---------FRKVEHWFGDYAQR----------
S.enterica_TyrA    323 -RFGDAIGLLEQGD------------KQA----FIDS---------FRKVEHWFGDYARR----------
C.sakazakii_Tyr    323 -RFGEAIGLLEQGD------------KRA----FIDS---------FRKVEHWFGDYAKR----------
X.hominickii_Ty    323 -SFGQALKLLENQD------------KSA----FIAS---------FNQVREWFGDEATR----------
P.asymbiotica_T    323 -SFGQALEILESRD------------KRA----FVSS---------FEDVSAWFGDYAPH----------
M.fervens_TyrA     249 -QCKEISKIVKNKD------------REG----FVKI---------MKEAAKHFGSEAKR----------
M.maripaludis_T    248 -NCENISEIVQNKD------------RDS----FVEN---------MKNSAKHFGNETKR----------
M.jannaschii_Ty    250 -QCKEISEIVKNKD------------REG----FVKI---------MKEAAKHFGSEAKR----------
M.vulcani_TyrA     242 -QCSEMSSIVRKKD------------VEA----FTNK---------MKEAAVHFGDTASA----------
M.burtonii_TyrA    242 -ECNILSEIVREQD------------IEA----FCNK---------MTEAAAHFKDTPSA----------
M.thermophila_T    239 -ECLRLSDAVDHGD------------LEG----FMRT---------MREAADHFGDTHSA----------
M.concilii_TyrA    242 -ECMRLCEKTDDGD------------MEG----FKQM---------MREAALHYGGTHEA----------
M.harundinacea_    251 -ECQRLSKLADTGN------------SEG----FMEA---------MRSAADHFDEREVA----------
M.barkeri_TyrA1    261 -ECEELSRLVRAHD------------EES----FVKK---------MKSAARKYGDTAHA----------
M.barkeri_TyrA2    261 -ECEELSRLVRAHD------------EES----FVKK---------MKAAARKYGDTAHA----------
M.horonobensis_    281 -ECEELAGLVRAHD------------EEG----FVRK---------MKAAARKYDDTAHA----------
M.mazei_TyrA       251 -ECEELSSLVKAHD------------EEG----FVRK---------MKAAARKYDDTSHA----------
C.methanoperede    241 -QCNLIAEMVQRKD------------IEG----FVSL---------MKKAAAHFGDTESA----------
P.homiensis_Tyr    227 -GAAGAAI--------------------------------------------------------------
R.pomeroyi_TyrA    252 -LIEGLDRAYAGRD--------------------------------------------------------
M.hallyeonensi_    241 -LAASLSVELEATS------------TTQ----PHNE-------------SGH-----------------
J.pohangensis_T    241 -LAAGLSVELDTAS------------ATP----LLQE-------------RGH-----------------
R.halocynthiae_    241 -LAASLSVELENSI------------KDT----AVQ----------------------------------
P.ascidiaceicol    241 -LAASLSEELEAAL------------RAS----PNNE-------------SGH-----------------
O.pituitosum_Ty    241 -LAANLNEELSRVS------------QA------------------------------------------
O.anthropi_TyrA    240 -LADRVNAELARPD------------PFI----AAEE-------------EATGAFRKSA----------
O.intermedium_T    248 -LADQINEELARVQ------------PMA----AREE-------------AA------------------
B.canariense_Ty    242 -LASALDAELGNRS------------PVR----LSAD-------------RSSVKFDTSQ---------T
P.inhibens_TyrA    238 -RAADLGRHPVTPR------------TQQ----SPPN-------------AAGF----------------
P.zucineum_TyrA    233 -EAKSLLEQVRAEG------------A-------------------------------------------
S.spMCT13_TyrA     230 -EANGLLAESLAAG------------KAA-----------------------------------------
A.excentricus_T    253 -EIDSLRERLEAHG------------RET----D------------------------------------
S.cellulosum_Ty    249 -ALVAVDRSLDVPT------------KDA----APED---------LAALAIPDLGALSP----------
M.xanthus_TyrA     234 -ALTQLHLGCDSGR------------SGA----PEPVAAPPGLTEVRECVDALDRELV------------
C.bacteriumRBG_    241 -KLTEWAQLVRNGD------------RQG----FIAR---------MEALKKQRAQAGPD----------
D.mccartyi_TyrA    240 -KATEWAELVKNGD------------KTK----FARR---------MQTLKDNLARTEPG----------
D.mccartyi_TyrA    240 -RATQWAELVKNGN------------KAG----FAHR---------MQTLKDNLARTEPG----------
D.spWBC-2_TyrA     241 -KARDWATLVREGR------------RDE----FVNR---------MSNLKAKLEASNPD----------
D.lykanthropore    242 -KADEWAGLVADGR------------RQD----FIDR---------MSRLKTTLEAANPD----------
D.alkenigignens    247 -KAEYWASLVKDGR------------RDE----FARR---------MAALKSKLETANPD----------
A.sulfaticallid    228 -SILELHDGLRD--------------DAR----FKEI---------FEASKKVFDDFGGS---------T
A.profundus_Tyr    219 -HMISLSERLKD--------------ERE----FKKL---------FEDLRDVYIDKGES----------
A.veneficus_Tyr    219 -SLADLDAKFSD--------------EEK----FRKL---------FEELGSKFDDYGNS----------
G.ahangari_TyrA    218 -NARVLDEIMKD--------------ERS----FKGL---------VDELREEFDDYRDS----------
F.placidus_TyrA    220 -AASEVD-RFIE--------------EGK----VQEL---------FDYLRGAFKNGKNT----------
```

```
B.spBAL6_X_TyrA  233 -SFNKVIKRLED----------------------------------------------------------
B.spMedPE-Swde_  232 -SLQGVLSRLES----------------------------------------------------------
H.marinus_TyrA   232 -ALEETLASVQS----------------------------------------------------------
B.bacterium_Tyr  232 -ALTNIDQKVQAP---------------------------------------------------------
D.bacterium4572  231 -GMKRLEKKLER----------------------------------------------------------
D.multivorans_T  231 -AMKTIEERLKKDR------------LKT----EG-----------------------------------
D.bacterium_Tyr  242 -AMKIVNERLKE----------------------------------------------------------
D.cetonica_TyrA  233 -AMIGIHERLDG----------------------------------------------------------
D.tepidiphila_T  232 -AMARIHDHLKEKE------------DQG----PNLM---------G-----------------------
S.africana_TyrA  230 -SLDRTLQLLGDSD------------AGG----DAAANAGPGADSGAASSD-------------------
S.bajacaliforni  236 -ALEQIMTLFAQAD------------LPP----DRP----------------------------------
S.smaragdinae_T  233 -ALEQIMTLFAQAD------------LPP----DRP----------------------------------
S.bacterium_Tyr  230 -ALEALKAKLDML---------------------------------------------------------
S.bacteriumRBG_  230 -SLDAVMAAIDGGA------------RTN----AGGP---------GT----------------------
S.odontotermiti  230 -AINLMLDKFESIE------------LPE----DI-----------------------------------
S.bacterium4572  233 -GLEKMLNTFDSIE------------SLG----DLDG---------------------------------
C.Bathyarchaeot  240 -KSGEWLDIVKRRD------------RLA----FANK---------MELVKRRLRKVDPD----------
M.truncatula_3g  245 -ALHKVKEMLVQRI------------DEG----QNPE---------RTES--------------------
s002             250 -ALHKVKETLMI----------------------------------------------------------
G.max_11g233900  250 -AFYKVKETLMIQR-----------SNGE----QGHK---------RTES--------------------
G.max_18g023100  247 -ALHKVKETLMIQR-----------TNGE----QGHK---------RTES--------------------
B.bituminosa_20  250 -ALRKVKETLMIQS-----------IDEE----QDKK---------ITES--------------------
P.vulgaris_1g24  248 -ALQKVKETLVERK------------NEE----QGQE---------KN----------------------
L.sativus_20036  243 -ALHKVKEMLVQSM------------DEG----QNSE---------RTES--------------------
A.americana_205  243 -ALHKVKEMLVPRI------------NEE----HGPE---------RTES--------------------
M.truncatula_5g  245 -ALDRVKEMLVQRM------------REE----LGPE---------KD----------------------
G.max_14g055300  243 -ALHKVKEMLVQRK------------SEE----LGLE---------KTEC--------------------
C.cajan_05579    239 -ALQRVKEMLAQRM------------NEE----LGTE---------------------------------
P.trichocarpa_8  259 -SLEKVKQMLQDKM------------TEE----QNLN---------ESKF--------------------
C.clementina_32  249 -AFEKVKHKLQQKME---------EVQLE----QSPN---------ESKL--------------------
S.lycopersicum0  249 -ALEKTKEKLQERS------------KEL----QDPI---------ISKF--------------------
A.trichopoda_00  270 -AFDSLKRQLYGHL------------HDV----LRKQ---------LFGTGKGSENLHRL---------A
S.fallax_TyrA    362 -AFDSIKKQLFGRL------------HDV----LRQQ---------LFE---GVGMKDRI---------S
S.lycopersicum0  321 -AFEALKKELFGHL------------HDL----LRKQ---------LFG--KAEEAGQRR---------V
A.trichopoda_00  291 -AFDSLKRQLYGHL------------HDI----LRKQ---------LFGNGKGSENLQRL---------A
P.patens_TyrA2   350 -AFDSIKRQLFGQL------------HDV----LRKQ---------LFG---ANVADKAR---------L
P.patens_TyrA1   341 -AFHSVKRQLFGQL------------HDR----FRKQ---------LFSDSSAPRASIPQ---------D
A.thaliana_1g15  300 -AFESVKKELFGRL------------HQQ----YRKQ---------MFG---GEVQSPKK---------T
S.lycopersicum0  339 -ALDSLKRELFGQV------------LQK----LEKR----------------VEKGSRL---------A
B.distachyon_1g  322 -AMDSVKRRLFDGL------------HEV----LRRQ---------LFEGSPPLNRDSSF---------P
S.italica_4g275  323 -AMDSVKKRLFDGL------------HDV----LRRQL-------FDFEGSPAD---------------P
S.italica_4g174  336 -AFEKVRQMLYGRL------------HDL----LRKQ---------IVERVPMPVVSSRK----------
B.distachyon_1g  335 -AFEKVKQMLYGRL------------HDI----LRKQ---------IVERVPI-----------------
A.trichopoda_00  134 ----GVTSLFWFAL------------AER-----------------------------------------
A.trichopoda_00  322 -AFETVKQKLFGRL------------HDI----LRKQ---------IIERVPHQGVPSIRDTTKTPYFLP
C.reinhardtii_0  328 -AFDTVKKQLFGRL------------HDI----ARQQ---------LFPTVDSQGNGNGA---------A
C.subellipsoide  269 -AFDSVKKQLFRRL------------HDV----AREQ---------LFQQAPA----------------A
O.lucimarinus_T  297 -AFTKVKGELFDRL------------HER----IRND---------IFEES-------------------
C.merolae_TyrA   247 -ALEQVRAQLEAYD------------RKC----REAE---------ARSA--------------------
G.sulphuraria_T  244 -ALQALRKELETRE-------------------ALDG---------WKKD--------------------
S.cryophilus_Ty  254 -SVTDLFKLAISGK------------AQE----FESR---------VRTAGKFVFGEQFT---------A
P.decumbens_Tyr  251 -SVTELYKLMIGGH------------REE----LKRR---------VKAAGASVFKEGTA----------
A.carbonarius_T  251 -SVTDLYKLMIEGR------------RDE----LKSR---------VRAAGKAVFREGTN----------
S.complicata_Ty  259 -SVTELYKLIIERR------------AEE----FTNR---------IHEAARFVFGSPHSI------NPL
S.japonicus_Tyr  258 -SVTDLYKLAVTGR------------NEE----FERR---------VRAAGIAVFGEHRD---------H
S.octosporus_Ty  254 -SVTELFKLSISGK------------AQE----FENR---------VRNAGKFVFGEQNN---------G
S.pombe_TyrA     247 -SVTELFKLAISGK------------AKE----YEDR---------IRNAGKFVFGENMD---------R
```

```
s003                     ----------------------------------------------------------------------
S.spPCC6803_Tyr          ----------------------------------------------------------------------
B.animalis_TyrA     310  AGKVSDSTE---TIFQSLRID-PEHWREQLLKSAQR-------GEYIVRFTSGHR---------------
B.pseudocatenul     296  EPDFEERCE---TVELAIPE--TGWQ-QMLLESARR-------GEHIVRFDGYRQ---------------
B.adolescentis_     296  QPEYAEHCA---TVELSIPE--NDWQ-RTLLESARR-------GEHIVRFDGERT---------------
B.dentium_TyrA      302  QPDFMERCE---TVSLAIPA--EGWQ-QMLLESARR-------GEHIIRFTDDHA---------------
S.meliloti_TyrA     292  QDTAMP---------------------DFGRHAMDQK---------------------------------
R.leguminosarum          ----------------------------------------------------------------------
T.sp_TyrA                ----------------------------------------------------------------------
D.invisus_TyrA      283  KI--------------------------------------------------------------------
B.anthracis_Tyr     300  RGAIPAYHD---LYVDVLDK--VGAL-AHVTSILAR------EEISITNLQILE-AREGLLGVLRISFQR
S.thermophilus_     291  KGGVESAFD---IFVDVPDR--EDVI-LSIMELLRG--------TSLVNIRINEENREDIHGILQITFKN
A.aeolicus_TyrA          ----------------------------------------------------------------------
R.pneumotropicu     357  ---------------FLK-------ESHQLLQQAND------LKQG------------------------
s001                266  ---------------FLK-------ESRQLLQQAY-----------------------------------
H.influenzae_Ty     360  ---------------FLK-------ESRQLLQQAND------LKQG------------------------
A.succinogenes_     357  ---------------FMK-------ESRQLLQQAND------YRQHD-----------------------
H.massiliensis_     357  ---------------FLT-------ESRQLLQQAND------NRRH------------------------
H.somni_TyrA        357  ---------------FLK-------ESRQLLQQAND------YRSI------------------------
A.actinomycetem     357  ---------------FLQ-------ESRQLLQQAND------SRNV------------------------
A.aphrophilus_T     357  ---------------FLQ-------ESRQLLQQASD------ARNV------------------------
N.archeti_TyrA      357  ---------------FLV-------ESRTLLLQAND------NRQ-------------------------
Y.kristensenii_     357  ---------------FLV-------ESRSLLRQAND------SRQ-------------------------
S.marcescens_Ty     357  ---------------FLV-------ESRTLLRQAND------SRQ-------------------------
E.coli_TyrA         357  ---------------FQS-------ESRVLLRQAND------NRQ-------------------------
C.youngae_TyrA      357  ---------------FQS-------ESRTLLRQAND------SRP-------------------------
S.boydii_TyrA       357  ---------------FQS-------ESRVLLRQAND------NRQ-------------------------
C.freundii_TyrA     357  ---------------FQS-------ESRTLLRQAND------SRP-------------------------
E.fergusonii_Ty     357  ---------------FQN-------ESRVLLRQAND------SRQ-------------------------
S.enterica_TyrA     357  ---------------FQN-------ESRVLLRQAND------SRP-------------------------
C.sakazakii_Tyr     357  ---------------FQQ-------ESRALLRQAND------SRQ-------------------------
X.hominickii_Ty     357  ---------------FMK-------ESRVLLQQASD------SRI-------------------------
P.asymbiotica_T     357  ---------------FMR-------ESRVLLQQAND------SRQ-------------------------
M.fervens_TyrA      283  ---------------GAY------YSDKAIFALASE------IEKLNKLIGREIAVKNINSNVIHFGILK
M.maripaludis_T     282  ---------------GLN------CSNKAVYAISKE------TEKLLKSIGKEIGLKHIYSENVHYGILK
M.jannaschii_Ty     284  ---------------GAY------YSDKAVFALTSE------IEKLNKLIGKDVAVKNINSNVVHFGVLK
M.vulcani_TyrA      276  ---------------LRR-------SDKLINSKIAE------FDRLLDSIGEEIGVRHIYSGIVHTGIIK
M.burtonii_TyrA     276  ---------------LHR-------SDKLINAKISE------FEEMIASVGVERGLYHNYSGVTHVGTIK
M.thermophila_T     273  ---------------LQR-------SDRIINQRAQE------RE--------------------------
M.concilii_TyrA     276  ---------------LQR-------SDRVINSRIRE------KEEEGKR---------------------
M.harundinacea_     285  ---------------LAR-------SDRLISLWIDH------REGKRRGLEPRW----------------
M.barkeri_TyrA1     295  ---------------LRK-------SDKLINSRITE------YETILNSVGKVCGFSHFYSGKIHVGILG
M.barkeri_TyrA2     295  ---------------LRK-------SDKLINSRITE------YETILNSVGKVCGFFHIYSGKIHVGTLE
M.horonobensis_     315  ---------------LRR-------SDKLINSRITE------YETILNSIGKVCGFSHIYSGNIHVGRLE
M.mazei_TyrA        285  ---------------LRR-------SDKLINSRIIE------YETILNSTGKVCGFSHIYSGNIHVGRLE
C.methanoperede     275  ---------------LRR-------SDKLIGTKIAE------HEELVRSTGSERAIKHIYSGVIHSGIIR
P.homiensis_Tyr          ----------------------------------------------------------------------
R.pomeroyi_TyrA          ----------------------------------------------------------------------
M.hallyeonensi_          ----------------------------------------------------------------------
J.pohangensis_T          ----------------------------------------------------------------------
R.halocynthiae_          ----------------------------------------------------------------------
P.ascidiaceicol          ----------------------------------------------------------------------
O.pituitosum_Ty          ----------------------------------------------------------------------
O.anthropi_TyrA          ----------------------------------------------------------------------
O.intermedium_T          ----------------------------------------------------------------------
B.canariense_Ty     273  KQTD------------------------------------------------------------------
P.inhibens_TyrA          ----------------------------------------------------------------------
P.zucineum_TyrA          ----------------------------------------------------------------------
S.spMCT13_TyrA           ----------------------------------------------------------------------
A.excentricus_T          ----------------------------------------------------------------------
S.cellulosum_Ty     283  -------------ELRETREL-IDDLDLELVGLLAR------RAELVQRAGRAKATIGAAVRDPVREARL
M.xanthus_TyrA      275  -------------------------------QLLNR------RAQLIQQAAHLKAEHGLPLPDAEREASL
C.bacteriumRBG_     275  ---------------FRK-------AYENMYRILGK----------------------------------
D.mccartyi_TyrA     274  ---------------FEK-------AYQAMYHLNQPR---------------------------------
D.mccartyi_TyrA     274  ---------------FEK-------AYQDMYHLKQP----------------------------------
D.spWBC-2_TyrA      275  ---------------FGA-------SYQKLYHLANHR---------------------------------
D.lykanthropore     276  ---------------FGA-------SYQKLYHLANR------RQD-------------------------
D.alkenigignens     281  ---------------FGV-------SYQALYRLTDRG---------------------------------
A.sulfaticallid     261  IILDSARASEIPERDIHALRGYIRVVDSLILRLIER------RVRAGKEIAMYKKEKNLPIEISEIEEVK
A.profundus_Tyr     251  TIILDSYKATLDVNDLNLVRGYIRALDSLILRLIER------RVETGKKVAEIKKERNEPIEITDVEEIK
A.veneficus_Tyr     251  TLILDACKATWDVSTIELLRGYIRAVDSLILRLLER------RTQAGRKIALHKRDRNEPIEIADLEEVK
G.ahangari_TyrA     250  TIVLDAYKTTVNAEGIDMLRGYIRAIDSLVLRLIEK------RVEAGRKVAMEKLKLNEPVEISHVEDVK
F.placidus_TyrA     251  TIILDSARTTLTPNSLEELRGYIKTVDSLILRLIEK------RVDAGRRIALEKMKRNEPIEVSEVEYFK
```

```
B.spBAL6_X_TyrA      ----------------------------------------------------------------------
B.spMedPE-Swde_      ----------------------------------------------------------------------
H.marinus_TyrA       ----------------------------------------------------------------------
B.bacterium_Tyr      ----------------------------------------------------------------------
D.bacterium4572      ----------------------------------------------------------------------
D.multivorans_T      ----------------------------------------------------------------------
D.bacterium_Tyr      ----------------------------------------------------------------------
D.cetonica_TyrA      ----------------------------------------------------------------------
D.tepidiphila_T      ----------------------------------------------------------------------
S.africana_TyrA      ----------------------------------------------------------------------
S.bajacaliforni      ----------------------------------------------------------------------
S.smaragdinae_T      ----------------------------------------------------------------------
S.bacterium_Tyr      ----------------------------------------------------------------------
S.bacteriumRBG_      ----------------------------------------------------------------------
S.odontotermiti      ----------------------------------------------------------------------
S.bacterium4572      ----------------------------------------------------------------------
C.Bathyarchaeot  274 ---------------YAK-------SYEVMHRLLEA------VKS-------------------------
M.truncatula_3g      ----------------------------------------------------------------------
s002                 ----------------------------------------------------------------------
G.max_11g233900      ----------------------------------------------------------------------
G.max_18g023100      ----------------------------------------------------------------------
B.bituminosa_20      ----------------------------------------------------------------------
P.vulgaris_1g24      ----------------------------------------------------------------------
L.sativus_20036      ----------------------------------------------------------------------
A.americana_205      ----------------------------------------------------------------------
M.truncatula_5g      ----------------------------------------------------------------------
G.max_14g055300      ----------------------------------------------------------------------
C.cajan_05579        ----------------------------------------------------------------------
P.trichocarpa_8      ----------------------------------------------------------------------
C.clementina_32      ----------------------------------------------------------------------
S.lycopersicum0      ----------------------------------------------------------------------
A.trichopoda_00  305 PPALSQWV----CTGSHGM---------------------------------------------------
S.fallax_TyrA    394 LPNIVEDA----AHKSTD-----FATAASN------------VVAHSKATPDRSSKRSGDNNGSS-----
S.lycopersicum0  354 LSKLPRNGY---ALPAPSSD---AVKPENN----------------------------------------
A.trichopoda_00  326 PPAVSRNGY---GQEATACE---VKQ--------------------------------------------
P.patens_TyrA2   382 PHDPSNSS----STNG--------------ISKNGGS----GIASNSNTALPILESVLGNSNELDKDSDS
P.patens_TyrA1   376 SADLSKNG----SSQPKAPSN-NVSRVYNNTDIISSSNGISRIAKSTDTSTSSIELELRKTIGLNKDSYS
A.thaliana_1g15  332 EQKLLNDG----GVVP--------------MNDISSS----SSSSSSSS---------------------
S.lycopersicum0  367 LP---------------TPD---FSKKIEK------------LKVERKELEALS----------------
B.distachyon_1g  357 AESSLDD----------------------------------------------GR---------------
S.italica_4g275  354 AELLVVGG----DLYTDG-------DEGDA------------DAVDG---GSRGE---------------
S.italica_4g174  370 -----LEN-----GRS--------------------------NSFAG---LSYRL---------------
B.distachyon_1g      ----------------------------------------------------------------------
A.trichopoda_00      ----------------------------------------------------------------------
A.trichopoda_00  366 KREEMRDL----SSFAMP-------PQKEE------------LPQKT---EVLGR---------------
C.reinhardtii_0  363 APPPQALPPP-REPLA-LPS---RSRDEVMAQAPSD------AYSASKTIDV------------------
C.subellipsoide  297 APERLLLKAG-RSPQA-EAG---QNSSNGGSQSQRE------HAESTPLI--------------------
O.lucimarinus_T      ----------------------------------------------------------------------
C.merolae_TyrA       ----------------------------------------------------------------------
G.sulphuraria_T      ----------------------------------------------------------------------
S.cryophilus_Ty  289 SSSGLL------------------LSDELLDQYSIS------NVPNDKSVGNSHLSILAIVDSWWKLGIH
P.decumbens_Tyr  285 -SQDLL------------------LKDEVLDQFSLS------NRSREKAPPNSHLSLLAIVDCWSKLGIV
A.carbonarius_T  285 -GQDLL------------------LKDEVLDRFSLG------NRAREESPPNSHLSLLAIVDCWSKLGIV
S.complicata_Ty  297 TSDELL------------------LRDEVLDRFSLG------SVPKDERTPNSHLSLLAIVDCWWKLRIV
S.japonicus_Tyr  293 NYSGLL------------------LSDDLLDQYSIS------NVPNDKTVPNSHLSILAIVDSWAKLGIK
S.octosporus_Ty  289 ASSGLL------------------LSDELLDQYSIS------NVPNDKSVGNSHLSILAIVDSWWKLGIH
S.pombe_TyrA     282 NSSGLL------------------LSDELLDQYSIS------NIPKDESKRNSHLSILAIVDSWSKLGIH
```

```
s003                      ----------------------------------------------------------------------
S.spPCC6803_Tyr           ----------------------------------------------------------------------
B.animalis_TyrA    354    --LIAEQHPSIRS---------------------------------------------------------
B.pseudocatenul    338    --AMAQVRSAV-----------------------------------------------------------
B.adolescentis_    338    --AIAQARSVV-----------------------------------------------------------
B.dentium_TyrA     344    --VDVQIRSAV-----------------------------------------------------------
S.meliloti_TyrA           ----------------------------------------------------------------------
R.leguminosarum           ----------------------------------------------------------------------
T.sp_TyrA                 ----------------------------------------------------------------------
D.invisus_TyrA            ----------------------------------------------------------------------
B.anthracis_Tyr    357    EEDRMKAKLALGEEKYQTYETI------------------------------------------------
S.thermophilus_    347    EKDRKHARTVIEANTDYHVVIA------------------------------------------------
A.aeolicus_TyrA           ----------------------------------------------------------------------
R.pneumotropicu           ----------------------------------------------------------------------
s001                      ----------------------------------------------------------------------
H.influenzae_Ty           ----------------------------------------------------------------------
A.succinogenes_           ----------------------------------------------------------------------
H.massiliensis_           ----------------------------------------------------------------------
H.somni_TyrA              ----------------------------------------------------------------------
A.actinomycetem           ----------------------------------------------------------------------
A.aphrophilus_T           ----------------------------------------------------------------------
N.archeti_TyrA            ----------------------------------------------------------------------
Y.kristensenii_           ----------------------------------------------------------------------
S.marcescens_Ty           ----------------------------------------------------------------------
E.coli_TyrA               ----------------------------------------------------------------------
C.youngae_TyrA            ----------------------------------------------------------------------
S.boydii_TyrA             ----------------------------------------------------------------------
C.freundii_TyrA           ----------------------------------------------------------------------
E.fergusonii_Ty           ----------------------------------------------------------------------
S.enterica_TyrA           ----------------------------------------------------------------------
C.sakazakii_Tyr           ----------------------------------------------------------------------
X.hominickii_Ty           ----------------------------------------------------------------------
P.asymbiotica_T           ----------------------------------------------------------------------
M.fervens_TyrA     326    DIENDYLILDKNGKEQRFNILKVEVFFGDELNELKKKYLERKYIDISILFKKDVDENVILNLLKKIFDIE
M.maripaludis_T    325    EIYSDNIVLKQNEKEVILNISNVSLMENKELEDWKIENLEKYFVDISVLFKKDIDLSIILELLTHKFEIE
M.jannaschii_Ty    327    DIEDDYLILNKNGKEQKFNILRVEVFAGDELSKLKKKHLEKKYIDVSVLFKKDVDEEVILNLLKKMFDIE
M.vulcani_TyrA     318    KVSPRDVVLECGSRVVPLKIENIRLLDEEELYEWKLGNLEHHLRDISVLIPEEADADVIMELVSCNENIV
M.burtonii_TyrA    318    KVTPRAVIISKGGKDVYLKTENIRLYTEKELKDWKVKNLLHPKRDISVLLPSGADASVFEKVIDNDERIA
M.thermophila_T           ----------------------------------------------------------------------
M.concilii_TyrA           ----------------------------------------------------------------------
M.harundinacea_           ----------------------------------------------------------------------
M.barkeri_TyrA1    337    KAGPDEIVLTKMVSKGTSPHIKNKFVKLKLENLRMLSESELWEWRKENLEHSTRDISVLIPEGADPEVIL
M.barkeri_TyrA2    337    KAGLDEIVLTKLVSKGTSLHIKNKFVKLKLENLRMLSESELWKWRKENLEHSTRDISVLIPKGADPVVIL
M.horonobensis_    357    KVGPNEIVLTKLVSKGTAPHIKNKFIKLKLENLRLLSESELREWRKEKLKHSIRDISVLIPEGADPEIVL
M.mazei_TyrA       327    KVRPNEIVLMKLVSKGTAPNIKNRFITLKLENLRPLSEAELREWRKENLEHSVRDISVVIPEGADPEAVL
C.methanoperede    317    KVTPRTVILERGGKKIEFLIENIRLLNELELYAWKSANLINMTRDVSVYIPAASDAEIIKSVIECSGGII
P.homiensis_Tyr           ----------------------------------------------------------------------
R.pomeroyi_TyrA           ----------------------------------------------------------------------
M.hallyeonensi_           ----------------------------------------------------------------------
J.pohangensis_T           ----------------------------------------------------------------------
R.halocynthiae_           ----------------------------------------------------------------------
P.ascidiaceicol           ----------------------------------------------------------------------
O.pituitosum_Ty           ----------------------------------------------------------------------
O.anthropi_TyrA           ----------------------------------------------------------------------
O.intermedium_T           ----------------------------------------------------------------------
B.canariense_Ty           ----------------------------------------------------------------------
P.inhibens_TyrA           ----------------------------------------------------------------------
P.zucineum_TyrA           ----------------------------------------------------------------------
S.spMCT13_TyrA            ----------------------------------------------------------------------
A.excentricus_T           ----------------------------------------------------------------------
S.cellulosum_Ty    333    MESRRRCALELGLDPDGVAEVFEVILRHSRSLQERRSAV-------------------------------
M.xanthus_TyrA     308    LETRRQWAAEQGMDADDTEDVFRAVLRFSRRAVPTGSR--------------------------------
C.bacteriumRBG_           ----------------------------------------------------------------------
D.mccartyi_TyrA           ----------------------------------------------------------------------
D.mccartyi_TyrA           ----------------------------------------------------------------------
D.spWBC-2_TyrA            ----------------------------------------------------------------------
D.lykanthropore           ----------------------------------------------------------------------
D.alkenigignens           ----------------------------------------------------------------------
A.sulfaticallid    325    LKELASSTTLNRMMLNRIFGEIFHLTKAEEYRILGISKRMAVLGPMGSFSDEVALKLTGSRVPFIYCSSV
A.profundus_Tyr    315    IRDLIANTHLNPVLIYRIFDSLMTLTKEEEYRVLGVNKKLAILGPMGSFSEEMALRLVGSRLPLIYCSTT
A.veneficus_Tyr    315    IKDLLLRTSLNPIYVQDIFENIMGLTKEEEYRVLGIKKTLAVLGPAGSFSEEAALKLVGSRLPLKYCATT
G.ahangari_TyrA    314    LRELVTKTELNPVMVSEIFERLMKLTKEEEYRILGVRKKVAVLGPPGSFSEETALKLVGSRLPLIYQSKV
F.placidus_TyrA    315    LKELTSKTSLDAGSVSSIFEEIMSLTKAEEYKVAGVEKKVAVLGPAGSFSEEAALKLIDSKLPLIYCSSV
```

```
B.spBAL6_X_TyrA          ----------------------------------------------------------------------
B.spMedPE-Swde_          ----------------------------------------------------------------------
H.marinus_TyrA           ----------------------------------------------------------------------
B.bacterium_Tyr          ----------------------------------------------------------------------
D.bacterium4572          ----------------------------------------------------------------------
D.multivorans_T          ----------------------------------------------------------------------
D.bacterium_Tyr          ----------------------------------------------------------------------
D.cetonica_TyrA          ----------------------------------------------------------------------
D.tepidiphila_T          ----------------------------------------------------------------------
S.africana_TyrA          ----------------------------------------------------------------------
S.bajacaliforni          ----------------------------------------------------------------------
S.smaragdinae_T          ----------------------------------------------------------------------
S.bacterium_Tyr          ----------------------------------------------------------------------
S.bacteriumRBG_          ----------------------------------------------------------------------
S.odontotermiti          ----------------------------------------------------------------------
S.bacterium4572          ----------------------------------------------------------------------
C.Bathyarchaeot          ----------------------------------------------------------------------
M.truncatula_3g          ----------------------------------------------------------------------
s002                     ----------------------------------------------------------------------
G.max_11g233900          ----------------------------------------------------------------------
G.max_18g023100          ----------------------------------------------------------------------
B.bituminosa_20          ----------------------------------------------------------------------
P.vulgaris_1g24          ----------------------------------------------------------------------
L.sativus_20036          ----------------------------------------------------------------------
A.americana_205          ----------------------------------------------------------------------
M.truncatula_5g          ----------------------------------------------------------------------
G.max_14g055300          ----------------------------------------------------------------------
C.cajan_05579            ----------------------------------------------------------------------
P.trichocarpa_8          ----------------------------------------------------------------------
C.clementina_32          ----------------------------------------------------------------------
S.lycopersicum0          ----------------------------------------------------------------------
A.trichopoda_00          ----------------------------------------------------------------------
S.fallax_TyrA            ----------------------------------------------------------------------
S.lycopersicum0          ----------------------------------------------------------------------
A.trichopoda_00          ----------------------------------------------------------------------
P.patens_TyrA2       430 ISKSSKQ---------------------------------------------------------------
P.patens_TyrA1       441 VSTVLK----------------------------------------------------------------
A.thaliana_1g15          ----------------------------------------------------------------------
S.lycopersicum0          ----------------------------------------------------------------------
B.distachyon_1g          ----------------------------------------------------------------------
S.italica_4g275          ----------------------------------------------------------------------
S.italica_4g174          ----------------------------------------------------------------------
B.distachyon_1g          ----------------------------------------------------------------------
A.trichopoda_00          ----------------------------------------------------------------------
A.trichopoda_00          ----------------------------------------------------------------------
C.reinhardtii_0          ----------------------------------------------------------------------
C.subellipsoide          ----------------------------------------------------------------------
O.lucimarinus_T          ----------------------------------------------------------------------
C.merolae_TyrA           ----------------------------------------------------------------------
G.sulphuraria_T          ----------------------------------------------------------------------
S.cryophilus_Ty      335 PQKHMICSTPLFRLWVGVSEYVFCNPDLLTSCIYTATKHNEFCPDDLEFVISARSWSEHVEQGDFENYKK
P.decumbens_Tyr      330 PYDHMICSTPLFRLWLGVTEYLFRNPELLEEALDTAIDDNTFRSDDLEFTFAARAWSDCVSFGDFESYRD
A.carbonarius_T      330 PYDHMICSTPLFRLWLGVTEYLFRNPDLLDEALDTAVDDKTFRSDDLEFTFAARAWSDCVSFGDFESYRD
S.complicata_Ty      343 PYDHMICSTPLFRMWLGVTEYLFKQPQLLDECIRIALTDDTFRSDDLEFTFAARGWAECVGFGDFGSYRK
S.japonicus_Tyr      339 PQKQMICSTPLFRLWVGVAEYIFRQPEMLDRAIKVMLRQSDFCSDDLEFVIAARSWSDCVENGDFDTYKK
S.octosporus_Ty      335 PQKHMICSTPLFRLWVGVSEYVFCNPDLLTSCIYTATKHNEFCPDDLEFVLSARSWSEHVEQGDFENYKK
S.pombe_TyrA         328 PQNHMICSTPLFRLWVGVSEYVFRHPGLLDSCIYTATKHNDFSPDDLEFVVAVRSWSECVAAKDFTTYKK
```

```
s003                   ----------------------------------------------------------------------
S.spPCC6803_Tyr        ----------------------------------------------------------------------
B.animalis_TyrA        ----------------------------------------------------------------------
B.pseudocatenul        ----------------------------------------------------------------------
B.adolescentis_        ----------------------------------------------------------------------
B.dentium_TyrA         ----------------------------------------------------------------------
S.meliloti_TyrA        ----------------------------------------------------------------------
R.leguminosarum        ----------------------------------------------------------------------
T.sp_TyrA              ----------------------------------------------------------------------
D.invisus_TyrA         ----------------------------------------------------------------------
B.anthracis_Tyr        ----------------------------------------------------------------------
S.thermophilus_        ----------------------------------------------------------------------
A.aeolicus_TyrA        ----------------------------------------------------------------------
R.pneumotropicu        ----------------------------------------------------------------------
s001                   ----------------------------------------------------------------------
H.influenzae_Ty        ----------------------------------------------------------------------
A.succinogenes_        ----------------------------------------------------------------------
H.massiliensis_        ----------------------------------------------------------------------
H.somni_TyrA           ----------------------------------------------------------------------
A.actinomycetem        ----------------------------------------------------------------------
A.aphrophilus_T        ----------------------------------------------------------------------
N.archeti_TyrA         ----------------------------------------------------------------------
Y.kristensenii_        ----------------------------------------------------------------------
S.marcescens_Ty        ----------------------------------------------------------------------
E.coli_TyrA            ----------------------------------------------------------------------
C.youngae_TyrA         ----------------------------------------------------------------------
S.boydii_TyrA          ----------------------------------------------------------------------
C.freundii_TyrA        ----------------------------------------------------------------------
E.fergusonii_Ty        ----------------------------------------------------------------------
S.enterica_TyrA        ----------------------------------------------------------------------
C.sakazakii_Tyr        ----------------------------------------------------------------------
X.hominickii_Ty        ----------------------------------------------------------------------
P.asymbiotica_T        ----------------------------------------------------------------------
M.fervens_TyrA     396 IIDVYEGDNIEEGYKSITFRIYGYNKEELKNIEKEFLNIIKNIGGKERFK--------------------
M.maripaludis_T    395 VIDTYSGDKIDENELSATFRINSYEKDDLKTLEEIFNEIIKNIGGKLRY---------------------
M.jannaschii_Ty    397 IIDVYEGEKIEEGYKSITFRIYGYNKDELKNIEKEFLKIIKNIGGKERFK--------------------
M.vulcani_TyrA     388 SIEIIDRYEGVEGTERLSVTFRVMIIGDVEAEKVHLEVERQLKGIGCSIRGQ------------------
M.burtonii_TyrA    388 STEIIDTYSGISDNRLSATFRITILEESNATKMQTDVEKLLCGLGCKIRG--------------------
M.thermophila_T        ----------------------------------------------------------------------
M.concilii_TyrA        ----------------------------------------------------------------------
M.harundinacea_        ----------------------------------------------------------------------
M.barkeri_TyrA1    407 NAINTNKQLAACEISDIYKGVNQGDHKRINRGDYKEFNRIETEKEREEAKRLGVTYRITVFGDCDANSVE
M.barkeri_TyrA2    407 NAINTNKQLAACEISDMYKGINLGDHKRTNREYCKENWEDYKGNWGDYKGNWDNYKKFNLIGTKKEREET
M.horonobensis_    427 GAVSTNEQLADCQISDIYDEFNRTEPEKLICETGKLGVTYRITIFGDCNADSIESEVTTLLCGLGCRIRE
M.mazei_TyrA       397 RAVSTNKHLADCKISDIYKGVYETVLEKKGSTAEKLGVTYRIIIFGDCDADSVETEVTVLLCGLGCRIRE
C.methanoperede    387 SAQIIDVYNKQNMQSVTYRLTIFGDRDAAIVQGSVEKLLKGIGCTIR-----------------------
P.homiensis_Tyr        ----------------------------------------------------------------------
R.pomeroyi_TyrA        ----------------------------------------------------------------------
M.hallyeonensi_        ----------------------------------------------------------------------
J.pohangensis_T        ----------------------------------------------------------------------
R.halocynthiae_        ----------------------------------------------------------------------
P.ascidiaceicol        ----------------------------------------------------------------------
O.pituitosum_Ty        ----------------------------------------------------------------------
O.anthropi_TyrA        ----------------------------------------------------------------------
O.intermedium_T        ----------------------------------------------------------------------
B.canariense_Ty        ----------------------------------------------------------------------
P.inhibens_TyrA        ----------------------------------------------------------------------
P.zucineum_TyrA        ----------------------------------------------------------------------
S.spMCT13_TyrA         ----------------------------------------------------------------------
A.excentricus_T        ----------------------------------------------------------------------
S.cellulosum_Ty        ----------------------------------------------------------------------
M.xanthus_TyrA         ----------------------------------------------------------------------
C.bacteriumRBG_        ----------------------------------------------------------------------
D.mccartyi_TyrA        ----------------------------------------------------------------------
D.mccartyi_TyrA        ----------------------------------------------------------------------
D.spWBC-2_TyrA         ----------------------------------------------------------------------
D.lykanthropore        ----------------------------------------------------------------------
D.alkenigignens        ----------------------------------------------------------------------
A.sulfaticallid    395 EEIVRMVEKDENTYGLIPIENSVHGTVLKSIDALMRYDVEVFGETKMEVIHVLASKKKLELTEIEEVYSH
A.profundus_Tyr    385 DEIIRAVERGKVDYGLIPIENSINGTVLPVLDALLNSNVEVFGECELEVVHCLTAKREIPLKSVKVVYSH
A.veneficus_Tyr    385 DEIIKCVENGSADYGLVPIENSVNGTVISVLDALLNHDVEVFGETTLEIVHCLAARRYMPLKEIRVVYSH
G.ahangari_TyrA    384 EDIFLAVESGKADYGIVPIENSLQGTVLQTLDALLRHDVEVFAEYESEIRHNLVAKKRLPLRDIEVVYSH
F.placidus_TyrA    385 EEIVKAVESGKADFGILPIENSINGTVIPSLDALLKHDVEVFAETEVEVNLNLAAKKLIDLKDVEVIYSH
```

```
B.spBAL6_X_TyrA          ------------------------------------------------------------
B.spMedPE-Swde_          ------------------------------------------------------------
H.marinus_TyrA           ------------------------------------------------------------
B.bacterium_Tyr          ------------------------------------------------------------
D.bacterium4572          ------------------------------------------------------------
D.multivorans_T          ------------------------------------------------------------
D.bacterium_Tyr          ------------------------------------------------------------
D.cetonica_TyrA          ------------------------------------------------------------
D.tepidiphila_T          ------------------------------------------------------------
S.africana_TyrA          ------------------------------------------------------------
S.bajacaliforni          ------------------------------------------------------------
S.smaragdinae_T          ------------------------------------------------------------
S.bacterium_Tyr          ------------------------------------------------------------
S.bacteriumRBG_          ------------------------------------------------------------
S.odontotermiti          ------------------------------------------------------------
S.bacterium4572          ------------------------------------------------------------
C.Bathyarchaeot          ------------------------------------------------------------
M.truncatula_3g          ------------------------------------------------------------
s002                     ------------------------------------------------------------
G.max_11g233900          ------------------------------------------------------------
G.max_18g023100          ------------------------------------------------------------
B.bituminosa_20          ------------------------------------------------------------
P.vulgaris_1g24          ------------------------------------------------------------
L.sativus_20036          ------------------------------------------------------------
A.americana_205          ------------------------------------------------------------
M.truncatula_5g          ------------------------------------------------------------
G.max_14g055300          ------------------------------------------------------------
C.cajan_05579            ------------------------------------------------------------
P.trichocarpa_8          ------------------------------------------------------------
C.clementina_32          ------------------------------------------------------------
S.lycopersicum0          ------------------------------------------------------------
A.trichopoda_00          ------------------------------------------------------------
S.fallax_TyrA            ------------------------------------------------------------
S.lycopersicum0          ------------------------------------------------------------
A.trichopoda_00          ------------------------------------------------------------
P.patens_TyrA2           ------------------------------------------------------------
P.patens_TyrA1           ------------------------------------------------------------
A.thaliana_1g15          ------------------------------------------------------------
S.lycopersicum0          ------------------------------------------------------------
B.distachyon_1g          ------------------------------------------------------------
S.italica_4g275          ------------------------------------------------------------
S.italica_4g174          ------------------------------------------------------------
B.distachyon_1g          ------------------------------------------------------------
A.trichopoda_00          ------------------------------------------------------------
A.trichopoda_00          ------------------------------------------------------------
C.reinhardtii_0          ------------------------------------------------------------
C.subellipsoide          ------------------------------------------------------------
O.lucimarinus_T          ------------------------------------------------------------
C.merolae_TyrA           ------------------------------------------------------------
G.sulphuraria_T          ------------------------------------------------------------
S.cryophilus_Ty     405  QFIKIQDYFRPRFEEATKVGNAMIKKLLENLKKTA-------------------------
P.decumbens_Tyr     400  RFEKIASYFAPRFAEASKLGNEMMKTILEKTSNKP-------------------------
A.carbonarius_T     400  RFERIQQYFAPRFPEAVKLGNEMMKTILEKTTTGSA------------------------
S.complicata_Ty     413  RFEATQGFFEGRFEEATKIGNEMIATILAHTKD---------------------------
S.japonicus_Tyr     409  RFLKTKEYFRPRFEESRVVCNAMISKLMENLHK---------------------------
S.octosporus_Ty     405  QFVKIQDYFRPRFEEATKVGNAMIKKLLENLNKTA-------------------------
S.pombe_TyrA        398  RFLETQEYFRPRFEEATRVGNAMISKLLENLQKM--------------------------
```

```
s003                      ----------------------------------------------------------------------
S.spPCC6803_Tyr           ----------------------------------------------------------------------
B.animalis_TyrA           ----------------------------------------------------------------------
B.pseudocatenul           ----------------------------------------------------------------------
B.adolescentis_           ----------------------------------------------------------------------
B.dentium_TyrA            ----------------------------------------------------------------------
S.meliloti_TyrA           ----------------------------------------------------------------------
R.leguminosarum           ----------------------------------------------------------------------
T.sp_TyrA                 ----------------------------------------------------------------------
D.invisus_TyrA            ----------------------------------------------------------------------
B.anthracis_Tyr           ----------------------------------------------------------------------
S.thermophilus_           ----------------------------------------------------------------------
A.aeolicus_TyrA           ----------------------------------------------------------------------
R.pneumotropicu           ----------------------------------------------------------------------
s001                      ----------------------------------------------------------------------
H.influenzae_Ty           ----------------------------------------------------------------------
A.succinogenes_           ----------------------------------------------------------------------
H.massiliensis_           ----------------------------------------------------------------------
H.somni_TyrA              ----------------------------------------------------------------------
A.actinomycetem           ----------------------------------------------------------------------
A.aphrophilus_T           ----------------------------------------------------------------------
N.archeti_TyrA            ----------------------------------------------------------------------
Y.kristensenii_           ----------------------------------------------------------------------
S.marcescens_Ty           ----------------------------------------------------------------------
E.coli_TyrA               ----------------------------------------------------------------------
C.youngae_TyrA            ----------------------------------------------------------------------
S.boydii_TyrA             ----------------------------------------------------------------------
C.freundii_TyrA           ----------------------------------------------------------------------
E.fergusonii_Ty           ----------------------------------------------------------------------
S.enterica_TyrA           ----------------------------------------------------------------------
C.sakazakii_Tyr           ----------------------------------------------------------------------
X.hominickii_Ty           ----------------------------------------------------------------------
P.asymbiotica_T           ----------------------------------------------------------------------
M.fervens_TyrA            ----------------------------------------------------------------------
M.maripaludis_T           ----------------------------------------------------------------------
M.jannaschii_Ty           ----------------------------------------------------------------------
M.vulcani_TyrA            ----------------------------------------------------------------------
M.burtonii_TyrA           ----------------------------------------------------------------------
M.thermophila_T           ----------------------------------------------------------------------
M.concilii_TyrA           ----------------------------------------------------------------------
M.harundinacea_           ----------------------------------------------------------------------
M.barkeri_TyrA1      477  ADVTSLLCGLGCRIREKNLKQIEKTEPKR-----------------------------------------
M.barkeri_TyrA2      477  KRLGVTYRITVFGDCNASSVEADVTSLLCGLGCRIREKNLKQKGKPETERKI------------------
M.horonobensis_      497  KNLKYRE---------------------------------------------------------------
M.mazei_TyrA         467  KNLKFS----------------------------------------------------------------
C.methanoperede           ----------------------------------------------------------------------
P.homiensis_Tyr           ----------------------------------------------------------------------
R.pomeroyi_TyrA           ----------------------------------------------------------------------
M.hallyeonensi_           ----------------------------------------------------------------------
J.pohangensis_T           ----------------------------------------------------------------------
R.halocynthiae_           ----------------------------------------------------------------------
P.ascidiaceicol           ----------------------------------------------------------------------
O.pituitosum_Ty           ----------------------------------------------------------------------
O.anthropi_TyrA           ----------------------------------------------------------------------
O.intermedium_T           ----------------------------------------------------------------------
B.canariense_Ty           ----------------------------------------------------------------------
P.inhibens_TyrA           ----------------------------------------------------------------------
P.zucineum_TyrA           ----------------------------------------------------------------------
S.spMCT13_TyrA            ----------------------------------------------------------------------
A.excentricus_T           ----------------------------------------------------------------------
S.cellulosum_Ty           ----------------------------------------------------------------------
M.xanthus_TyrA            ----------------------------------------------------------------------
C.bacteriumRBG_           ----------------------------------------------------------------------
D.mccartyi_TyrA           ----------------------------------------------------------------------
D.mccartyi_TyrA           ----------------------------------------------------------------------
D.spWBC-2_TyrA            ----------------------------------------------------------------------
D.lykanthropore           ----------------------------------------------------------------------
D.alkenigignens           ----------------------------------------------------------------------
A.sulfaticallid      465  PQAMAQCAEFINNYLPKAKLRYTSSTSDAISMLKDTSAAIVSENAARLYNLYILRKGIQDMENNVTRFYI
A.profundus_Tyr      455  PQAIAQCINFINNYLPHAEIRYTTSTSDAIKLLDENSVAITSEYAARLYRLCILRKGIQDVKNNVTRFYL
A.veneficus_Tyr      455  PQAIAQCMGFINNYLKAELRYTSSTSDAIALLDDYSAAIVSENAAKLHKLYVLRKGIQDAKANTTRFYLI
G.ahangari_TyrA      454  PQAIAQCSEFINNYLPHAEIRYTRTTSEAIEMLDDRSAAIASELAAKLYRLQVIKRDIQSLTSNRTRFYI
F.placidus_TyrA      455  PQAVSQCMTFINNYLPHAEIRYTRSTSDAVSLLDEKSAAIVSENAAKIYRLQILRRSIQDAKENVTRFYV
```

```
B.spBAL6_X_TyrA      ----------------------------------------------------------------------
B.spMedPE-Swde_      ----------------------------------------------------------------------
H.marinus_TyrA       ----------------------------------------------------------------------
B.bacterium_Tyr      ----------------------------------------------------------------------
D.bacterium4572      ----------------------------------------------------------------------
D.multivorans_T      ----------------------------------------------------------------------
D.bacterium_Tyr      ----------------------------------------------------------------------
D.cetonica_TyrA      ----------------------------------------------------------------------
D.tepidiphila_T      ----------------------------------------------------------------------
S.africana_TyrA      ----------------------------------------------------------------------
S.bajacaliforni      ----------------------------------------------------------------------
S.smaragdinae_T      ----------------------------------------------------------------------
S.bacterium_Tyr      ----------------------------------------------------------------------
S.bacteriumRBG_      ----------------------------------------------------------------------
S.odontotermiti      ----------------------------------------------------------------------
S.bacterium4572      ----------------------------------------------------------------------
C.Bathyarchaeot      ----------------------------------------------------------------------
M.truncatula_3g      ----------------------------------------------------------------------
s002                 ----------------------------------------------------------------------
G.max_11g233900      ----------------------------------------------------------------------
G.max_18g023100      ----------------------------------------------------------------------
B.bituminosa_20      ----------------------------------------------------------------------
P.vulgaris_1g24      ----------------------------------------------------------------------
L.sativus_20036      ----------------------------------------------------------------------
A.americana_205      ----------------------------------------------------------------------
M.truncatula_5g      ----------------------------------------------------------------------
G.max_14g055300      ----------------------------------------------------------------------
C.cajan_05579        ----------------------------------------------------------------------
P.trichocarpa_8      ----------------------------------------------------------------------
C.clementina_32      ----------------------------------------------------------------------
S.lycopersicum0      ----------------------------------------------------------------------
A.trichopoda_00      ----------------------------------------------------------------------
S.fallax_TyrA        ----------------------------------------------------------------------
S.lycopersicum0      ----------------------------------------------------------------------
A.trichopoda_00      ----------------------------------------------------------------------
P.patens_TyrA2       ----------------------------------------------------------------------
P.patens_TyrA1       ----------------------------------------------------------------------
A.thaliana_1g15      ----------------------------------------------------------------------
S.lycopersicum0      ----------------------------------------------------------------------
B.distachyon_1g      ----------------------------------------------------------------------
S.italica_4g275      ----------------------------------------------------------------------
S.italica_4g174      ----------------------------------------------------------------------
B.distachyon_1g      ----------------------------------------------------------------------
A.trichopoda_00      ----------------------------------------------------------------------
A.trichopoda_00      ----------------------------------------------------------------------
C.reinhardtii_0      ----------------------------------------------------------------------
C.subellipsoide      ----------------------------------------------------------------------
O.lucimarinus_T      ----------------------------------------------------------------------
C.merolae_TyrA       ----------------------------------------------------------------------
G.sulphuraria_T      ----------------------------------------------------------------------
S.cryophilus_Ty      ----------------------------------------------------------------------
P.decumbens_Tyr      ----------------------------------------------------------------------
A.carbonarius_T      ----------------------------------------------------------------------
S.complicata_Ty      ----------------------------------------------------------------------
S.japonicus_Tyr      ----------------------------------------------------------------------
S.octosporus_Ty      ----------------------------------------------------------------------
S.pombe_TyrA         ----------------------------------------------------------------------
```

```
s003                 ----------------------------------------------------------------------
S.spPCC6803_Tyr      ----------------------------------------------------------------------
B.animalis_TyrA      ----------------------------------------------------------------------
B.pseudocatenul      ----------------------------------------------------------------------
B.adolescentis_      ----------------------------------------------------------------------
B.dentium_TyrA       ----------------------------------------------------------------------
S.meliloti_TyrA      ----------------------------------------------------------------------
R.leguminosarum      ----------------------------------------------------------------------
T.sp_TyrA            ----------------------------------------------------------------------
D.invisus_TyrA       ----------------------------------------------------------------------
B.anthracis_Tyr      ----------------------------------------------------------------------
S.thermophilus_      ----------------------------------------------------------------------
A.aeolicus_TyrA      ----------------------------------------------------------------------
R.pneumotropicu      ----------------------------------------------------------------------
s001                 ----------------------------------------------------------------------
H.influenzae_Ty      ----------------------------------------------------------------------
A.succinogenes_      ----------------------------------------------------------------------
H.massiliensis_      ----------------------------------------------------------------------
H.somni_TyrA         ----------------------------------------------------------------------
A.actinomycetem      ----------------------------------------------------------------------
A.aphrophilus_T      ----------------------------------------------------------------------
N.archeti_TyrA       ----------------------------------------------------------------------
Y.kristensenii_      ----------------------------------------------------------------------
S.marcescens_Ty      ----------------------------------------------------------------------
E.coli_TyrA          ----------------------------------------------------------------------
C.youngae_TyrA       ----------------------------------------------------------------------
S.boydii_TyrA        ----------------------------------------------------------------------
C.freundii_TyrA      ----------------------------------------------------------------------
E.fergusonii_Ty      ----------------------------------------------------------------------
S.enterica_TyrA      ----------------------------------------------------------------------
C.sakazakii_Tyr      ----------------------------------------------------------------------
X.hominickii_Ty      ----------------------------------------------------------------------
P.asymbiotica_T      ----------------------------------------------------------------------
M.fervens_TyrA       ----------------------------------------------------------------------
M.maripaludis_T      ----------------------------------------------------------------------
M.jannaschii_Ty      ----------------------------------------------------------------------
M.vulcani_TyrA       ----------------------------------------------------------------------
M.burtonii_TyrA      ----------------------------------------------------------------------
M.thermophila_T      ----------------------------------------------------------------------
M.concilii_TyrA      ----------------------------------------------------------------------
M.harundinacea_      ----------------------------------------------------------------------
M.barkeri_TyrA1      ----------------------------------------------------------------------
M.barkeri_TyrA2      ----------------------------------------------------------------------
M.horonobensis_      ----------------------------------------------------------------------
M.mazei_TyrA         ----------------------------------------------------------------------
C.methanoperede      ----------------------------------------------------------------------
P.homiensis_Tyr      ----------------------------------------------------------------------
R.pomeroyi_TyrA      ----------------------------------------------------------------------
M.hallyeonensi_      ----------------------------------------------------------------------
J.pohangensis_T      ----------------------------------------------------------------------
R.halocynthiae_      ----------------------------------------------------------------------
P.ascidiaceicol      ----------------------------------------------------------------------
O.pituitosum_Ty      ----------------------------------------------------------------------
O.anthropi_TyrA      ----------------------------------------------------------------------
O.intermedium_T      ----------------------------------------------------------------------
B.canariense_Ty      ----------------------------------------------------------------------
P.inhibens_TyrA      ----------------------------------------------------------------------
P.zucineum_TyrA      ----------------------------------------------------------------------
S.spMCT13_TyrA       ----------------------------------------------------------------------
A.excentricus_T      ----------------------------------------------------------------------
S.cellulosum_Ty      ----------------------------------------------------------------------
M.xanthus_TyrA       ----------------------------------------------------------------------
C.bacteriumRBG_      ----------------------------------------------------------------------
D.mccartyi_TyrA      ----------------------------------------------------------------------
D.mccartyi_TyrA      ----------------------------------------------------------------------
D.spWBC-2_TyrA       ----------------------------------------------------------------------
D.lykanthropore      ----------------------------------------------------------------------
D.alkenigignens      ----------------------------------------------------------------------
A.sulfaticallid  535 IRKQGSGEVDGNVTSLFFGVEDKPGALKDVLEVFYEKNINLRKLESRPSGTGLGDYIFFSEVEKRLNEDD
A.profundus_Tyr  525 IRRKGGKRSGRITAMFFGVEDRVGALKDVLEVFYKKNINMRKLESRPAKTGLGDYIFFVEIERALDEDDL
A.veneficus_Tyr  525 RKKGSGEMRGSITSLFFGVEDRPGALKDVLEVFYRKGINMRKLESRPARTGLGDYVFFVEVEKDLSSEDL
G.ahangari_TyrA  524 IRRSGGSVNGDESLTCLFFGVDDRPGALYRVLEVFYKNGINLRKLESRPAGTRLGDYIFFAEAEEKLGEN
F.placidus_TyrA  525 VRRRGGEVRGNVTALFFGVEDKPGALFSVLEIFKRRNINLRKLESRPSGIKLGDYIFFVEAEKKLEEEEL
```

```
B.spBAL6_X_TyrA      ----------------------------------------------------------------------
B.spMedPE-Swde_      ----------------------------------------------------------------------
H.marinus_TyrA       ----------------------------------------------------------------------
B.bacterium_Tyr      ----------------------------------------------------------------------
D.bacterium4572      ----------------------------------------------------------------------
D.multivorans_T      ----------------------------------------------------------------------
D.bacterium_Tyr      ----------------------------------------------------------------------
D.cetonica_TyrA      ----------------------------------------------------------------------
D.tepidiphila_T      ----------------------------------------------------------------------
S.africana_TyrA      ----------------------------------------------------------------------
S.bajacaliforni      ----------------------------------------------------------------------
S.smaragdinae_T      ----------------------------------------------------------------------
S.bacterium_Tyr      ----------------------------------------------------------------------
S.bacteriumRBG_      ----------------------------------------------------------------------
S.odontotermiti      ----------------------------------------------------------------------
S.bacterium4572      ----------------------------------------------------------------------
C.Bathyarchaeot      ----------------------------------------------------------------------
M.truncatula_3g      ----------------------------------------------------------------------
s002                 ----------------------------------------------------------------------
G.max_11g233900      ----------------------------------------------------------------------
G.max_18g023100      ----------------------------------------------------------------------
B.bituminosa_20      ----------------------------------------------------------------------
P.vulgaris_1g24      ----------------------------------------------------------------------
L.sativus_20036      ----------------------------------------------------------------------
A.americana_205      ----------------------------------------------------------------------
M.truncatula_5g      ----------------------------------------------------------------------
G.max_14g055300      ----------------------------------------------------------------------
C.cajan_05579        ----------------------------------------------------------------------
P.trichocarpa_8      ----------------------------------------------------------------------
C.clementina_32      ----------------------------------------------------------------------
S.lycopersicum0      ----------------------------------------------------------------------
A.trichopoda_00      ----------------------------------------------------------------------
S.fallax_TyrA        ----------------------------------------------------------------------
S.lycopersicum0      ----------------------------------------------------------------------
A.trichopoda_00      ----------------------------------------------------------------------
P.patens_TyrA2       ----------------------------------------------------------------------
P.patens_TyrA1       ----------------------------------------------------------------------
A.thaliana_1g15      ----------------------------------------------------------------------
S.lycopersicum0      ----------------------------------------------------------------------
B.distachyon_1g      ----------------------------------------------------------------------
S.italica_4g275      ----------------------------------------------------------------------
S.italica_4g174      ----------------------------------------------------------------------
B.distachyon_1g      ----------------------------------------------------------------------
A.trichopoda_00      ----------------------------------------------------------------------
A.trichopoda_00      ----------------------------------------------------------------------
C.reinhardtii_0      ----------------------------------------------------------------------
C.subellipsoide      ----------------------------------------------------------------------
O.lucimarinus_T      ----------------------------------------------------------------------
C.merolae_TyrA       ----------------------------------------------------------------------
G.sulphuraria_T      ----------------------------------------------------------------------
S.cryophilus_Ty      ----------------------------------------------------------------------
P.decumbens_Tyr      ----------------------------------------------------------------------
A.carbonarius_T      ----------------------------------------------------------------------
S.complicata_Ty      ----------------------------------------------------------------------
S.japonicus_Tyr      ----------------------------------------------------------------------
S.octosporus_Ty      ----------------------------------------------------------------------
S.pombe_TyrA         ----------------------------------------------------------------------
```

```
s003                   -------------------------------------------
S.spPCC6803_Tyr        -------------------------------------------
B.animalis_TyrA        -------------------------------------------
B.pseudocatenul        -------------------------------------------
B.adolescentis_        -------------------------------------------
B.dentium_TyrA         -------------------------------------------
S.meliloti_TyrA        -------------------------------------------
R.leguminosarum        -------------------------------------------
T.sp_TyrA              -------------------------------------------
D.invisus_TyrA         -------------------------------------------
B.anthracis_Tyr        -------------------------------------------
S.thermophilus_        -------------------------------------------
A.aeolicus_TyrA        -------------------------------------------
R.pneumotropicu        -------------------------------------------
s001                   -------------------------------------------
H.influenzae_Ty        -------------------------------------------
A.succinogenes_        -------------------------------------------
H.massiliensis_        -------------------------------------------
H.somni_TyrA           -------------------------------------------
A.actinomycetem        -------------------------------------------
A.aphrophilus_T        -------------------------------------------
N.archeti_TyrA         -------------------------------------------
Y.kristensenii_        -------------------------------------------
S.marcescens_Ty        -------------------------------------------
E.coli_TyrA            -------------------------------------------
C.youngae_TyrA         -------------------------------------------
S.boydii_TyrA          -------------------------------------------
C.freundii_TyrA        -------------------------------------------
E.fergusonii_Ty        -------------------------------------------
S.enterica_TyrA        -------------------------------------------
C.sakazakii_Tyr        -------------------------------------------
X.hominickii_Ty        -------------------------------------------
P.asymbiotica_T        -------------------------------------------
M.fervens_TyrA         -------------------------------------------
M.maripaludis_T        -------------------------------------------
M.jannaschii_Ty        -------------------------------------------
M.vulcani_TyrA         -------------------------------------------
M.burtonii_TyrA        -------------------------------------------
M.thermophila_T        -------------------------------------------
M.concilii_TyrA        -------------------------------------------
M.harundinacea_        -------------------------------------------
M.barkeri_TyrA1        -------------------------------------------
M.barkeri_TyrA2        -------------------------------------------
M.horonobensis_        -------------------------------------------
M.mazei_TyrA           -------------------------------------------
C.methanoperede        -------------------------------------------
P.homiensis_Tyr        -------------------------------------------
R.pomeroyi_TyrA        -------------------------------------------
M.hallyeonensi_        -------------------------------------------
J.pohangensis_T        -------------------------------------------
R.halocynthiae_        -------------------------------------------
P.ascidiaceicol        -------------------------------------------
O.pituitosum_Ty        -------------------------------------------
O.anthropi_TyrA        -------------------------------------------
O.intermedium_T        -------------------------------------------
B.canariense_Ty        -------------------------------------------
P.inhibens_TyrA        -------------------------------------------
P.zucineum_TyrA        -------------------------------------------
S.spMCT13_TyrA         -------------------------------------------
A.excentricus_T        -------------------------------------------
S.cellulosum_Ty        -------------------------------------------
M.xanthus_TyrA         -------------------------------------------
C.bacteriumRBG_        -------------------------------------------
D.mccartyi_TyrA        -------------------------------------------
D.mccartyi_TyrA        -------------------------------------------
D.spWBC-2_TyrA         -------------------------------------------
D.lykanthropore        -------------------------------------------
D.alkenigignens        -------------------------------------------
A.sulfaticallid   605  LRRLRDVTTFYRVAGIFREVDRIEL------------------
A.profundus_Tyr   595  KELKEVTTFYKIIGVFDRVESLTL--N----------------
A.veneficus_Tyr   595  RELRNVTTFYHIVGVFDRVERLDLWG-----------------
G.ahangari_TyrA   594  IVEEIKKRTTFCKVAGFFRKVDRLDVFTG--------------
F.placidus_TyrA   595  KELKERTTFYKVVGVFEKIDRLNVFISERKESSSSEDRRAKEA
```

```
B.spBAL6_X_TyrA      -------------------------------------------
B.spMedPE-Swde_      -------------------------------------------
H.marinus_TyrA       -------------------------------------------
B.bacterium_Tyr      -------------------------------------------
D.bacterium4572      -------------------------------------------
D.multivorans_T      -------------------------------------------
D.bacterium_Tyr      -------------------------------------------
D.cetonica_TyrA      -------------------------------------------
D.tepidiphila_T      -------------------------------------------
S.africana_TyrA      -------------------------------------------
S.bajacaliforni      -------------------------------------------
S.smaragdinae_T      -------------------------------------------
S.bacterium_Tyr      -------------------------------------------
S.bacteriumRBG_      -------------------------------------------
S.odontotermiti      -------------------------------------------
S.bacterium4572      -------------------------------------------
C.Bathyarchaeot      -------------------------------------------
M.truncatula_3g      -------------------------------------------
s002                 -------------------------------------------
G.max_11g233900      -------------------------------------------
G.max_18g023100      -------------------------------------------
B.bituminosa_20      -------------------------------------------
P.vulgaris_1g24      -------------------------------------------
L.sativus_20036      -------------------------------------------
A.americana_205      -------------------------------------------
M.truncatula_5g      -------------------------------------------
G.max_14g055300      -------------------------------------------
C.cajan_05579        -------------------------------------------
P.trichocarpa_8      -------------------------------------------
C.clementina_32      -------------------------------------------
S.lycopersicum0      -------------------------------------------
A.trichopoda_00      -------------------------------------------
S.fallax_TyrA        -------------------------------------------
S.lycopersicum0      -------------------------------------------
A.trichopoda_00      -------------------------------------------
P.patens_TyrA2       -------------------------------------------
P.patens_TyrA1       -------------------------------------------
A.thaliana_1g15      -------------------------------------------
S.lycopersicum0      -------------------------------------------
B.distachyon_1g      -------------------------------------------
S.italica_4g275      -------------------------------------------
S.italica_4g174      -------------------------------------------
B.distachyon_1g      -------------------------------------------
A.trichopoda_00      -------------------------------------------
A.trichopoda_00      -------------------------------------------
C.reinhardtii_0      -------------------------------------------
C.subellipsoide      -------------------------------------------
O.lucimarinus_T      -------------------------------------------
C.merolae_TyrA       -------------------------------------------
G.sulphuraria_T      -------------------------------------------
S.cryophilus_Ty      -------------------------------------------
P.decumbens_Tyr      -------------------------------------------
A.carbonarius_T      -------------------------------------------
S.complicata_Ty      -------------------------------------------
S.japonicus_Tyr      -------------------------------------------
S.octosporus_Ty      -------------------------------------------
S.pombe_TyrA         -------------------------------------------
```

SUPPLEMENTARY DATA S2 | Full amino acid alignment for all sequences used to created phylogenetic analyses.

An amino acid alignment of various TyrA orthologs from plants and microbes was created in PROMALS3D using TyrA crystal structures to guide the alignment. Identical amino acids present in >50% are shaded black, biochemically similar residues present in >50% of the sequences are shaded gray. Residues involved in the cofactor binding motif (GXGXXG) are boxed in blue. The cofactor determining position (e.g. 36, Bonner et al., 2008) is highlighted in red, though due to gaps in alignment at the N-terminus, position 36 is not entirely conserved in all sequences. The substrate specificity determining position (222, number based on GmPDH1) is highlighted in red. Some PDHs are bifunctional, thus there are extended C- or N-terminal regions in many of the TyrA enzymes.
