# Supplementary material for: Conserved Molecular Mechanism of TyrA Dehydrogenase Substrate Specificity Underlying Alternative Tyrosine Biosynthetic Pathways in Plants and Microbes
Source: Front Mol Biosci. 2017 Nov 7;4:73. doi: 10.3389/fmolb.2017.00073 (PMC5681985; doi:10.3389/fmolb.2017.00073)
Supplement: Supplementary file 3 [file Image1.pdf]

Supplementary Material

Conserved Molecular Mechanism of TyrA Dehydrogenase Substrate Specificity Underlying Alternative Tyrosine Biosynthetic Pathways in Plants and Microbes

Craig A. Schenck[1,2], Yusen Men[1], Hiroshi A. Maeda[1*]

[1]Department of Botany, University of Wisconsin-Madison, Madison, WI

[2]Current address: Department of Biochemistry and Molecular Biology, Michigan State University, East Lansing, MI

*** Correspondence:** Hiroshi A. Maeda: maeda2@wisc.edu

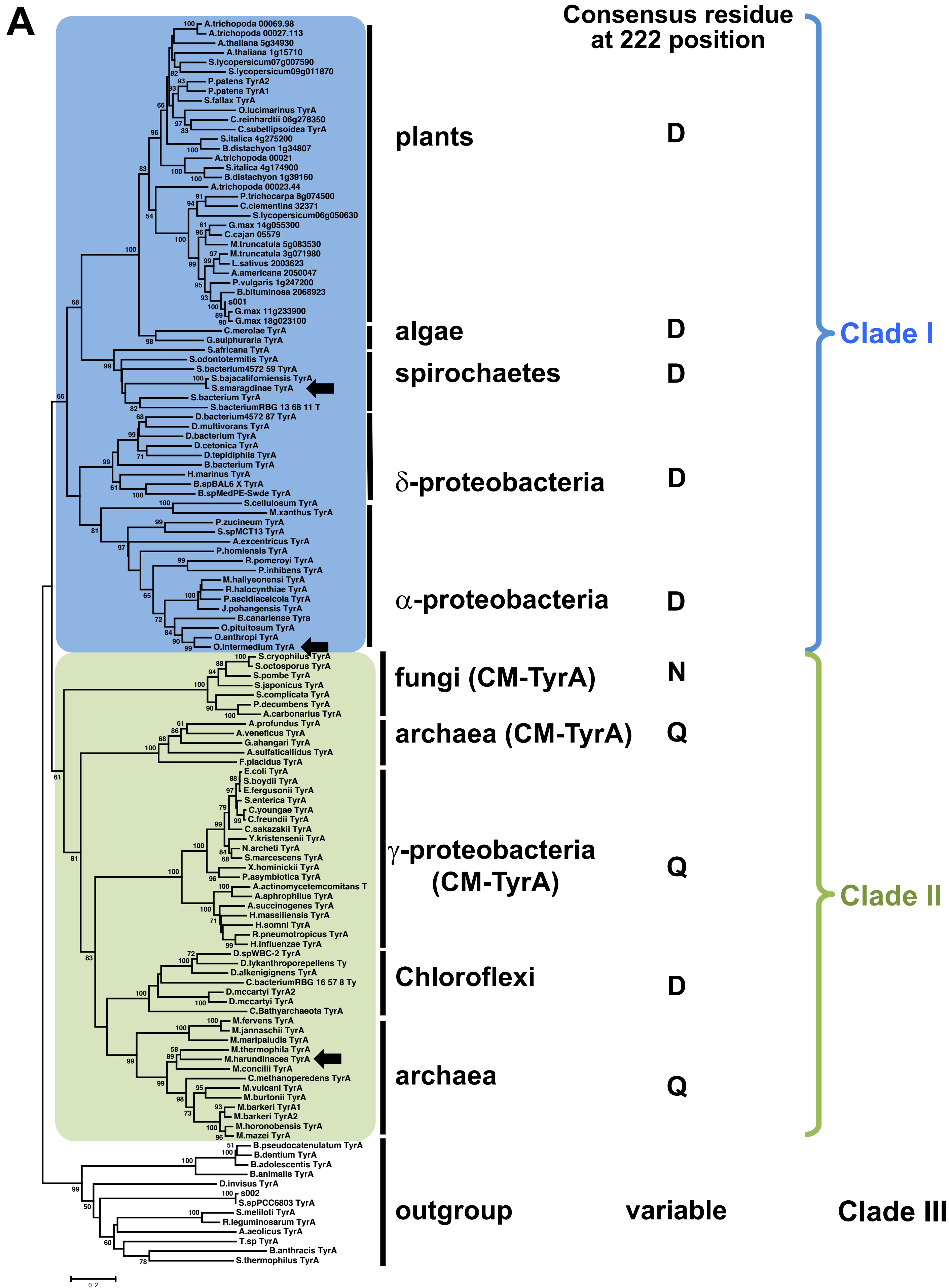
A
Consensus residue at 222 position
A.trichopoda 00069.98
A.trichopoda 00027.113
A.thaliana 5g34930
A.thaliana 1g15710
S.lycopersicum07g007590
S.lycopersicum09g011870
P.patens TyrA2
P.patens TyrA1
S.fallax TyrA
O.lucimarinus TyrA
C.reinhardtii 06g278350
C.subellipsoidea TyrA
S.italica 4g275200
B.distachyon 1g34807
A.trichopoda 00021
S.italica 4g174900
B.distachyon 1g39160
A.trichopoda 00023.44
P.trichocarpa 8g074500
C.clementina 32371
S.lycopersicum06g050630
G.max 14g055300
C.cajan 05579
M.truncatula 5g083530
M.truncatula 3g071980
L.sativus 2003623
A.americana 2050047
P.vulgaris 1g247200
B.bituminosa 2068923
s001
G.max 11g233900
G.max 18g023100
C.merolae TyrA
G.sulphuraria TyrA
S.africana TyrA
S.odontotermitis TyrA
S.bacterium4572 59 TyrA
S.bajacaliforniensis TyrA
S.smaragdinae TyrA
S.bacterium TyrA
S.bacteriumRBG 13 68 11 T
D.bacterium4572 87 TyrA
D.multivorans TyrA
D.bacterium TyrA
D.cetonica TyrA
D.tepidiphila TyrA
B.bacterium TyrA
H.marinus TyrA
B.spBAL6 X TyrA
B.spMedPE-Swde TyrA
S.cellulosum TyrA
M.xanthus TyrA
P.zucineum TyrA
S.spMCT13 TyrA
A.excentricus TyrA
P.homiensis TyrA
R.pomeroyi TyrA
P.inhibens TyrA
M.hallyeonensi TyrA
R.halocynthiae TyrA
P.ascidiaceicola TyrA
J.pohangensis TyrA
B.canariense Tyra
O.pituitosum TyrA
O.anthropi TyrA
O.intermedium TyrA
S.cryophilus TyrA
S.octosporus TyrA
S.pombe TyrA
S.japonicus TyrA
S.complicata TyrA
P.decumbens TyrA
A.carbonarius TyrA
A.profundus TyrA
A.veneficus TyrA
G.ahangari TyrA
A.sulfaticallidus TyrA
F.placidus TyrA
E.coli TyrA
S.boydii TyrA
E.fergusonii TyrA
S.enterica TyrA
C.youngae TyrA
C.freundii TyrA
C.sakazakii TyrA
Y.kristensenii TyrA
N.archeti TyrA
S.marcescens TyrA
X.hominickii TyrA
P.asymbiotica TyrA
A.actinomycetemcomitans T
A.aphrophilus TyrA
A.succinogenes TyrA
H.massiliensis TyrA
H.somni TyrA
R.pneumotropicus TyrA
H.influenzae TyrA
D.spWBC-2 TyrA
D.lykanthroporepellens Ty
D.alkenigignens TyrA
C.bacteriumRBG 16 57 8 Ty
D.mccartyi TyrA2
D.mccartyi TyrA
C.Bathyarchaeota TyrA
M.fervens TyrA
M.jannaschii TyrA
M.maripaludis TyrA
M.thermophila TyrA
M.harundinacea TyrA
M.concilii TyrA
C.methanoperedens TyrA
M.vulcani TyrA
M.burtonii TyrA
M.barkeri TyrA1
M.barkeri TyrA2
M.horonobensis TyrA
M.mazei TyrA
B.pseudocatenulatum TyrA
B.dentium TyrA
B.adolescentis TyrA
B.animalis TyrA
D.invisus TyrA
s002
S.spPCC6803 TyrA
S.meliloti TyrA
R.leguminosarum TyrA
A.aeolicus TyrA
T.sp TyrA
B.anthracis TyrA
S.thermophilus TyrA
0.2
plants D
algae D
spirochaetes D
δ-proteobacteria D
α-proteobacteria D
Clade I
fungi (CM-TyrA) N
archaea (CM-TyrA) Q
γ-proteobacteria (CM-TyrA) Q
Chloroflexi D
archaea Q
Clade II
outgroup variable
Clade III

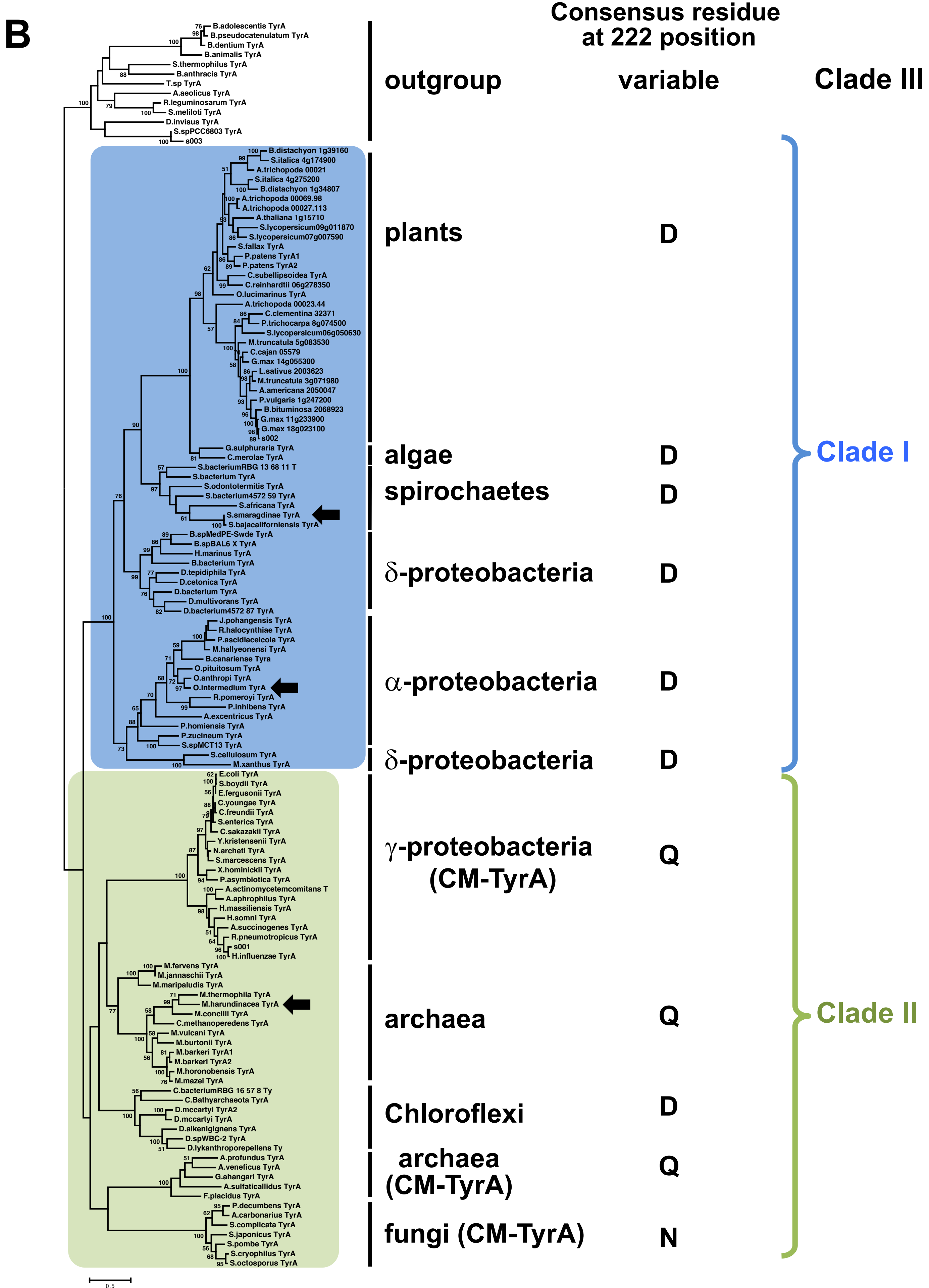

B
Consensus residue
at 222 position
outgroup
variable
Clade III
B.adolescentis TyrA
B.pseudocatenulatum TyrA
B.dentium TyrA
B.animalis TyrA
S.thermophilus TyrA
B.anthracis TyrA
T.sp TyrA
A.aeolicus TyrA
R.leguminosarum TyrA
S.meliloti TyrA
D.invisus TyrA
S.spPCC6803 TyrA
s003
B.distachyon 1g39160
S.italica 4g174900
A.trichopoda 00021
S.italica 4g275200
B.distachyon 1g34807
A.trichopoda 00069.98
A.trichopoda 00027.113
A.thaliana 1g15710
S.lycopersicum09g011870
S.lycopersicum07g007590
S.fallax TyrA
P.patens TyrA1
P.patens TyrA2
C.subellipsoidea TyrA
C.reinhardtii 06g278350
O.lucimarinus TyrA
A.trichopoda 00023.44
C.clementina 32371
P.trichocarpa 8g074500
S.lycopersicum06g050630
M.truncatula 5g083530
C.cajan 05579
G.max 14g055300
L.sativus 2003623
M.truncatula 3g071980
A.americana 2050047
P.vulgaris 1g247200
B.bituminosa 2068923
G.max 11g233900
G.max 18g023100
s002
plants
D
G.sulphuraria TyrA
C.merolae TyrA
algae
D
S.bacteriumRBG 13 68 11 T
S.bacterium TyrA
S.odontotermitis TyrA
S.bacterium4572 59 TyrA
S.africana TyrA
S.smaragdinae TyrA
S.bajacaliforniensis TyrA
spirochaetes
D
B.spMedPE-Swde TyrA
B.spBAL6 X TyrA
H.marinus TyrA
B.bacterium TyrA
D.tepidiphila TyrA
D.cetonica TyrA
D.bacterium TyrA
D.multivorans TyrA
D.bacterium4572 87 TyrA
δ-proteobacteria
D
J.pohangensis TyrA
R.halocynthiae TyrA
P.ascidiaceicola TyrA
M.hallyeonensi TyrA
B.canariense Tyra
O.pituitosum TyrA
O.anthropi TyrA
O.intermedium TyrA
R.pomeroyi TyrA
P.inhibens TyrA
A.excentricus TyrA
P.homiensis TyrA
P.zucineum TyrA
S.spMCT13 TyrA
α-proteobacteria
D
S.cellulosum TyrA
M.xanthus TyrA
δ-proteobacteria
D
Clade I
E.coli TyrA
S.boydii TyrA
E.fergusonii TyrA
C.youngae TyrA
C.freundii TyrA
S.enterica TyrA
C.sakazakii TyrA
Y.kristensenii TyrA
N.archeti TyrA
S.marcescens TyrA
X.hominickii TyrA
P.asymbiotica TyrA
A.actinomycetemcomitans T
A.aphrophilus TyrA
H.massiliensis TyrA
H.somni TyrA
A.succinogenes TyrA
R.pneumotropicus TyrA
s001
H.influenzae TyrA
γ-proteobacteria
(CM-TyrA)
Q
M.fervens TyrA
M.jannaschii TyrA
M.maripaludis TyrA
M.thermophila TyrA
M.harundinacea TyrA
M.concilii TyrA
C.methanoperedens TyrA
M.vulcani TyrA
M.burtonii TyrA
M.barkeri TyrA1
M.barkeri TyrA2
M.horonobensis TyrA
M.mazei TyrA
archaea
Q
C.bacteriumRBG 16 57 8 Ty
C.Bathyarchaeota TyrA
D.mccartyi TyrA2
D.mccartyi TyrA
D.alkenigignens TyrA
D.spWBC-2 TyrA
D.lykanthroporepellens Ty
Chloroflexi
D
A.profundus TyrA
A.veneficus TyrA
G.ahangari TyrA
A.sulfaticallidus TyrA
F.placidus TyrA
archaea
(CM-TyrA)
Q
P.decumbens TyrA
A.carbonarius TyrA
S.complicata TyrA
S.japonicus TyrA
S.pombe TyrA
S.cryophilus TyrA
S.octosporus TyrA
fungi (CM-TyrA)
N
Clade II

SUPPLEMENTARY FIGURE S1 | Phylogenetic analysis of plant and microbial TyrA orthologs. TyrA orthologs from various plants and microbes were identified through BlastP searches using characterized plant (AtADH2, GmPDH1) and microbial (*Synechocystis* sp. PCC6803 ADH, and *E. coli* PDH) TyrAs**.** Alignments were performed in PROMALS3D and guided by three TyrA structures from divergent organisms (GmPDH1, *Synechocystis* sp. PCC6803 ADH, and *H. influenzae* PDH). **(A)** Neighbor-joining phylogenetic analysis performed in MEGA7 from the PROMALS3D alignment of 130 TyrA orthologs. Evolutionary distances were calculated using the Poisson correction method with 1,000 bootstrap replicates, which are indicated at the branches with values less than 50% removed for clarity. Scale bar represents number of amino acid substitutions per site. **(B)** Phylogenetic analysis using the same sequences in **A** but inferred using Maximum-likelihood method with JTT matrix-based model. clade I, which contains all plant TyrAs is shaded in blue and the clade sister (clade II) is shaded in green. The consensus residue at the corresponding 222 position is shown to the right for each clade. Black arrows indicated enzymes characterized in this study.

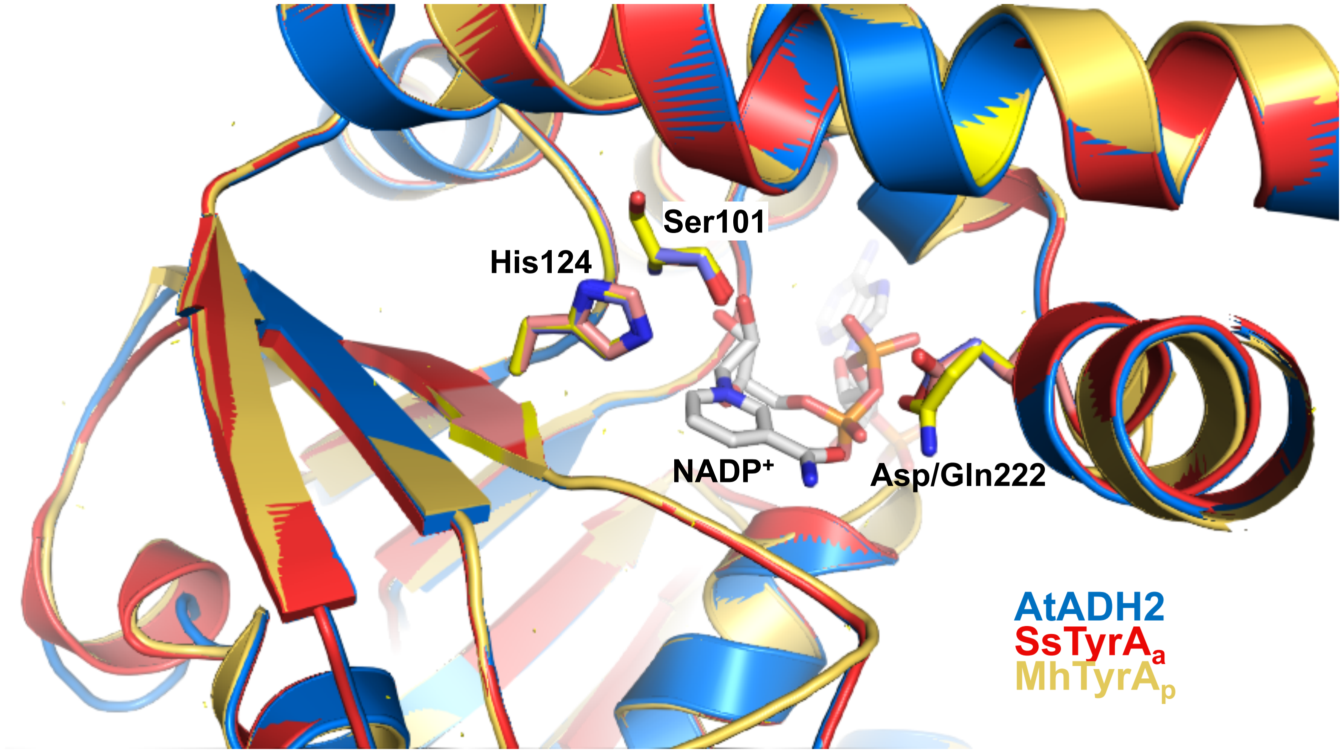

SUPPLEMENTARY FIGURE S2 | Structural conservation of residue 222 among clade I TyrA orthologs. Homology models of AtADH2 (blue), SsTyrA$_a$ (red), and MhTyrA$_p$ (yellow) show that they contain conserved catalytic residues (e.g. His and Ser, numbering based on GmPDH1 structure, which was used as the template for modeling). Clade I enzymes have an acidic residue at the active site 222: Asp in AtADH2 and SsTyrA, whereas Clade II, MhTyrA, has a Gln.

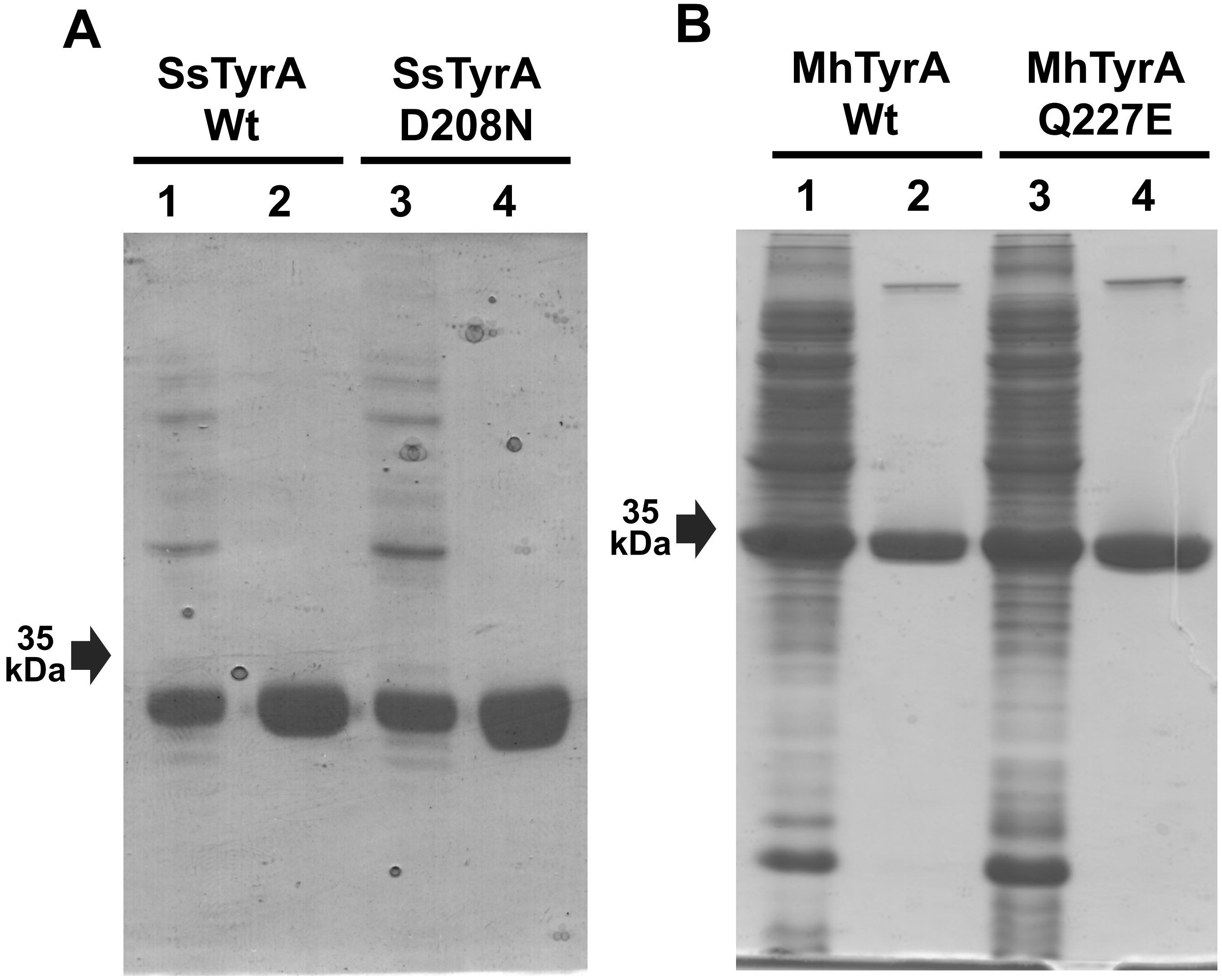

SUPPLEMENTARY FIGURE S3 | Purification of $SsTyrA_a$ and $MhTyrA_p$ Wild-type (Wt) and mutant recombinant enzymes.
(**A**) SDS-PAGE of supernatants and recombinant $SsTyrA_a$ Wt and D208N. *E. coli* supernatants (lanes 1 & 3) expressing $SsTyrA_a$ Wt and D208N were applied to a column containing Ni-NTA resin and eluted with 500mM imidazole containing buffer. Purified recombinant $SsTyrA_a$ Wt (lane 2) and D208N (lane 4) eluted at the appropriate size of ~30kDa. (**B**) SDS-PAGE of supernatants and recombinant $MhTyrA_p$ Wt and Q227E purified using affinity chromatography facilitated by a 6x-His tag on the N-terminus of the protein. *E. coli* supernatants (lanes 1 & 3) expressing MhTyrA Wt and Q227E were applied to a column containing Ni-NTA resin and eluted with 500mM imidazole containing buffer. Purified recombinant $MhTyrA_p$ Wt (lane 2) and Q227E (lane 4) eluted at the appropriate size of ~34kDa.

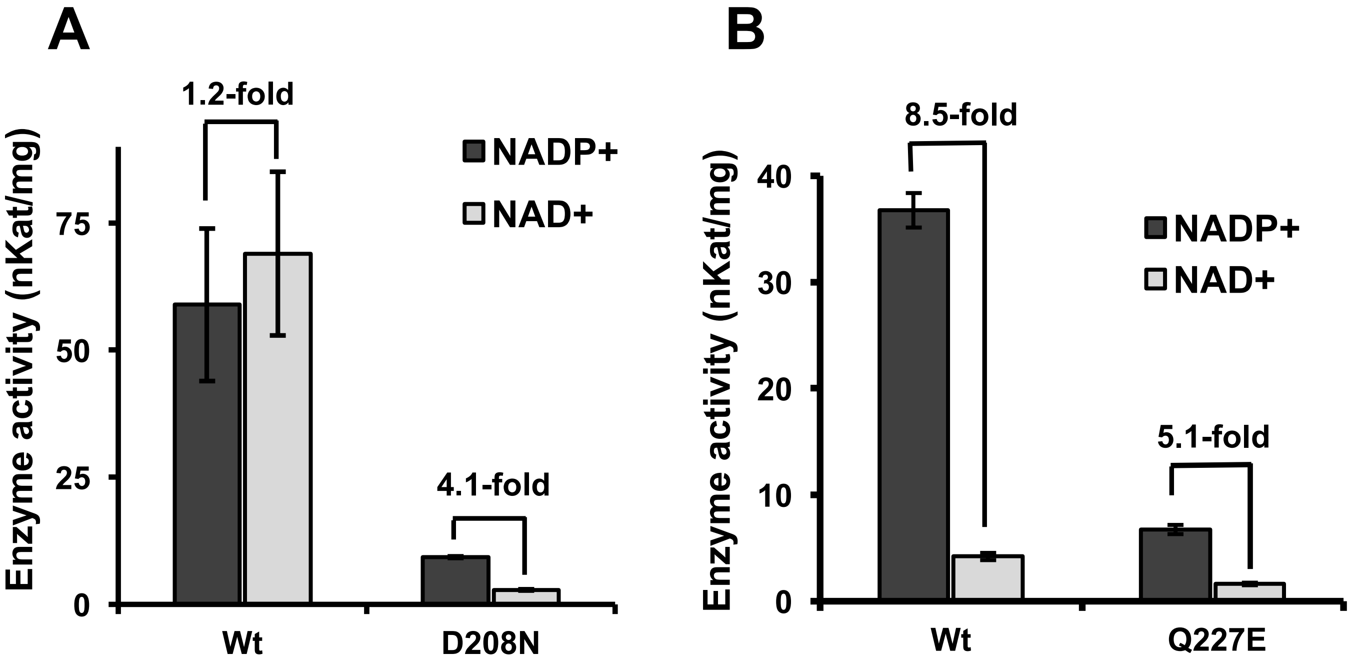

SUPPLEMENTARY FIGURE S4 | Cofactor specificity of SsTyrA$_a$ and D208N MhTyrA$_p$ Q227E mutants.

(**A**) The preferred substrate of SsTyrA$_a$ Wt and D208N mutant was used to test cofactor specificity with NADP$^+$ (black) and NAD$^+$ (gray). (**B**) The preferred substrate of MhTyrA$_p$ Wt and Q227E mutant was used to test cofactor specificity with NADP$^+$ (black) and NAD$^+$ (gray).

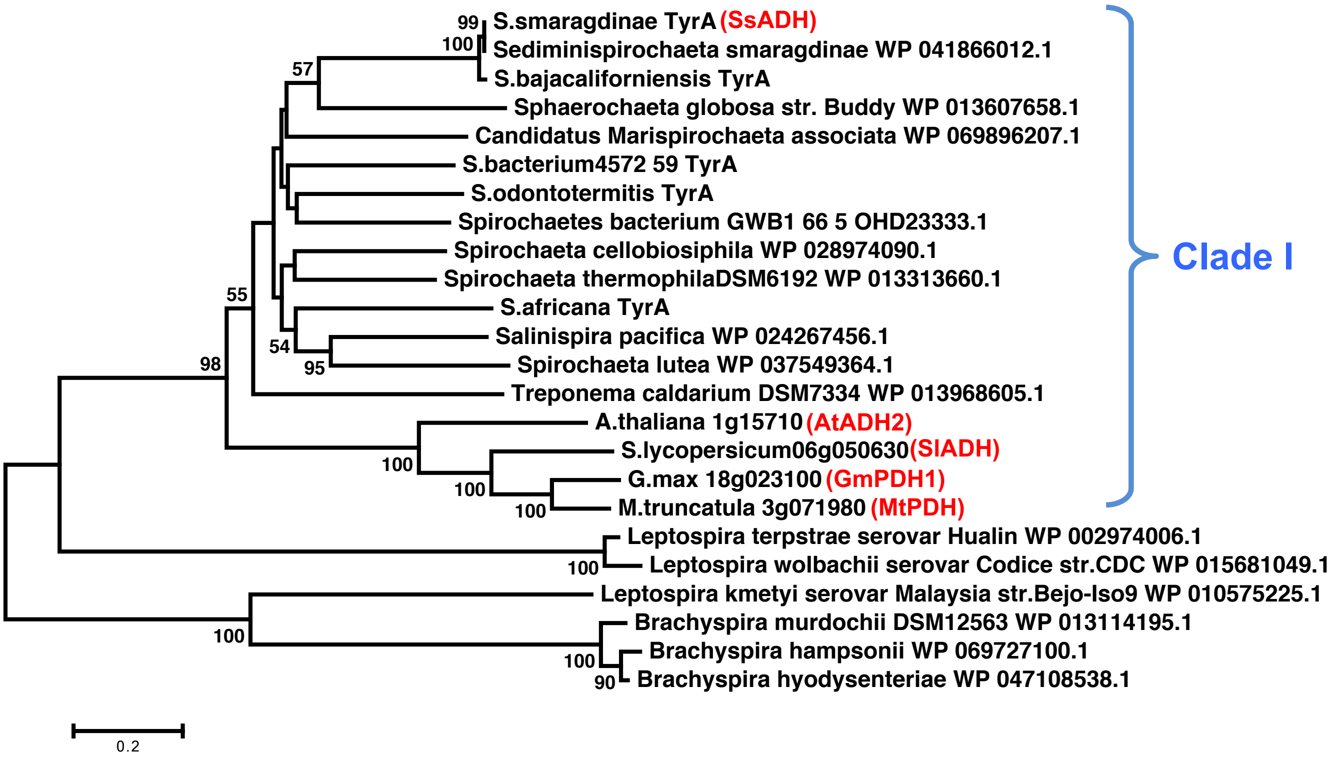

SUPPLEMENTARY FIGURE S5 | Phylogenetic analysis of Spirochaetes TyrA orthologs. TyrA orthologs from Spirochaetes were identified through BlastP searches using characterized Spirochaetes TyrA (SsADH) targeting specific Spirochaetes orders (Leptospirales, Brevinematales, and Brachyspirales) that were not included in **Figure 2** or Supplementary Figure S1. TyrA orthologs were identified in the Spirochaetes, Leptospirales, and Brachyspirales, but not in Brevinematales. Neighbor-joining phylogenetic analysis performed in MEGA7 from the MUSCLE alignment of Spirocheate TyrA orthologs. Evolutionary distances were calculated using the Poisson correction method with 1,000 bootstrap replicates, which are indicated at the branches, with values less then 50% removed for clarity. Scale bar represents number of amino acid substitutions per site. TyrA orthologs from Spirochaetes form a clade with SsADH and plant TryAs (characterized enzymes from this study or in previous studies shown in red). TyrA from Leptospirales and Brachyspirales group distinctly from clade I, suggesting that only a portion of Spirochaete have plant-like TyrA enzymes that group within clade I. Full genus and species followed by NCBI accession number are indicated for Spirochaete sequences not included in the original phylogenetic analyses (**Figure 2,** Supplementary Figure S1).

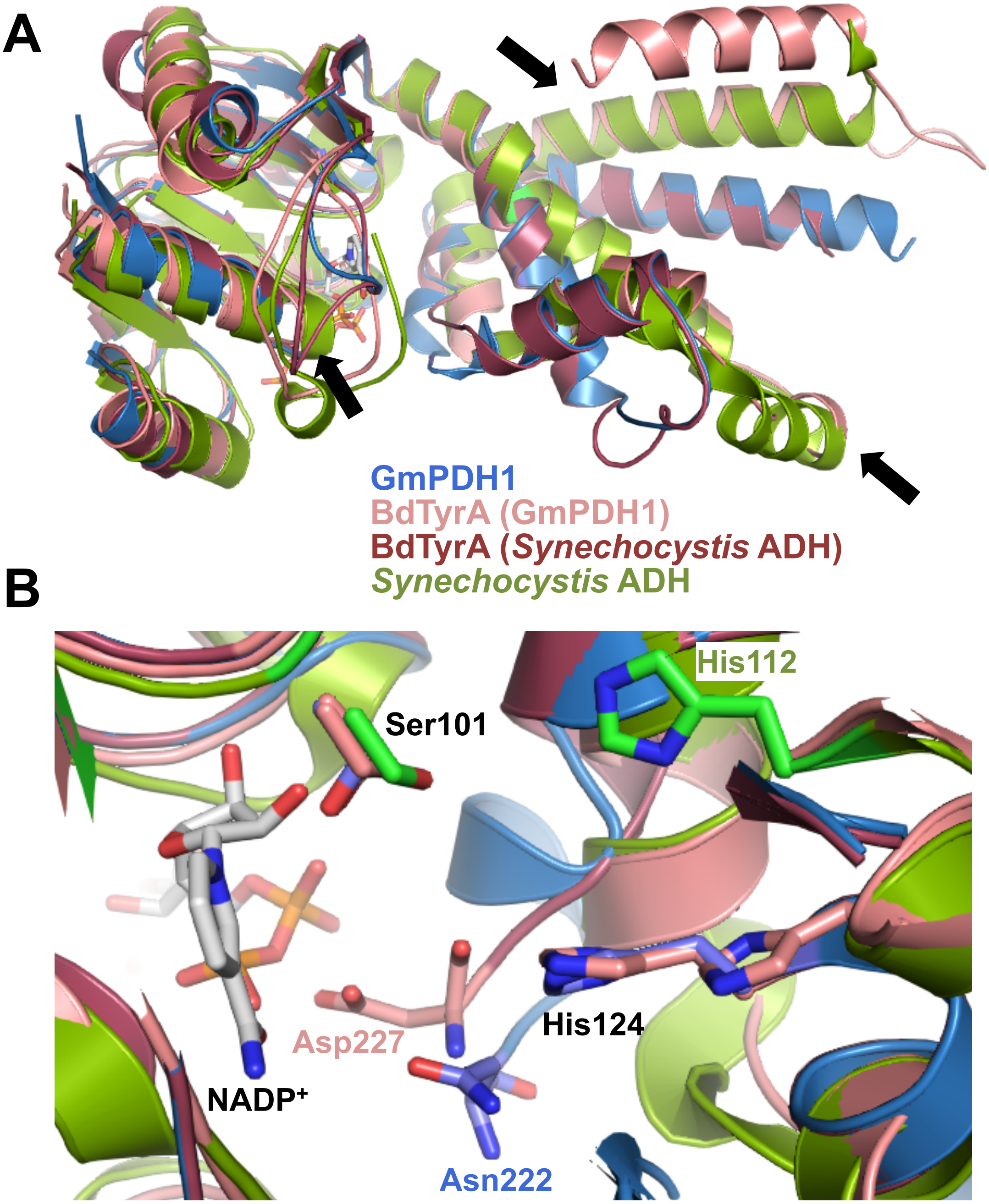

SUPPLEMENTARY FIGURE S6 | Conservation of global conformation in divergent microbial TyrA orthologs.

One representative sequence from the outgroup (*Bifidobacterium dentium*, BdTyrA) was chosen to determine active site architecture conservation in divergent microbial TyrAs. Homology models for BdTyrA (red), were created in SWISS-MODEL using GmPDH1 (light red; BdTyrA (GmPDH1)) and a more similar sequence from *Synechocystis* (dark red; BdTyrA (*Synechocystis* ADH)) as templates. **(A)** An overlay was created showing both BdTyrA models and their template structures. The overall conformation is generally conserved across divergent TyrAs, with some exceptions highlighted with arrows. An extended loop region is present in both models of BdTyrA and *Synechocystis*ADH, and there are additional α-helices in BdTyrA (*Synechocystis*) and *Synechocystis* ADH. **(B)** All enzymes possess the catalytic His and Ser residues, although His112 in *Synechocystis* ADH is in a slightly different position within the active site. The substrate specificity determining residue is present in only GmPDH1 (Asn222), whereas Asp227 in BdTyrA (GmPDH1) is shown but did not align with Asn222 in PROMALS3D alignments and adopts a different conformation and position than Asn222. In BdTyrA (*Synechocystis* ADH) and *Synechocystis* ADH a corresponding residue is lacking entirely in the active site.
